# Chemical Proteomics Reveals Human Off‐Targets of Fluoroquinolone Induced Mitochondrial Toxicity

**DOI:** 10.1002/anie.202421424

**Published:** 2025-04-02

**Authors:** Till Reinhardt, Yassmine El Harraoui, Alex Rothemann, Adrian T. Jauch, Sigrid Müller‐Deubert, Martin F. Köllen, Timo Risch, Lianne JHC Jacobs, Rolf Müller, Franziska R. Traube, Denitsa Docheva, Stefan Zahler, Jan Riemer, Nina C. Bach, Stephan A. Sieber

**Affiliations:** ^1^ Center for Functional Protein Assemblies Department of Bioscience TUM School of Natural Sciences Technische Universität München Ernst-Otto-Fischer-Straße 8 85748 Garching, Deutschland.; ^2^ Institute for Biochemistry and CECAD University of Cologne Köln, Deutschland.; ^3^ Department of Pharmacy Pharmaceutical Biology Ludwig-Maximilians-Universität München Butenandtstraße 5–13 81377 München, Deutschland.; ^4^ Department of Musculoskeletal Tissue Regeneration Orthopaedic Hospital König-Ludwig-Haus University of Würzburg 97076 Würzburg, Deutschland.; ^5^ Helmholtz Institute for Pharmaceutical Research Saarland (HIPS) Helmholtz Centre for Infection Research (HZI) and Saarland University Department of Pharmacy Campus Building E8.1 66123 Saarbrücken, Deutschland.; ^6^ Institut für Biochemie und Technische Biochemie Universität Stuttgart 70569 Stuttgart.

**Keywords:** Chemical proteomics, Fluoroquinolone, Human off-targets, Mitochondrial toxicity, Antibiotics

## Abstract

Fluoroquinolones (FQs) are an important class of potent broad‐spectrum antibiotics. However, their general use is more and more limited by adverse side effects. While general mechanisms for the fluoroquinolone‐associated disability (FQAD) have been identified, the underlying molecular targets of toxicity remain elusive. In this study, focusing on the most commonly prescribed FQs Ciprofloxacin and Levofloxacin, whole proteome analyses revealed prominent mitochondrial dysfunction in human cells, specifically of the complexes I and IV of the electron transport chain (ETC). Furthermore, global untargeted chemo‐proteomic methodologies such as photo‐affinity profiling with FQ‐derived probes, as well as derivatization‐free thermal proteome profiling, were applied to elucidate human protein off‐targets of FQs in living cells. Accordingly, the interactions of FQs with mitochondrial AIFM1 and IDH2 have been identified and biochemically validated for their contribution to mitochondrial dysfunction. Of note, the FQ induced ETC dysfunction *via* AIFM1 activates the reverse carboxylation pathway of IDH2 for rescue, however, its simultaneous inhibition further enhances mitochondrial toxicity. This off‐target discovery study provides unique insights into FQ toxicity enabling the utilization of identified molecular principles for the design of a safer FQ generation.

## Graphical abstract

A schematic overview of the adverse effects of Ciprofloxacin related to mitochondrial toxicity. AIFM1 plays a role in the MIA40‐mediated import into the IMS and oxidative folding of nuclear‐encoded proteins, including ETC complexes I and IV subunits. Ciprofloxacin disrupts the IMS import machinery, impairing ETC function. Dysfunction, especially of complex I, leads to a reduction in the NAD+/NADH ratio. This alteration in redox homeostasis, coupled with a shortage of NAD+, a crucial cofactor for the TCA cycle, can trigger metabolic reprogramming involving IDH2.

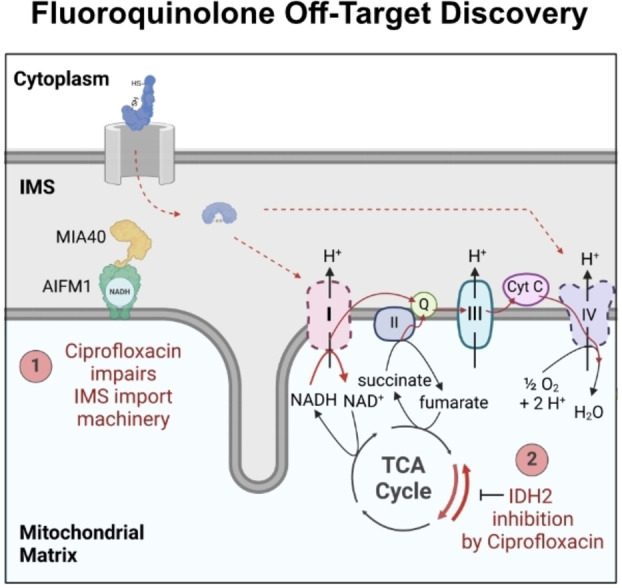

## Introduction

With the rapid development of multi‐resistant bacteria, the arsenal of effective antibiotics to treat infections is dramatically shrinking.[^1^, ^2^, ^3^] This crisis is further intensified by safety concerns with a class of front‐line antibiotics, the fluoroquinolones (FQs), which received several black box warnings by the US food and drug administration (FDA) due to severe side effects.[^4^, ^5^] A restricted use of FQs is in particular a drawback for the treatment of Gram‐negative strains which are only addressed by a limited number of alternative antibiotics capable of entering their almost impermeable cell membranes.^[6]^ Thus, to keep these desperately needed antibiotics within the first‐line of defense against pathogenic bacteria, it is crucial to decipher the unwanted off‐targets in human cells, to understand the origin of side effects and develop chemical strategies to enhance selectivity towards bacterial cells.

FQs inhibit the bacterial topoisomerase IV and gyrase, two enzymes essential for DNA replication.^[7]^ A crucial motif for the inhibition of both enzymes is the β‐keto acid moiety of FQs, which acts as a chelator of active‐site Mg^2+^ ions. The β‐keto acid of FQs binds *via* water‐metal ion bridges to highly‐conserved serine and acidic residues in the subunits A of the type‐II topoisomerases.^[8]^ Consequently, these residues are most frequently mutated in FQ‐resistant bacteria, defining the quinolone resistance‐determining region (QRDR).^[9]^

Although a large variety of FQs were developed, only a few are used in human therapy against bacterial infections. Safety concerns led to the withdrawal of various members, such as Temafloxacin in 1992 and Grepafloxacin in 1999.[^10^, ^11^] Currently still marketed and commonly used derivatives, such as Ciprofloxacin (Cipro) and Levofloxacin (Levo), are usually well‐tolerated, however, severe, disabling and potentially long‐lasting or permanent adverse effects are well documented.[^5^, ^12^, ^13^] Potential side‐effects include tendinopathy and tendon rupture,[^14^, ^15^] aortopathy,[^16^, ^17^] neuropathy,[^18^, ^19^] and various other adverse reactions, including CNS events.^[20]^ In 2015, the FDA termed this set of adverse effects the “Fluoroquinolone‐associated disability” (FQAD) and recommended to limit the use of FQs to infections insensitive to other alternative antibiotics.^[5]^ The reason for the increased sensitivity of some patients to FQs is not completely understood yet. A common theory is arising toxicity due to drug accumulation or concomitant prescriptions.[^20^, ^21^]

Various mechanistic studies have demonstrated that FQs are strong metal chelators, impairing different enzymatic reactions due to cofactor complexation, such as the inhibition of iron‐dependent dioxygenases, potentially leading to epigenetic effects.[^22^, ^23^] Likewise, integrin signaling, important for cell‐cell and cell‐extracellular matrix adhesion,^[24]^ and prolyl 4‐hydroxylases, involved in collagen maturation and modification,^[15]^ were reported to be affected *via* divalent ion coordination of FQs, suggesting multiple factors that could contribute to toxicity. Moreover, FQ treatment of human and animal tenocytes and human aortic myofibroblasts led to collagen degradation and increases in matrix metalloprotease (MMP) expression and activity.[^25^, ^26^] Both in tendons and ligaments, as well as in the aorta, collagen I and III are the major structural components.[^25^, ^27^] Mechanisms, such as mitochondrial dysfunction and increased oxidative stress are general consequences of FQ treatment.[^4^, ^12^, ^15^] Also, various other theories of how FQs lead to adverse effects have been proposed, including, but not limited to, GABA_A_ receptor inhibition, inhibition of mitochondrial topoisomerases, as well as α4β2 nicotinic acetylcholine receptor inhibition by Ciprofloxacin.[^28^, ^29^, ^30^]

**Figure 1:**
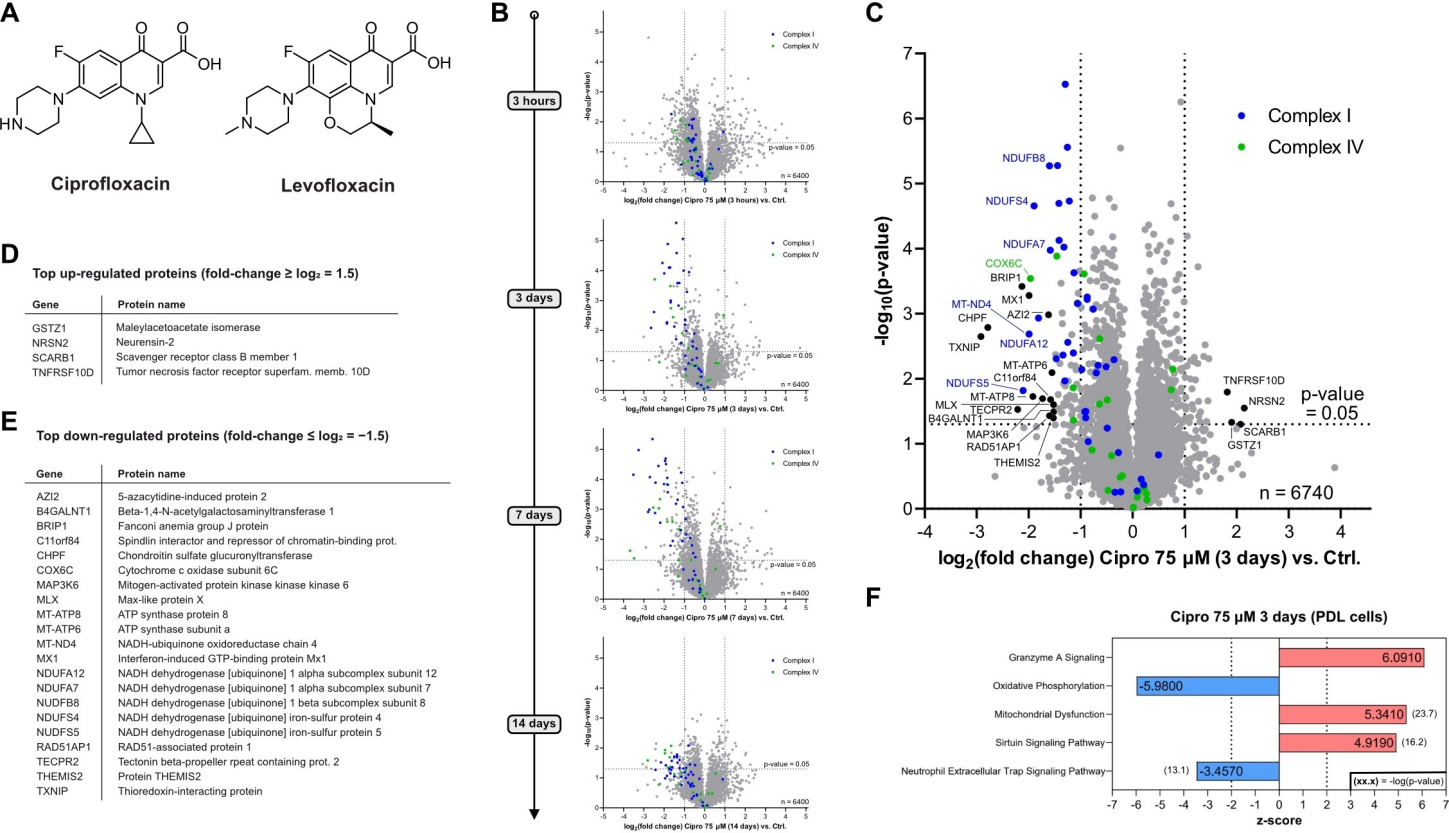
Whole proteome analysis of Ciprofloxacin‐treated human cells. (**A**) Structures of the FQs Ciprofloxacin and Levofloxacin. (**B**) Time‐dependent whole proteome analysis of Ciprofloxacin‐treated HEK‐293 cells (75 μM) versus control. Threshold lines represent a log_2_ regulation of ±1 and – log_10_ (*P*‐value) of 1.3 (two‐sided two‐sample *t*‐test, *n*=3 replicates per group). Subunits of the electron transport chain (ETC) complexes I and IV are depicted in blue and green, respectively. A pronounced and time‐dependent down‐regulation of both complexes was detected. Similar effects were observed for the reported plasma concentration (7.5 μM), however, shifted to later onset (see **Figure S2**). (**C**) Volcano plot depicting the proteome regulation of periodontal ligament cell line (PDL‐hTERT) treated with Ciprofloxacin (75 μM) for 3 days versus vehicle control‐treated cells featuring significant down‐regulation of complex I (blue) and complex IV (green) subunits of the ETC. Threshold lines represent a log_2_ regulation of ±1 and – log_10_ (*P*‐value) of 1.3 (two‐sided two‐sample *t*‐test, *n*=4 replicates per group). (**D**) Alphabetically sorted table of top up‐regulated proteins (threshold: log_2_ regulation≥1.5). (**E**) Alphabetically sorted table of top down‐regulated proteins (threshold: log_2_ regulation≤−1.5). (**F**) Top 5 most strongly regulated pathways (detailed pathway analysis in **Figure S6**).

Most reported studies focused on symptoms, indirect or downstream effects of FQ treatment and, to the best of our knowledge, no direct, unbiased and global off‐target identification study to decipher the fundamental molecular origins of FQ‐derived toxicity in human cells has been performed yet. To get insights into the molecular mechanisms of FQ‐associated adverse effects, we here focused on the most commonly prescribed FQs Ciprofloxacin and Levofloxacin (Figure 1A) and applied (chemical‐) proteomic methodologies to elucidate protein off‐targets in human cells. For this we selected a two‐tiered strategy based on thermal proteome profiling (TPP)^[31]^ as well as affinity‐based protein profiling (A*f*BPP)^[32]^ with tailored FQ‐derived photo‐crosslinker probes. Among the identified targets, two crucial mitochondrial enzymes isocitrate dehydrogenase isoform 2 (IDH2) and apoptosis‐inducing factor mitochondrial 1 (AIFM1), important for mitochondrial metabolism^[33]^ and respiratory chain biogenesis,[^34^, ^35^] respectively, were validated for FQ‐dependent effects. Corresponding whole proteome analysis and biochemical assays of short and long‐term FQ treatment confirmed major alterations in the respiratory chain complex I and IV as well as shifted NADPH levels corroborating the impaired cellular function of these target proteins. In light of rising antibiotic resistances and the dried‐up antibiotic pipeline, especially for Gram‐negative bacteria, this is of significant importance to develop new and safer FQ generations.

## Results and Discussion

### Whole Proteome Analysis of FQ‐treated Human Cells

To unravel pathways crucial for the onset of toxicity, we systematically investigated the changes in the proteome composition upon FQ addition *via* LC–MS/MS proteomics using label‐free quantification (LFQ).^[36]^ In pilot studies performed with Ciprofloxacin in HEK‐293 cells we first tested high (75 μM, resembling peak plasma levels) and low (7.5 μM, resembling regular plasma levels) concentrations of the drug co‐incubated with cells for 3 hours, 3 days, 7 days and 2 weeks representing a typical therapeutic regime (Figure 1B, S1, S2).[^37^, ^38^] Interestingly, both high and low concentrations evoked the significant up‐regulation of proteins involved in iron transport and homeostasis (HMOX1, HBA1, HPX, LTF),[^39^, ^40^, ^41^] cellular stress (HMOX1, AATF, GPX3, MST1, PEX10, VNN1)[^42^, ^43^, ^44^, ^45^, ^46^, ^47^] and collagen fibril organization (COL18A1, COL1A1, COL1A2, COL1A5, FMOD, FN1, ITGA‐1)[^48^, ^49^, ^50^] already after 3 h (Figure S1). The functions of these proteins correlate well with the known adverse effects of Ciprofloxacin to engage in metal‐ion complexation, enhancing cellular stress, e.g. *via* reactive oxygen species (ROS) and leading to tendon and ligament damage.[^12^, ^25^, ^51^] Interestingly, multiple target gene products and proteins involved in NF‐κB signaling were detected to be upregulated within the thresholds (fold‐change≥1, –log(*P*‐value)≥1.3; see Figure S1).[^52^, ^53^, ^54^] Following the time course further, we observed the downregulation of proteins relevant for the electron transport chain (ETC), crucial for oxidative phosphorylation, especially of complex I and of complex IV (Figure 1B, S2). At high concentrations, the downregulation already started at 3 h and was strongly pronounced after 3 days, peaking after 7 days (Figure 1B, S2B). Incubation of cells with 7.5 μM Ciprofloxacin showed a weaker shift of complex I and IV subunits after 3 and 7 days, but importantly after 14 days the downregulation became more prominent (Figure S2A). The extent and identity of the top regulated protein pathways after 3 days at 75 μM and 14 days at 7.5 μM was comparable, with strongly impaired oxidative phosphorylation, enhanced mitochondrial dysfunction and pronounced granzyme A and sirtuin signaling, e.g. involved in inflammation and redox homeostasis (Figure S3).[^55^, ^56^]

As tendon/ligament‐derived cells more closely represent the physiological environment of these tissues and ideally provide additional insights into the origin of FQ‐mediated tendon rupture, we performed similar whole proteome studies with human periodontal ligament (PDL) cells. We used as a model system an established cell line, namely PDL‐hTERT, which was immortalized *via* stable expression of human telomerase reverse transcriptase.^[57]^ We here focused on the 3‐day time point at high and low Ciprofloxacin concentrations and additionally performed offline cation‐exchange chromatography fractionation to achieve deeper proteome coverage (Figure 1C–E, **S4**). Interestingly, similar effects were recorded as in our previous analysis in HEK‐293 cells. In order to investigate if these regulated proteins are a general hallmark of FQs, we performed the same study with Levofloxacin which revealed a large fraction of comparable changes (Figure S5). Despite the deviating structure of Levofloxacin, the downregulation of complex I and IV was also observed using the same concentrations, although to a lesser extent. Of note, plasma concentrations of Levofloxacin are reported to be higher compared to Ciprofloxacin.^[37]^ For both Ciprofloxacin and Levofloxacin the top regulated protein pathways in PDL‐hTERT are consistent with the results in HEK‐293 cells (Figure 1F, S3, S6).

The observed proteome changes, e.g. the significant down‐regulation of the ETC complexes I and IV by Ciprofloxacin and Levofloxacin are in line with the literature‐known mitochondrial toxicity of fluoroquinolones.[^4^, ^12^, ^15^] However, Ciprofloxacin and Levofloxacin do not exert high general cytotoxicity as judged by the metabolic activity of HEK‐293 and PDL‐hTERT cells *via* MTT assays after 3 days of treatment with approximate apparent IC_50_ values well above the reported plasma concentrations (Figure S7). Although FQs have been reported to induce apoptosis at levels exceeding the plasma and peak concentrations, our FACS‐based cell‐death assays using the standard 7.5 μM and 75 μM peak concentrations of Ciprofloxacin and Levofloxacin in PDL‐hTERT cells for 3 days, did not reveal a significantly higher population of early‐ or late‐stage apoptotic cells compared to control cells, although prolonged treatment for 7 days led to a rounded cell morphology (Figure S8).^[58]^ We thus conclude that FQs do not exhibit acute cell toxicity and primarily a different mode of action must be responsible for the observed mitochondrial toxicity, which rather develops over a longer time of drug treatment and potentially accumulation.

### Synthesis and Bioactivity of Versatile FQ‐Based Profiling Tools

**Figure 2:**
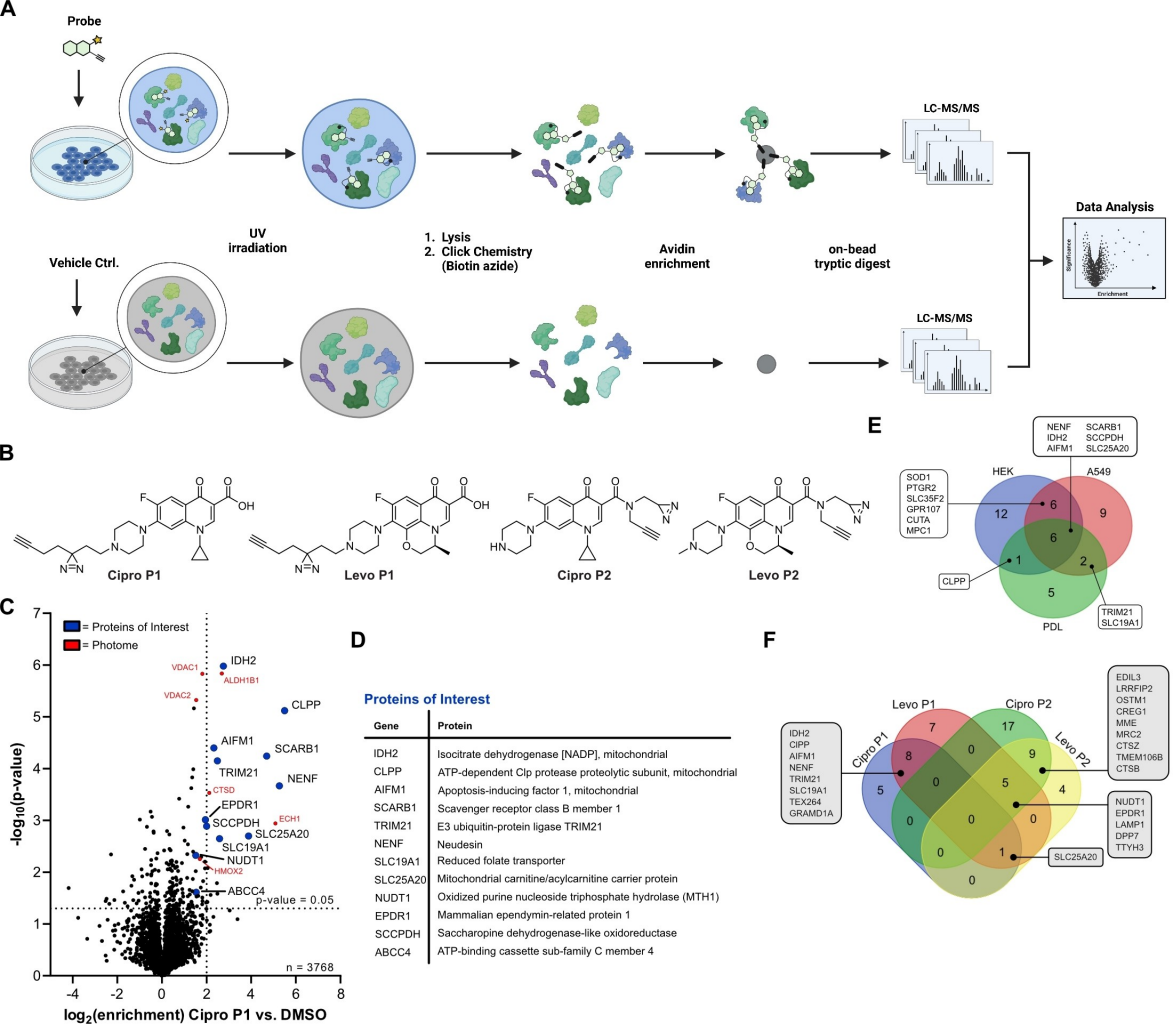
Fluoroquinolone off‐target discovery *via* affinity‐based protein profiling (A*f*BPP). (**A**) Schematic workflow of MS‐based A*f*BPP. Cells are incubated with affinity probe or vehicle control and subsequently irradiated with UV light to covalently link potential target proteins. After lysis, biotin is appended to the alkyne handle *via* CuAAC chemistry and engaged proteins enriched on avidin beads. Following tryptic digest, the resulting peptides are measured *via* LC–MS/MS and raw data analyzed to infer potential protein targets of the probe. (**B**) Set of FQ‐derived affinity probes based on Ciprofloxacin and Levofloxacin. (**C**) Representative A*f*BPP volcano plot of **Cipro P1** (2.5 μM, 1 h, 37 °C) in PDL‐hTERT cells versus the DMSO control. Threshold lines indicate log_2_ enrichment≥2 and the statistical significance –log10(*P* value)≥1.3 (two‐sided two‐sample *t*‐test, *n*=4 replicates per group). Literature‐known unspecific photome hits are depicted in red.^[65]^ Proteins of interest are highlighted in blue. (**D**) Corresponding table of potential off‐target proteins of **Cipro P1** in PDL cells. (**E**) Venn‐diagram illustrating the overlapping enriched proteins of **Cipro P1** in HEK293, A549 and PDL‐hTERT cell lines. (**F**) Venn‐diagram depicting the overlapping protein hits in PDL‐hTERT cells across the four FQ‐derived probes. Within the **P1** and **P2** probe sets putative protein (off−)targets are strongly conserved (8 shared hits with **P1** probes, 9 shared hits with **P2** probes). The overlap between **Levo P1** and both **P2** probes is 5 proteins. No protein is adhering to the standard thresholds across all probes.

To directly identify protein off‐targets of Ciprofloxacin and Levofloxacin, we designed chemical probes suitable for *in situ* protein target identification studies *via* A*f*BPP (Figure 2A, B).^[32]^ In order to enrich and identify non‐covalently binding proteins, a diazirine photo‐crosslinker together with an alkyne tag was incorporated in the core FQ‐scaffold. The piperazine ring, present in both drugs, was selected for modification with the minimal photo‐crosslinker as previous structure–activity relationship studies (SAR) for antibiotic activity in bacteria indicated flexibility in the modification of the amine at position C4.[^59^, ^60^] Alternatively, we modified the acid moiety with a photo‐crosslinker to account for a putative different binding mode of human protein off‐targets and maximize the chances of their identification. Appending the photo‐crosslinker at the carboxylic acid also impairs the well‐established bivalent metal complexation of FQs and thus could help to decipher off‐targets unrelated to metal binding.

Probe **Cipro P1** was synthesized using substitution conditions with Ciprofloxacin and the literature known iodo‐derivative of the minimalist photo‐crosslinker (Scheme S1A).^[61]^ The corresponding Levofloxacin probe **Levo P1** was synthesized starting from Levofloxacin Q‐acid. After nucleophilic aromatic substitution to install the piperazine moiety, the minimalist photo‐crosslinker was appended accordingly (Scheme S1B). Amide coupling of the branched photo‐affinity handle developed by *Conway et al*. to *N*‐Boc‐protected Ciprofloxacin and subsequent deprotection afforded **Cipro P2** (Scheme S2A).^[62]^ Similar amide coupling conditions starting with Levofloxacin afforded **Levo P2** (Scheme S2B).

The probes were first tested for their antibiotic activity against pathogenic *Staphylococcus aureus* (NCTC 8325) and *Escherichia coli* (MM28, Figure S9A). As expected, both **P2**‐probes with the derivatized acid moiety, that is essential for antibiotic activity, showed significant (400‐ to >1600‐fold) decreases in their minimal inhibitory concentrations (MICs) in Gram‐positive *S. aureus* and Gram‐negative *E. coli*. Satisfyingly, probes **Cipro P1** and **Levo P1** displayed only a 12.5‐ to 25‐fold reduction in the MIC in *S. aureus* suggesting only slight perturbations in the uptake and/or target engagement. In *E. coli*, **P1**‐probes exhibited a 100‐fold potency drop compared to their parent compound, which is most likely due to limited uptake, as the activities in the LPS‐deficient *E. coli* strain RFM795 only decreased 4‐fold.

To further confirm this notion, we exemplarily tested inhibition of one of the *S. aureus* cognate targets, the topoisomerase IV, with **Cipro P1** and an approx. 16‐fold reduction in activity compared to the parent Ciprofloxacin (IC_50_ of approx. 25 μM, in line with literature values of 7–20 μM) was observed (Figure S9B).[^63^, ^64^] Although the focus of this study is on human off‐targets, these results confirm the desired effects of the **P1**‐probes in bacteria which is a promising starting point for human off‐target discovery. For the same reason, also **P2**‐probes, despite showing no antibiotic activity, were subjected to the following human cell A*f*BPP experiments.

We thus shifted our attention to human cell lines and the corresponding toxic effects evoked by the probe molecules. MTT assays were performed in HEK‐293 and PDL‐hTERT cells after 3 d of co‐incubation and approximate apparent IC_50_ values ranged from 120 to 950 μM demonstrating no acute cell toxicity with the probes and therefore enabling their use for off‐target studies in human cells (Figure S10).

### Target Identification *via* Chemical Proteomics

With the unique insight of proteome changes, we next focused on the identification of the corresponding molecular targets responsible for these effects utilizing our FQ‐derived probes by A*f*BPP. We initiated these experiments by optimizing the probe concentration and duration of labeling *via* a fluorescent gel‐based readout using **Cipro P1** as proof‐of‐concept. Intact HEK‐293 cells were treated with various concentrations of the **Cipro P1** probe ranging from 2 to 75 μM for 1 h at 37 °C in FCS‐free medium. After this co‐incubation, the cells were irradiated for 15 min at 360 nm, lysed and reacted with TAMRA‐azide *via* copper(I)‐catalyzed azide/alkyne cycloaddition (CuAAC) chemistry.[^66^, ^67^] After separation *via* SDS‐PAGE, fluorescent scanning revealed concentration‐dependent labeling starting already at 2 μM (Figure S11).

Subsequently, we performed the respective quantitative analysis using biotin azide for avidin bead enrichment of probe‐targeted proteins and tryptic digest followed by LC–MS/MS analysis using label‐free quantification (LFQ) (Figure 2A).^[36]^ In MS‐based A*f*BPP, the comparison of proteins in probe‐treated and untreated cells provides enrichment ratios and significance values facilitating the ranking of hits (*P*‐value <0.05, fold‐change>log_2_ 2). However, care must be taken with common photo‐crosslinker off‐targets (photome hits), which have been previously inventoried for human cell lines.^[65]^ In order to narrow down promising targets, we performed concentration‐dependent labeling (0.5, 1.0 and 2.5 μM) with an optimal irradiation time of 10 min (Figure S12).

After exclusion of 4 known photome hits, 12 putative proteins of interest remained, featuring concentration‐dependent enrichment (Figure S12B–D). Interestingly, almost the same scope of protein hits was also observed in A549 cells suggesting a conserved set of targets within these different cell lines (Figure S13). With those promising initial results for **Cipro P1**, we again shifted our attention to PDL‐hTERT cells as our model system for tendons/ligaments. For these studies, we applied our full set of diversified **Cipro P1**, **Cipro P2** as well as **Levo P1** and **Levo P2** probes. Gel‐based studies revealed an optimal labeling concentration of 5 μM with the **P1**‐probes and 25 μM for the **P2**‐probes attributed to the branched photo‐crosslinker, which was reported to require higher concentrations for sufficient labeling (Figure S14).^[62]^ A*f*BPP of **Cipro P1** in PDL‐hTERT cells revealed a strong overlap of 6 proteins which are in common with the respective experiment in HEK‐293 and A549 cells and three proteins that were solely enriched in PDL‐hTERT cells (Figure 2C–E, **S15**). Despite the structural differences between their cyclic scaffolds, **Cipro P1** and **Levo P1** enriched a mutual set of 8 proteins including ClpP, AIFM1, NENF, TRIM21, SLC19A1, TEX264, GRAMD1A and IDH2 (**Figure S15, S16**). Importantly, largely different protein hits were obtained with **Cipro P2** and **Levo P2**, with 9 shared hits between both and no common overlap of the **P2**‐ with the **P1**‐ probes, suggesting a significant effect on the labeling specificity by de‐functionalizing the free acid (Figure 2F, S16). Interestingly, a majority of targets are lysosomal proteins suggesting that the modification of the free acid enhances the direction or trapping of the scaffold into the acidic lysosomal compartment **(Figure S16**). This notion was confirmed by co‐localization of **Cipro‐P2** with LAMP1 in the lysosome *via* immuno‐cytochemistry corroborating previous reports about lysosomotropic effects of Ciprofloxacin (Figure S17).^[58]^

**Figure 3:**
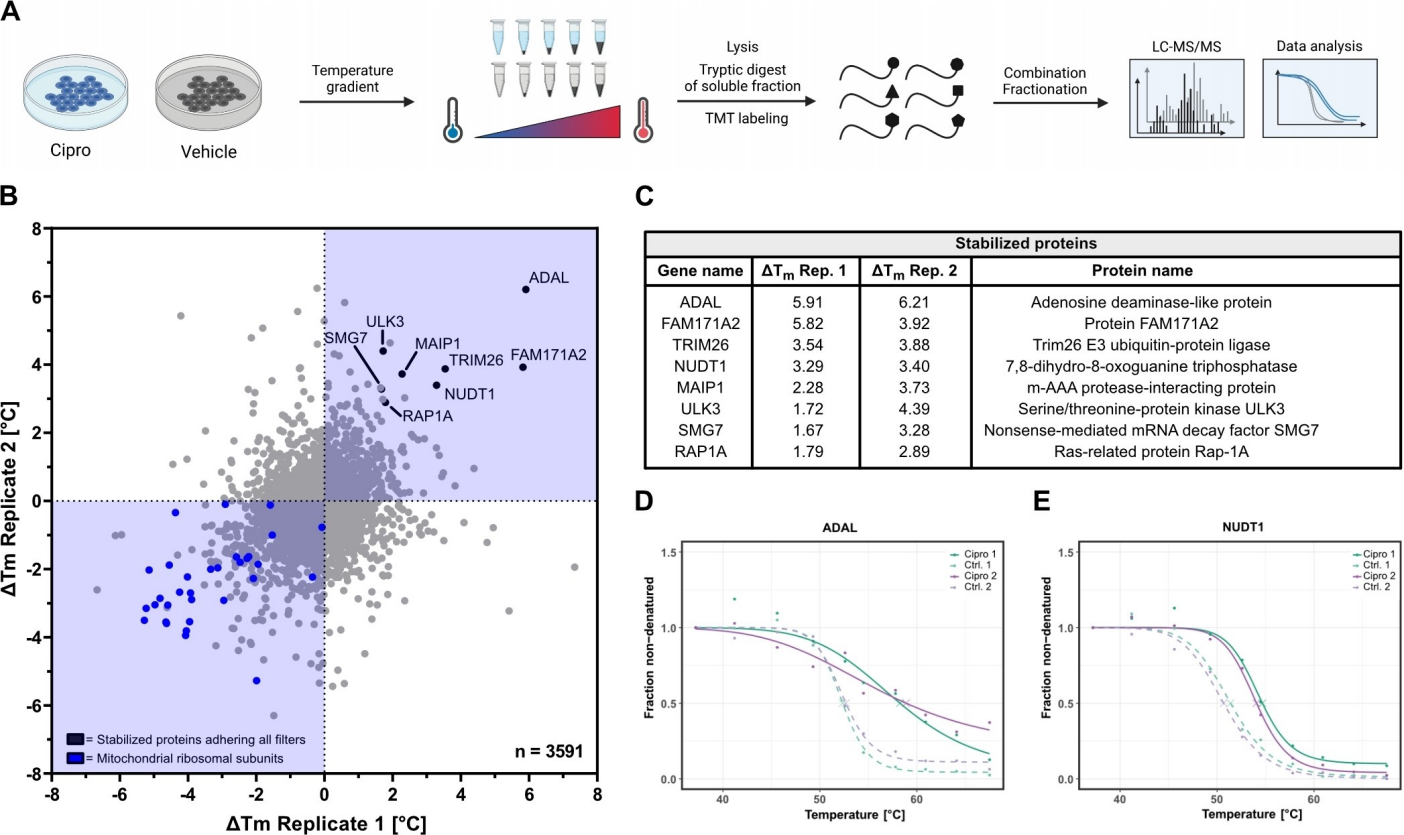
Thermal protein profiling (TPP) of HEK293 cells with Ciprofloxacin. (**A**) General TPP workflow. Cells were treated either with vehicle control or Ciprofloxacin (70 μM) in duplicates for 1 h and cell aliquots were subjected to a temperature gradient with 10 increments. After lysis and ultra‐centrifugation to remove aggregated proteins, the soluble fractions were digested by trypsin and the resulting peptides isotopically labelled with TMT 10‐plex with one distinct channel per temperature. After combination of all 10 TMT channels per sample and offline HILIC fractionation, the samples were measured by LC–MS/MS and the raw data analyzed to infer protein melting curves. (**B**) Scatter plot of the melting point differences of both Ciprofloxacin‐treated replicates in relation to their vehicle‐treated replicates. Stabilized proteins adhering to all filters are labelled in black. Mito‐ribosomal subunits are depicted in blue and were found to be strongly destabilized. (**C**) Table of stabilized proteins adhering to all filters. (**D**) Inferred melting curve of the most strongly stabilized protein ADAL. (**E**) Inferred melting curve of the protein NUDT1 that was found stabilized in the TPP experiment and also has been enriched in the A*f*BPP experiments.

Thermal protein profiling (TPP) is based on protein stabilization (or destabilization) upon small molecule binding. Treated and untreated proteomes are heated to several temperatures and the resulting fraction of soluble proteins is quantified by LC–MS/MS resulting in characteristic protein melting curves (Figure 3A).^[31]^ This method bears the advantage that the unmodified FQ can be directly applied without any derivatization, however, the detection of intrinsically stabilized proteins (e.g. in the membrane or protein dimers) is limited. When performing TPP *in situ* using intact cells rather than lysate, the method is also suitable to detect changes in protein stabilization due to down‐stream and secondary effects on whole pathways. Notably, TPP with Ciprofloxacin has been performed before in bacteria by *Mateus et al*., highlighting the feasibility of this method for FQs.^[68]^ We thus used this method as a complementary approach to A*f*BPP and our whole proteome studies providing additional insights into general proteome stability upon Ciprofloxacin treatment. HEK‐293 cells were treated with Ciprofloxacin (75 μM) for 1 h at 37 °C, processed and analyzed *via* LC–MS/MS. Interestingly even after only 1 hour of treatment, numerous mitochondrial ribosomal subunits were destabilized indicating impaired mitochondrial translation and, again, mitochondrial dysfunction (Figure 3B, S18). *Vice versa*, we obtained few proteins with increased stability upon Ciprofloxacin treatment, including NUDT1 and ADAL (Figure 3B–E). Interestingly, NUDT1 is the single overlapping protein found in both A*f*BPP and TPP experiments. The limited overlap between A*f*BPP and TPP could be attributed to the discussed complementary design of both approaches and highlights the value of performing both methods.

With both protein enrichment (A*f*BPP) and protein stabilization (TPP) data in hand we prioritized putative targets for validation studies. Based on the results we decided to focus on ClpP (mitochondrial proteostasis),[^69^, ^70^] AIFM1 (respiratory chain complexes I and IV biogenesis),[^35^, ^71^] IDH2 (mitochondrial ROS homeostasis, TCA cycle and mitochondrial metabolism and connection to MMP activity),[^72^, ^73^] SCARB1 (cholesterol homeostasis),^[74]^ PTGR2 (inflammation and lipid metabolism),^[75]^ NUDT1 (oxidized purine nucleoside salvage)[^76^, ^77^] and ADAL1 (methylated purine nucleoside salvage).[^78^, ^79^, ^80^] In addition to the discussed photome hits, we decided to exclude general transporter‐like proteins (e.g. for xeniobiotics) and proteins frequently found enriched with the minimalist photo‐crosslinker appended to diverse scaffolds from target validation, as they are likely transiently labeled by the photo‐crosslinker (SCCPDH, SLC25A20, SLC19A1, ABCC4).[^81^, ^82^, ^83^, ^84^, ^85^, ^86^, ^87^, ^88^, ^89^] We excluded NENF, as the protein is secreted and reported to be frequently overexpressed in cancer and immortalized cell lines.^[90]^ Notably, those proteins could, however, give an idea of how the probes and potentially also the parent FQs are translocated into the cells and organelles.

### Effect of Ciprofloxacin and Levofloxacin on Non‐Mitochondrial Targets

The identified hits can be grouped into mitochondrial and non‐mitochondrial localization, which both could contribute to the observed side effects and were thus investigated subsequently. Non‐mitochondrial proteins comprise PTGR2, SCARB1, NUDT1 and ADAL1, which were either cloned and recombinantly expressed or obtained from commercial sources. The activity of PTGR2, a prostaglandin reductase involved in adipogenesis and lipid metabolism,^[75]^ was not significantly affected by Ciprofloxacin (tested up to 250 μM, data not shown) and for SCARB1 that is involved in reverse cholesterol transport^[74]^ weak binding was detected for **Cipro P1**, but no binding affinity could be determined for the parent compound Ciprofloxacin *via* microscale thermophoresis (MST, Figure S19) due to aggregation/precipitation at high compound concentrations excluding both as specific targets.

**Figure 4:**
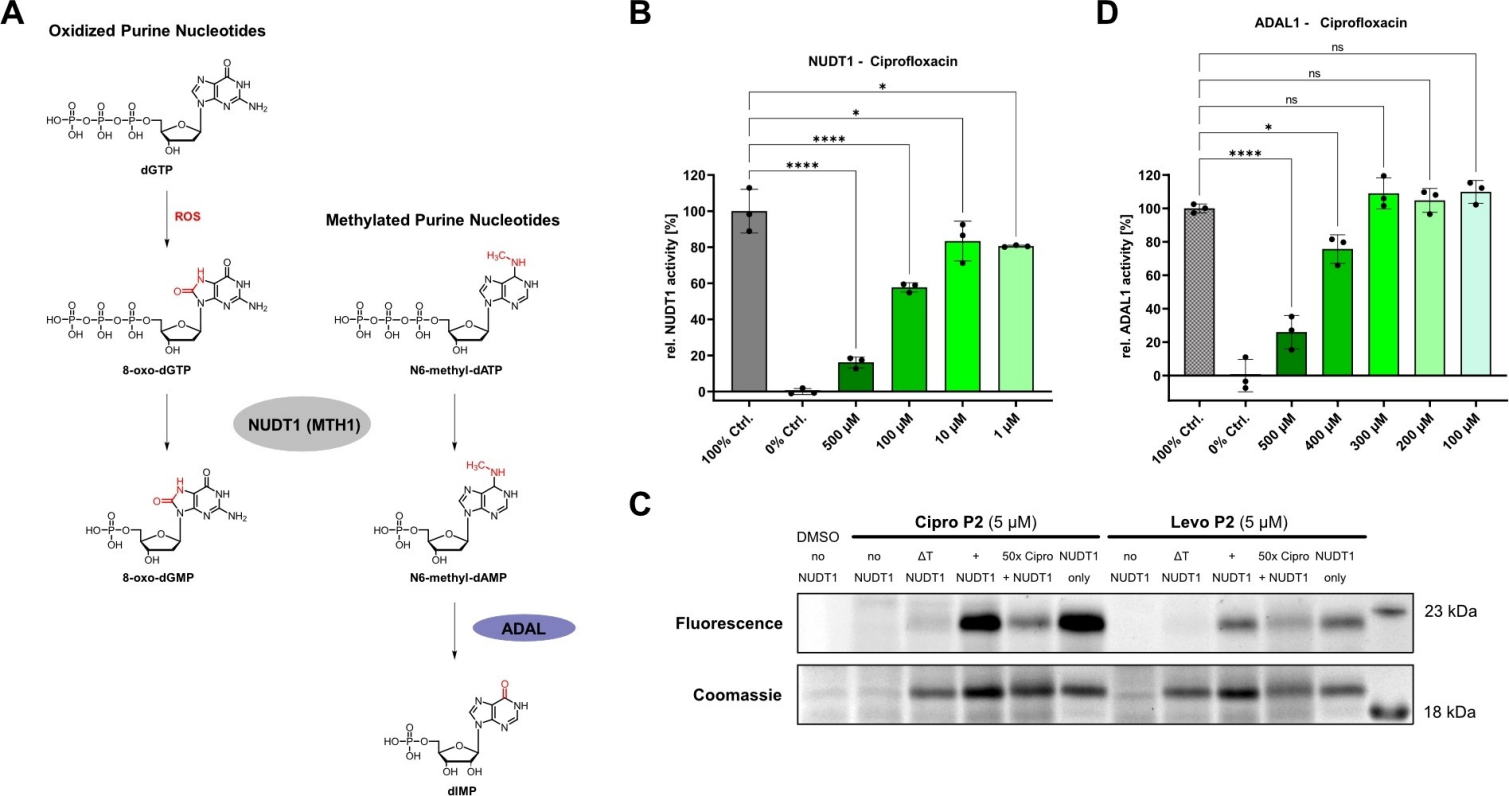
Target validation studies for NUDT1 and ADAL1. (**A**) Activity of NUDT1 purine nucleoside triphosphate hydrolase converting reactive oxygen species (ROS)‐damaged oxidized purine nucleotides to their corresponding monophosphates as exemplary depicted for 8‐oxo‐dGTP. Also N^6^‐methyl‐(d)ATP nucleotides are substrates of NUDT1 resulting in N^6^‐methyl‐(d)AMP, that is substrate of the adenosine deaminase‐like protein ADAL1 converting the methylated nucleoside monophosphate to (d)IMP. (**B**) NUDT1 in vitro activity assay in presence of Ciprofloxacin. The enzymatic activity is partially, but significantly and concentration‐dependently inhibited by physiologically relevant concentrations of Ciprofloxacin. The mean and SD of 3 replicates per condition is plotted. The graph is representative of three independent experiments. (**C**) Gel‐based A*f*BPP of recombinant NUDT1 spiked into a human cell lysate background by **Cipro P2** and **Levo P2**. The labelling is abolished in the heat denatured NUDT1 control (ΔT) and partially outcompeted by the parent FQ further validating NUDT1 engagement by the two FQs. The corresponding Coomassie‐stain is depicted as loading control. The complete gel is depicted in **Figure S20C**. (**D**) In vitro activity assay of ADAL1 in presence of Ciprofloxacin showing significant inhibition only at concentrations >300 μM, which is likely not relevant in physiological conditions. The mean and SD of 3 replicates per condition is plotted. The graph is representative of two independent experiments. In the bar plots the statistical relevance based on one‐way ANOVA with Dunnett's multiple comparison test is depicted (ns meaning P‐value >0.05, * P‐value≤0.05, ** P‐value≤0.01, *** P‐value≤0.001, **** P‐value≤0.0001).

The enzyme NUDT1 (also called MTH1) hydrolyses oxidized purine nucleoside triphosphates (2‐oxo‐ATP, 2‐oxo‐dATP, 8‐oxo‐GTP, 8‐oxo‐dGTP) to their respective monophosphates sanitizing the NTP pool of ROS‐damaged DNA and RNA precursors and thereby, protecting cells from missense base‐paring (Figure 4A).[^76^, ^77^] NUDT1 is non‐essential in healthy cells, however crucial under high oxidative stress, e.g. in cancer cells.^[91]^ In fact, various NUDT1 inhibitors have been developed eradicating cancer by “cancer phenotypic lethality”, including quinoline and quinolinedione compounds sharing structural similarities with FQs.[^91^, ^92^, ^93^] As also FQs increase ROS, a potential NUDT1 activity modulation by FQs is likely to contribute to their human side‐effects.[^4^, ^12^, ^15^] Indeed, incubation with Ciprofloxacin affected NUDT1 in vitro activity in a dose‐dependent manner with significant partial inhibition at a high but physiologically relevant concentration of 100 μM (Figure 4B). Inhibition of NUDT1 by Levofloxacin was only significant at the highest concentration tested. The known specific inhibitor (*S*)‐crizotinib served as positive control (Figure S20A–B).^[93]^ Additionally, gel‐based labeling of recombinant NUDT1 spiked into human lysate as background by **Cipro P2** and **Levo P2** was abolished for temperature‐denatured NUDT1 and competed by parent Ciprofloxacin (Figure 4C, **S20C**), further corroborating NUDT1 as an off‐target of FQs. Of note, the inhibition of NUDT1 is in line with elevated 8‐oxo‐dG levels, which have been observed in cells treated with Ciprofloxacin and could thus contribute to its long‐lasting side‐effects.^[12]^

Adenosine deaminase‐like protein ADAL1, the most stabilized protein in TPP, operates downstream of NUDT1 by converting N^6^‐methyl dAMP/AMP to dIMP/IMP in the purine salvage pathway (Figure 4A).[^78^, ^79^, ^80^] Activity assays in presence of Ciprofloxacin showed partial enzyme inhibition only at very high concentrations≥300 μM (Figure 4D, S21A–B). Although this is likely not relevant for the side effects of FQs, it could explain the stabilization observed with TPP.^[79]^ For Levofloxacin even at high concentrations no inhibitory effect was detected (Figure S21C).

### Studies into Mitochondrial Off‐Targets Validate AIFM1 and IDH2 as Drivers for FQ Dysregulation of Cells

In light of our whole proteome analysis data showing significant downregulation of both the electron transport chain, especially complexes I and IV, and the destabilized mito‐ribosomal subunits in the TPP experiment, we next focused our target validation efforts on mitochondrial protein hits. We started with ClpP, the proteolytic subunit of the mitochondrial caseinolytic protease P involved in the maintenance, maturation and quality control of various mitochondrial proteins, including the ETC and the mito‐ribosome.^[94]^ We recombinantly expressed and purified ClpP and tested its activity against the compounds in ClpP peptidase and ClpXP protease assays. Unexpectedly, while literature‐known positive controls for enzyme inhibition (TG42) and overactivation (ONC201) resulted in clear effects, Ciprofloxacin evoked no significant concentration‐dependent activation or inhibition (Figure S22C–D).[^95^, ^96^] In line with this observation, a full proteome analysis of HEK‐293 wild‐type and ClpP knockout cells in presence of Ciprofloxacin revealed almost identical changes within up‐ and downregulated proteins (Ciprofloxacin vs. vehicle control) suggesting that a potential interaction between Ciprofloxacin and ClpP does not play a major role for proteome dysregulation and thereby for toxicity (Figure S22E–F).^[70]^

**Figure 5:**
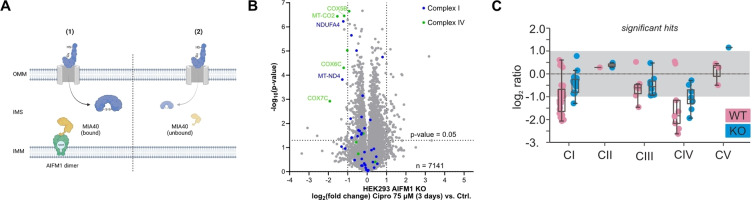
Effect of FQs on AIFM1. (**A**) Schematic overview of AIFM1 and MIA40 interaction. (**1**) NADH‐dependent dimerization of AIFM1 enables binding and import of MIA40 forming a stable active trimer. MIA40 mediates the import of specific nuclear‐encoded proteins into the intermembrane space (IMS) as key component of the mitochondrial disulfide relay system and further facilitates the oxidative folding of imported substrates. Various MIA40‐AIFM1 substrates are structural components of ETC complex I and IV or are involved in their biogenesis as assembly factors. (**2**) AIFM1 knockout or perturbation of the AIFM1‐MIA40 interaction results in reduced MIA40 and consequently substrate protein concentrations in the IMS. Figure based on *Salscheider et al*.^[35]^ AIFM1 knockout cells were generated using CRISPR/Cas9, and individual clones were verified by sequencing, immunoblotting and proteomics. (**B**) Volcano plot depicting the proteome regulation of AIFM1 knockout HEK293 cells treated with Ciprofloxacin (75 μM) for 3 days versus vehicle control‐treated AIFM1 knockout cells featuring only minor additional alterations of ETC subunits. Threshold lines represent a log_2_ regulation of ±1 and – log_10_ (*P*‐value) of 1.3 (two‐sided two‐sample *t*‐test, *n*=4 replicates per group). The volcano plot of the wild‐type (WT) control experiment is depicted in Figure S23A. (**C**) Boxplot showing the comparison of respiratory chain complex protein levels in HEK293 WT or AIFM1 KO cells treated with ciprofloxacin or left untreated. Changes of protein levels of all significantly affected subunits of the respective respiratory chain complexes upon ciprofloxacin treatment were plotted. In WT cells, levels of complex I and IV subunits decrease upon ciprofloxacin treatment. This effect is attenuated in AIFM1 KO cells indicating that ciprofloxacin acts on AIFM1.

Next, in‐depth validation studies for apoptosis‐inducing factor 1 (AIFM1) were performed. AIFM1 is a mitochondrial intermembrane space (IMS) protein anchored to the inner mitochondrial membrane.^[97]^ Among its function in non‐caspase‐mediated cell death, AIFM1 is crucially involved in the IMS import machinery. AIFM1 facilitates the binding and translocation of MIA40 (also called CHCHD4), the key component of the mitochondrial disulfide relay system, into the IMS.[^35^, ^71^] Substrates of this redox‐regulated system are small nuclear encoded cysteine‐containing proteins important for mitochondrial processes, such as ETC complex biogenesis, mitochondrial translation and lipid homeostasis, that are imported into the IMS and oxidatively folded by MIA40.^[98]^ Partial loss of AIFM1, such as in hypomorphic harlequin (Hq) mice, therefore results in severe mitochondrial dysfunction including complex I and IV dysfunction.^[99]^ In accordance, whole proteome analyses of HEK293 cells bearing a complete AIFM1 knockout (KO) generated by CRISPR‐Cas9 technology revealed a strikingly similar proteotype to FQ‐treated cells with a strong complex I and IV down‐regulation.^[35]^ In order to study the effects of FQ on AIFM1 in cells directly, we compared proteome changes of Ciprofloxacin‐treated and untreated HEK‐293 wt with treated and untreated KO cells (Figure 5B–C, **S23**). In accordance with the literature, we see overall similar pattern of protein regulation of treated wt cells, in particular confirming downregulation of complex I and IV. Strikingly, proteomic comparison between Ciprofloxacin treated and untreated KO cells show only little additional alterations highlighting that Ciprofloxacin needs the presence of AIFM1 to affect respiratory chain complex levels (Figure 5B–C, S23).

To investigate a direct interaction between the compound and the protein, we expressed and purified recombinant AIFM1. The protein was spiked in HEK‐293 lysate followed by addition of 5 μM **Cipro P1**, click to rhodamine azide and analysis *via* fluorescent SDS‐PAGE analysis. A strong fluorescent band corresponding to the molecular weight of AIFM1 was visible in the spiked sample, which was absent in the control lacking the protein (Figure S23E). Moreover, addition of excess Ciprofloxacin prior to probe addition resulted in a strong reduction of signal intensity, confirming that Ciprofloxacin binds AIFM1 and addresses the same binding site as the probe. Consequently, our results indicate that Ciprofloxacin binds AIFM1, thereby inducing mitochondrial dysfunction and ETC complex I and IV downregulation.

**Figure 6:**
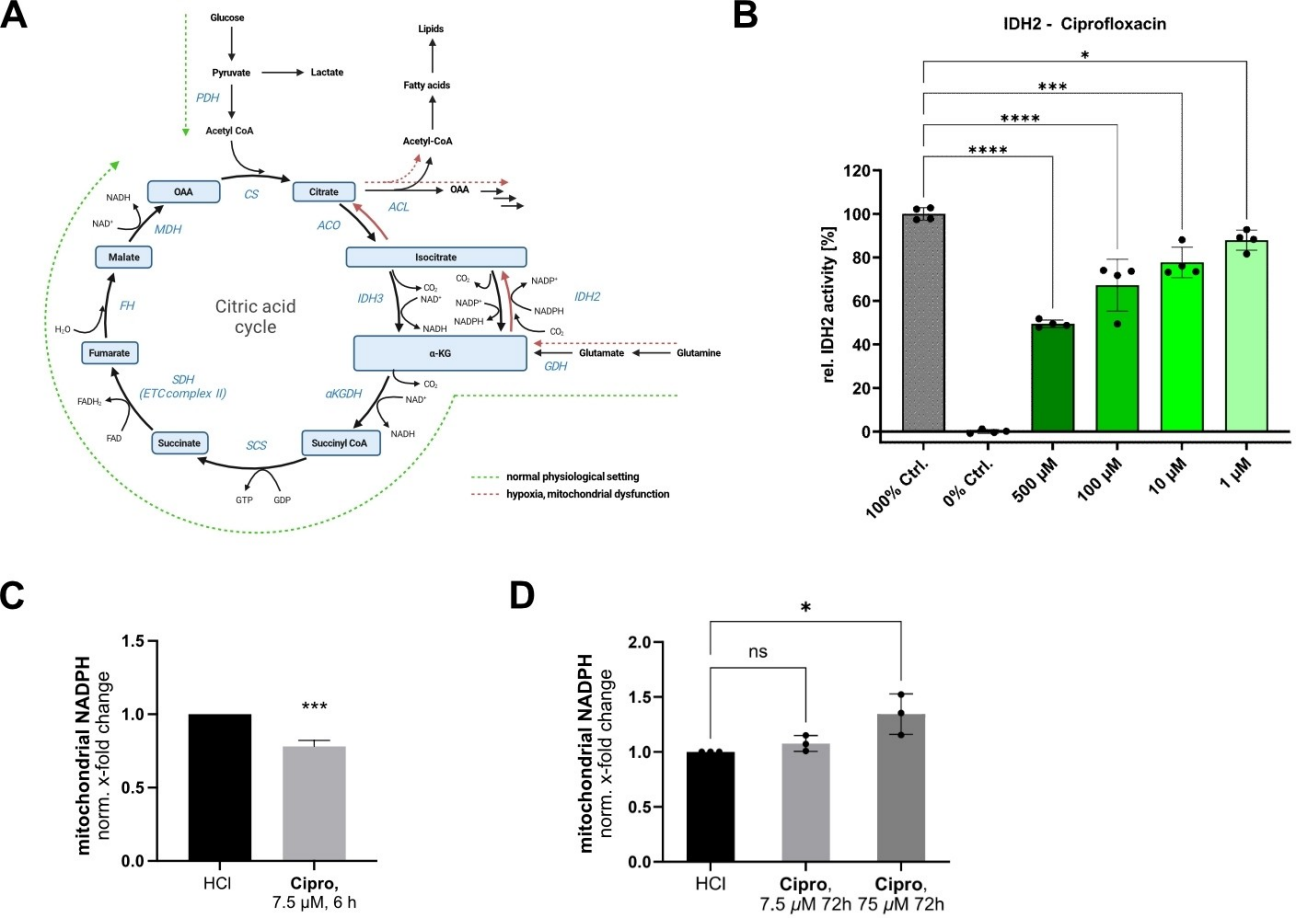
Effect of FQs on the mitochondrial isocitrate dehydrogenase IDH2. (**A**) IDH2 catalyzes the conversion of isocitrate and α‐ketoglutarate (αKG) bidirectionally and is therefore important for metabolic rewiring in hypoxic conditions and in case of mitochondrial dysfunction when reductive glutaminolysis (red pathway) is the prominent carbon source for citrate formation. (**B**) IDH2 in vitro activity assay in presence of Ciprofloxacin. Partial, but concentration‐dependent inhibition in a physiologically relevant range was detected. The mean and SD of 4 replicates per condition is plotted. The graph is representative of three independent experiments. (**C**) Mitochondrial NADPH levels are decreased by approx. 25 % after incubation of PDL‐hTERT cells with Ciprofloxacin (7.5 μM, 6 h) in line with the in vitro inhibition of the forward oxidative reaction of IDH2. To prevent the onset of ETC dysregulation in line with our proteomic data (compare Figure 1, S1,2,4) the lower concentration was deliberately chosen for this time point (average and mean of 3 independent experiments plotted, unpaired two‐tailed *t*‐test, *P*‐value <0.001). (**D**) Prolonged incubation, especially with the higher Ciprofloxacin concentration (75 μM, 3 days) resulted in elevated mitochondrial NADPH levels in PDL‐hTERT cells, potentially as a result of the inhibition of the reverse reductive carboxylation reaction of IDH2 that is reported to be prominent in settings of mitochondrial dysfunction (average and mean of 3 independent experiments plotted).^[102]^ In the bar plots in **B**, **C** and **D** the statistical relevance based on one‐way ANOVA with Dunnett's multiple comparison test is depicted (ns meaning P‐value >0.05, * P‐value≤0.05, ** P‐value≤0.01, *** P‐value≤0.001, **** P‐value≤0.0001).

The isocitrate dehydrogenase IDH2 interconverting isocitrate and α‐ketoglutarate (αKG) is involved in various essential pathways, such as the production of mitochondrial NADPH, a crucial cofactor, e.g. for iron‐sulfur cluster biogenesis, cholesterol biosynthesis and the regeneration of the glutathione reactive‐oxygen (ROS) scavenging system.[^72^, ^73^, ^100^] Additionally, IDH2 downregulation has been associated with increased matrix metalloproteinase (MMP) activity *via* increased NF‐kB signaling.[^72^, ^73^] Importantly, hypoxic conditions as well as mitochondrial ETC dysfunction trigger the reverse reductive carboxylation of αKG to isocitrate under NADPH consumption (Figure 6A).[^101^, ^102^]

Interestingly, the *in vitro* forward oxidative decarboxylation activity of isocitrate to αKG of IDH2 was significantly inhibited upon Ciprofloxacin and Levofloxacin treatment by 25–30 % reduction at 100 μM (Figure 6B, Figure S24A). The extent of inhibition was similar in presence of Enadisenib, a known inhibitor for IDH2 with point mutations involved in various cancers, that exhibits a weaker potency for the wild‐type enzyme (Figure S24B).^[103]^

To check if impaired IDH2 activity correlates with altered mitochondrial NADPH synthesis we determined its levels after short and long Ciprofloxacin treatments. As expected, exposure for 6 h in PDL‐hTERT cells revealed a NADPH decrease of approximately 25 % at low ciprofloxacin concentrations (only 7.5 μM tested to prevent the early onset of ETC dysregulation at high concentrations), which is in line with inhibition of IDH2's forward oxidative decarboxylation activity. However, prolonged incubation for 3 days especially at high Ciprofloxacin concentration (75 μM) induced the above described ETC dysfunction (as determined by FQ whole proteome experiments) and thus, resulted in an increase of NADPH levels likely based on IDH2's metabolic rewiring and switching to the reverse reductive carboxylation reaction (Figure 6C–D).^[102]^ Thus, the FQ induced ETC dysfunction *via* AIFM1 and the impaired formation of isocitrate in the reverse carboxylation pathway of IDH2 simultaneously contribute to mitochondrial dysfunction.

## Conclusion

Selective toxicity against prokaryotic over eukaryotic targets is the fundamental principle of safe antibacterial agents. In praxis, total selectivity is hard to achieve and, in fact, for many commonly used antibiotics side‐effects on eukaryotic targets are known.[^12^, ^104^] For FQs, although not generally occurring, adverse events can be especially devastating and long‐lasting. Here, we applied (chemo‐)proteomic approaches to decipher molecular mechanisms of FQ‐derived human side‐effects. Whole proteome analyses revealed significant protein dysregulation already after 3 h of treatment and pinpointed the reported mitochondrial toxicities of FQs to an arising proteotype with selective down‐regulation of the complexes I and IV of the ETC in dependence of the FQ concentration. Subsequently, A*f*BPP and TPP were employed to identify protein off‐targets.

**Figure 7:**
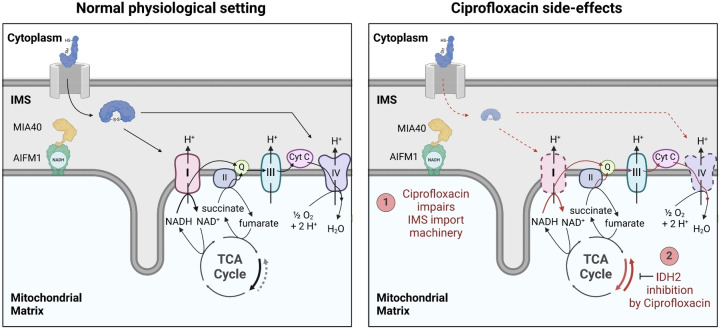
Schematic overview of identified Ciprofloxacin adverse effects regarding mitochondrial toxicity. AIFM1 is involved in the MIA40‐mediated IMS import and oxidative folding of specific nuclear encoded proteins including subunits of the ETC complex I and IV (compare Figure 5A). Ciprofloxacin impairs the IMS import machinery leading to ETC impairment. Dysfunction of the ETC, especially of complex I results in a decrease of the NAD^+^/NADH ratio. The change in redox‐homeostasis and shortage of NAD^+^, a key cofactor for the TCA cycle, can lead to the metabolic rewiring involving IDH2 as discussed in Figure 6A. Importantly, IDH2 function was also shown to be impaired by Ciprofloxacin, therefore also this compensation mechanism is impaired by FQs.

The most prominent side effect of FQ treatment, the specific down‐regulation of the ETC complexes I and IV, could be linked to an interaction of FQs with AIFM1 and its functional impairment comparable to a cellular KO. However, insights on how FQ binding to AIFM1 induces the dysregulation is subject to future investigations, i.e. requiring insights from structural biology. As a consequence of this interaction, the corresponding damage in the respiratory chain causes a reduction in NAD+ levels, i.e. an accumulation of NADH, leading to metabolic rewiring and a switch in the catalysis of IDH2 from a‐KG to isocitrate production. As simultaneous inhibition of IDH2 by FQs also inactivates this alternative reductive carboxylation route, mitochondria are incapable of compensating the shortage of crucial metabolites of the TCA cycle likely triggering a pronounced dysfunction (Figure 7). Of note, mitochondrial NADH/NAD+ and NADPH/NADP+ pools are linked by the nicotinamide nucleotide trans‐hydrogenase (NNT), thus an accumulation of NADH due to mitochondrial dysfunction could contribute to increased NADPH levels.^[105]^

Additionally, non‐mitochondrial protein interactions with NUDT1, reported divalent ion chelation and other modes of action likely contribute to the side‐effects. Besides the better understanding of molecular off‐targets of FQs, it is important to study why only some patients are so severely affected by adverse reactions.^[106]^ While risk factors like concomitant drug prescriptions and changed pharmacokinetics, e.g. due to renal impairment or age, leading to drug accumulation can partially explain the sensibility of some patients towards FQs, further studies are needed. To the best of our knowledge no studies into genetic predispositions of effected patients have been published, although insinuations about pending patent applications in this field have been made.[^5^, ^107^, ^108^] The overlay of genomic data with the off‐targets identified and validated in this study could potentially support and direct the development of safe FQ prescription practices and newer FQ generations.

## Methods

Detailed experimental procedures and methods are provided in the Supporting Information. Figures were created with *biorender.com* and affinity designer version 1.9.2.1035.

## Author Contributions

T.R. planned the project and performed chemical synthesis, proteomics experiments, protein expression and purification, bioactivity assays, data analysis and data visualization. N.B. performed proteomics experiments and planned the project. Y.E.H. conducted chemical synthesis, performed proteomics experiments and data analysis. A.T.J. performed lysosomal cell imaging and analyzed mitochondrial NADPH levels. F.T. performed pathway analyses, conducted FACS and hmdC level measurements. A.R. conducted AIFM1 target validation studies. M.K. conducted chemical synthesis. T.Ri. performed SCARB1 MST measurements. L.H.C.J. performed bioinformatics resulting in the boxplot analyses. S.M‐D. and D.D. provided PDL‐hTERT cells, live/dead‐assay and qPCR experiments. J.R. planned AIFM1 target validation. S.Z. planned cell imaging and NADPH level analyses. S.A.S conceived, planned and supervised the project. All authors reviewed and approved the manuscript.

## Conflict of Interests

The authors declare no competing interests.

1

## Supporting information

As a service to our authors and readers, this journal provides supporting information supplied by the authors. Such materials are peer reviewed and may be re‐organized for online delivery, but are not copy‐edited or typeset. Technical support issues arising from supporting information (other than missing files) should be addressed to the authors.

Supporting Information

## Data Availability

The mass spectrometry proteomics data have been deposited to the ProteomeXchange Consortium *via* the PRIDE partner repository with the data set identifier PXD047056 and PXD056813.^[109]^
