# Supplementary material for: Chemical Proteomics Reveals Human Off‐Targets of Fluoroquinolone Induced Mitochondrial Toxicity
Source: Angew Chem Int Ed Engl. 2025 Apr 2;64(18):e202421424. doi: 10.1002/anie.202421424 (PMC12036814; doi:10.1002/anie.202421424)
Supplement: Supplementary file 1 — Supporting Information [file ANIE-64-e202421424-s001.pdf]

Angewandte Chemie
Eine Zeitschrift der Gesellschaft Deutscher Chemiker

Supporting Information

Chemical Proteomics Reveals Human Off-Targets of Fluoroquinolone Induced Mitochondrial Toxicity

T. Reinhardt, Y. El Harraoui, A. Rothemann, A. T. Jauch, S. Müller-Deubert, M. F. Köllen, T. Risch, L. J. Jacobs, R. Müller, F. R. Traube, D. Docheva, S. Zahler, J. Riemer, N. C. Bach, S. A. Sieber**

Supplementary Figures

Chemical proteomics reveal human off-targets of fluoroquinolone induced mitochondrial toxicity

Till Reinhardt[1], Yassmine El Harraoui[1], Alex Rothemann[2], Adrian T. Jauch[3], Sigrid Müller-Deubert[4], Martin F. Köllen[1], Timo Risch[5], Lianne H.C. Jacobs[2], Rolf Müller[5], Franziska R. Traube[6], Denitsa Docheva[4], Stefan Zahler[3], Jan Riemer[2], Nina C. Bach[1,*] and Stephan A. Sieber[1,*]

[1]Center for Functional Protein Assemblies, Department of Bioscience, TUM School of Natural Sciences, Technical University of Munich, Ernst-Otto-Fischer-Straße 8, 85748 Garching, Germany.

[2]Institute for Biochemistry and CECAD, University of Cologne, Cologne, Germany.

[3]Department of Pharmacy, Pharmaceutical Biology, Ludwig-Maximilians-Universität München, Butenandtstraße 5-13, 81377 Munich, Germany.

[4]Department of Musculoskeletal Tissue Regeneration, Orthopaedic Hospital König-Ludwig-Haus, University of Würzburg, 97076 Würzburg, Germany.

[5]Helmholtz Institute for Pharmaceutical Research Saarland (HIPS), Helmholtz Centre for Infection Research (HZI) and Saarland University Department of Pharmacy, Campus Building E8.1, 66123 Saarbrücken, Germany.

[6] Institut für Biochemie und Technische Biochemie, Universität Stuttgart, 70569 Stuttgart.

[*]Corresponding author(s).

Figure S1

A

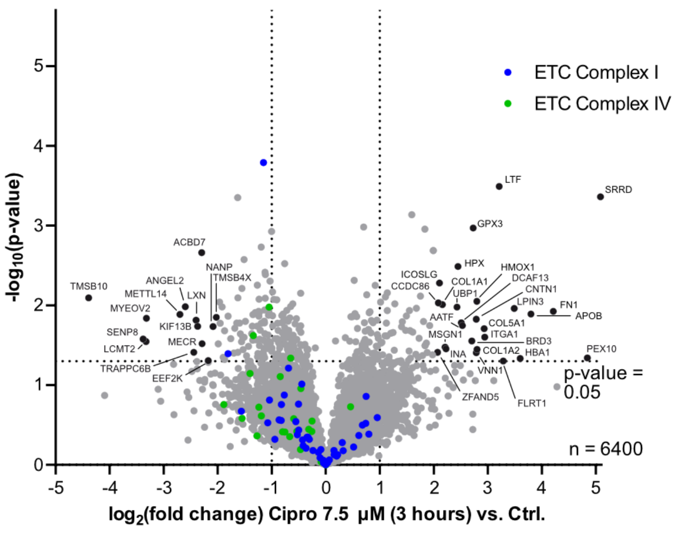

B

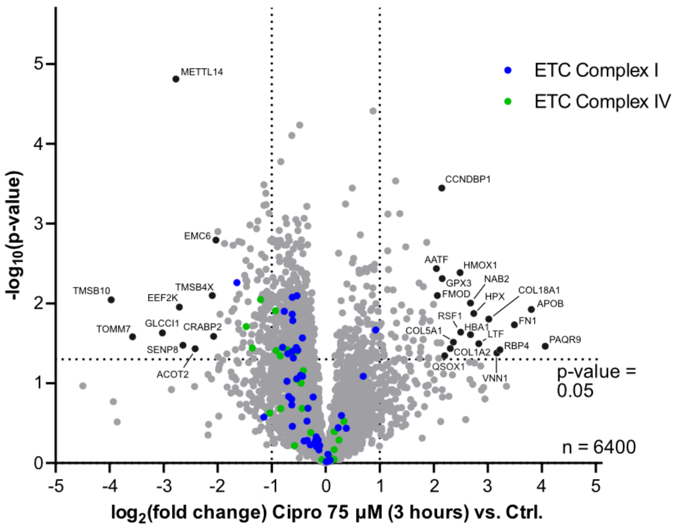

C

Up-regulated proteins (fold-change ≥ log₂ = + 2)

	Gene	Protein name
	AATF	Protein AATF
*	APOB	Apolipoprotein B-100
	BRD3	Bromodomain-containing protein 3
	CCDC86	Coiled-coil domain-containing protein 86
	CCNDBP1	Cyclin-D1-binding protein 1
	CNTN1	Contactin-1
*	COL18A1	Collagen alpha-1(XVIII) chain
*	COL1A1	Collagen alpha-1(I) chain
*	COL1A2	Collagen alpha-2(I) chain
*	COL1A5	Collagen alpha-1(V) chain
	DCAF13	DDB1- and CUL4-associated factor 13
	FLRT1	Leucine-rich repeat transmembr. prot. FLRT1
	FMOD	Fibromodulin
*	FN1	Fibronectin
	GPX3	Glutathione peroxidase 3
	HBA1	Hemoglobin subunit alpha
*	HMOX1	Heme oxygenase 1
	HPX	Hemopexin
*	ICOSLG	ICOS ligand
	INA	Alpha-internexin
	ITGA1	Integrin alpha-1
	LPIN3	Phosphatidate phosphatase LPIN3
*	LTF	Lactotransferrin
	MSGN1	Mesogenin-1
	MST1	Hepatocyte growth factor-like protein
	NAB2	NGFI-A-binding protein 2
	PAQR9	Progestin and adipoQ receptor family member 9
	PEX10	Peroxisome biogenesis factor 10
	QSOX1	Sulfhydryl oxidase 1
	RBP4	Retinol-binding protein 4
*	RSF1	Remodeling and spacing factor 1
	SRRD	SRR1-like protein
	UBP1	Upstream-binding protein 1
	UTP18	U3 small nucleolar RNA-associated protein 18 homolog
	VNN1	Pantetheinase
	WIPF1	WAS/WASL-interacting protein family member 1
	ZFAND5	AN1-type zinc finger protein 5

* = Involved in NF-κB signaling

D

Down-regulated proteins (fold-change ≤ log₂ = −2)

Gene	Protein name
ACBD7	Acyl-CoA-binding domain-containing protein 7
ACOT2	Acyl-coenzyme A thioesterase 2, mitochondrial
ANGEL2	Protein angel homolog 2
CRABP2	Cellular retinoic acid-binding protein 2
EEF2K	Eukaryotic elongation factor 2 kinase
EMC6	ER membrane protein complex subunit 6
GLCCI1	Glucocorticoid-induced transcript 1 protein
KIF13B	Kinesin-like protein KIF13B
LCMT2	tRNA wybutosine-synthesizing protein 4
LXN	Latexin
MECR	Trans-2-enoyl-CoA reductase, mitochondrial
METTL14	N6-adenosine-methyltransferase subunit METTL14
MYEOV2	Myeloma-overexpressed gene 2 protein
NANP	N-acylneuraminate-9-phosphatase
SENP8	Sentrin-specififc protease 8
TMSB10	Thymosin beta-10
TMSB4X	Thymosin beta-4
TOMM7	Mitochondrial import receptor subunit TOM7 homolog
TRAPPC6B	Trafficking protein particle complex subunit 6B

Figure S1: Whole proteome analysis of Ciprofloxacin in HEK-293 cells after 3 hours of incubation. (**A**) Volcano plot depicting the proteome regulation of the low (7.5 µM) concentration of Ciprofloxacin versus the control. (**B**) Volcano plot depicting the proteome regulation of the high (75 µM) concentration versus the control. Threshold lines represent a $\log_2$ regulation of ±1 and – $\log_{10}$ (*P*-value) of 1.3 (two-sided two-sample *t*-test, *n* = 3 replicates per group). Subunits of the electron transport chain (ETC) complexes I and IV are depicted in blue and green, respectively. (**C**) Alphabetically sorted table of top up-regulated proteins at either or both concentrations (threshold: $\log_2$ regulation ≥ 2). Proteins involved in NF-κB signaling are denoted by the XXX.[1–3] (**D**) Alphabetically sorted table of top down-regulated proteins at either or both concentrations (threshold: $\log_2$ regulation ≤ –2).

Figure S2

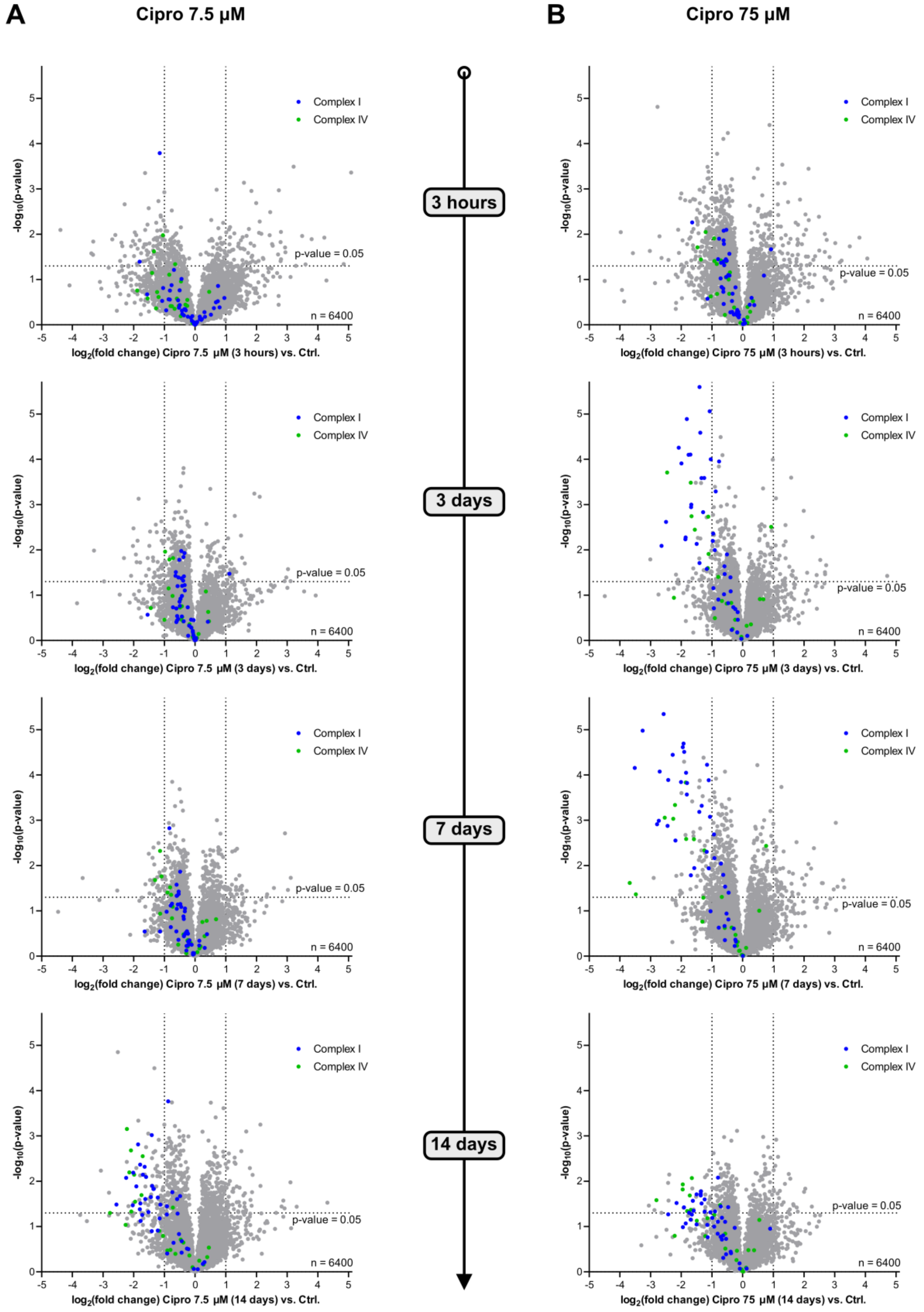

Figure S2: Whole proteome analysis of Ciprofloxacin in HEK-293 cells after 3 h, 3 days, 7 days and 14 days of incubation. (**A**) Respective volcano plots depicting the proteome regulation of the low (7.5 µM) concentration of Ciprofloxacin versus the control. Please note that the volcano plots of Ciprofloxacin in HEK-293 cells after 3 h shown in Figure S1 are depicted here again for a direct comparison. (**B**) Volcano plot depicting the proteome regulation of the high (75 µM) concentration versus the control. Threshold lines represent a $\log_2$ regulation of ±1 and $-\log_{10}$ (*P*-value) of 1.3 (two-sided two-sample *t*-test, $n = 3$ replicates per group). Subunits of the electron transport chain (ETC) complexes I and IV are depicted in blue and green, respectively. A pronounced down-regulation of both complexes is observable in time and concentration dependency.

Figure S3

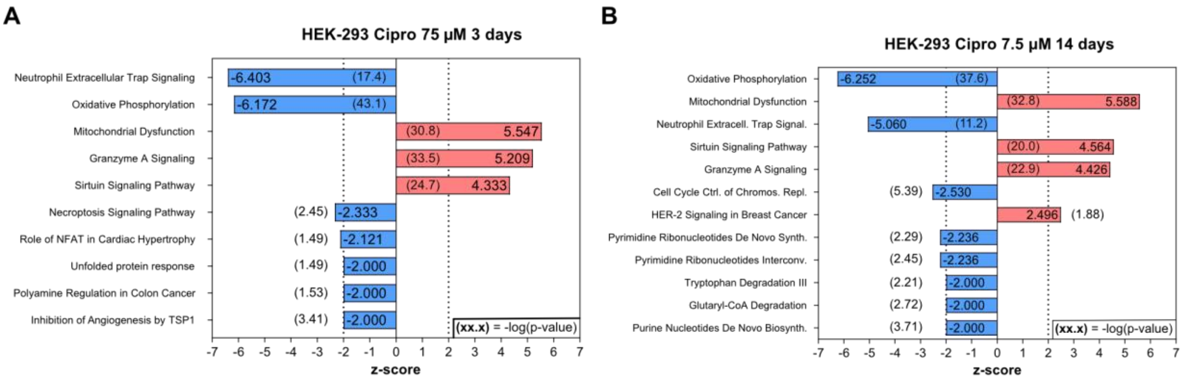

Figure S3: Proteome regulation pathway analyses of Ciprofloxacin-treated HEK-293 cells at high concentration after 3 days (**A**) and low concentration after 14 days (**B**). The identity, extent (z-score, threshold ±2) and statistical significance (– $\log_{10}$ *P*-value, in brackets) of both conditions are comparable. Consequently, higher drug concentrations merely lead to an earlier onset of dysregulation.

Figure S4

A

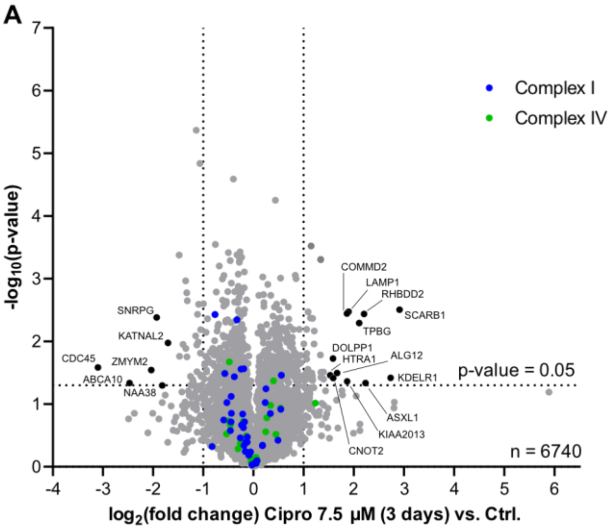

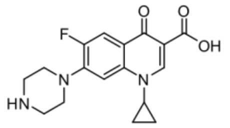

B

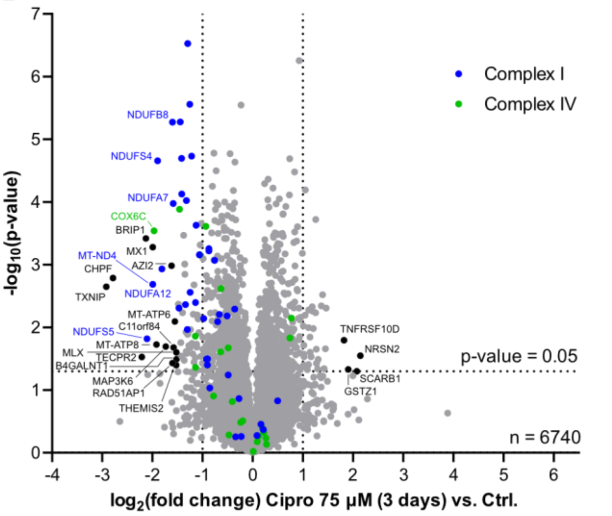

C **Up-regulated proteins (fold-change ≥ log₂ = 1.5)**

Gene	Protein name
ALG12	Mannosyltransferase ALG12 homolog
ASXL1	Polycomb group protein ASXL1
CNOT2	CCR4-NOT transcription complex subunit 2
COMMD2	COMM domain-containing protein 2
DOLPP1	Dolichyldiphosphatase 1
GSTZ1	Maleylacetoacetate isomerase
HTRA1	Serine protease HTRA1
KDELR1	ER lumen protein-retaining receptor 1
KIAA2013	Unchar. protein KIAA2013
LAMP1	Lysosome-associated membrane glycoprotein 1
NRSN2	Neurensin-2
RHBDD2	Rhomboid domain-containing protein 2
SCARB1	Scavenger receptor class B member 1
TNFRSF10D	Tumor necrosis factor receptor superfam. memb. 10D
TPBG	Trophoblast glycoprotein

D **Down-regulated proteins (fold-change ≤ log₂ = −1.5)**

Gene	Protein name
2MYM2	Myomesin-2
ABCA10	ATP-binding casette sub-family A member 10
AZI2	5-azacytidine-induced protein 2
B4GALNT1	Beta-1,4-N-acetylgalactosaminyltransferase 1
BRIP1	Fanconi anemia group J protein
C11orf84	Spindlin interactor and repressor of chromatin-binding prot.
CDC45	Cell division control protein 45 homolog
CHPF	Chondroitin sulfate glucuronyltransferase
COX6C	Cytochrome c oxidase subunit 6C
KATNAL2	Katanin p60 ATPase-contain. subunit A-like 2
MAP3K6	Mitogen-activated protein kinase kinase kinase 6
MLX	Max-like protein X
MT-ATP8	ATP synthase protein 8
MT-ATP6	ATP synthase subunit a
MT-ND4	NADH-ubiquinone oxidoreductase chain 4
MX1	Interferon-induced GTP-binding protein Mx1
NAA38	N-alpha-acetyltransferase 38
NDUFA12	NADH dehydrogenase [ubiquinone] 1 alpha subcomplex subunit 12
NDUFA7	NADH dehydrogenase [ubiquinone] 1 alpha subcomplex subunit 7
NUDFB8	NADH dehydrogenase [ubiquinone] 1 beta subcomplex subunit 8
NDUFS4	NADH dehydrogenase [ubiquinone] iron-sulfur protein 4
NUDFS5	NADH dehydrogenase [ubiquinone] iron-sulfur protein 5
RAD51AP1	RAD51-associated protein 1
SNRPG	Small nuclear ribonucleoprotein G
TECPR2	Tectonin beta-propeller rpeat containing prot. 2
THEMIS2	Protein THEMIS2
TXNIP	Thioredoxin-interacting protein

Figure S4: Whole proteome analysis of Ciprofloxacin in PDL-hTERT cells after 3 days of incubation. (**A**) Volcano plot depicting the proteome regulation of the low concentration versus the control. (**B**) Volcano plot of the high concentration versus the control. Vertical threshold lines represent a log_2 regulation of ±1 and – log_{10} (*P*-value) of 1.3 (two-sided two-sample *t*-test, $n = 4$ replicates per group). Subunits of the ETC complex I (blue) and complex IV (green) are highlighted. (**C**) Top up-regulated proteins in alphabetical order (threshold: fold-change ≥ log_2 1.5). (**D**) Top down-regulated proteins in alphabetical order (threshold: fold-change ≤ log_2 –1.5).

Figure S5

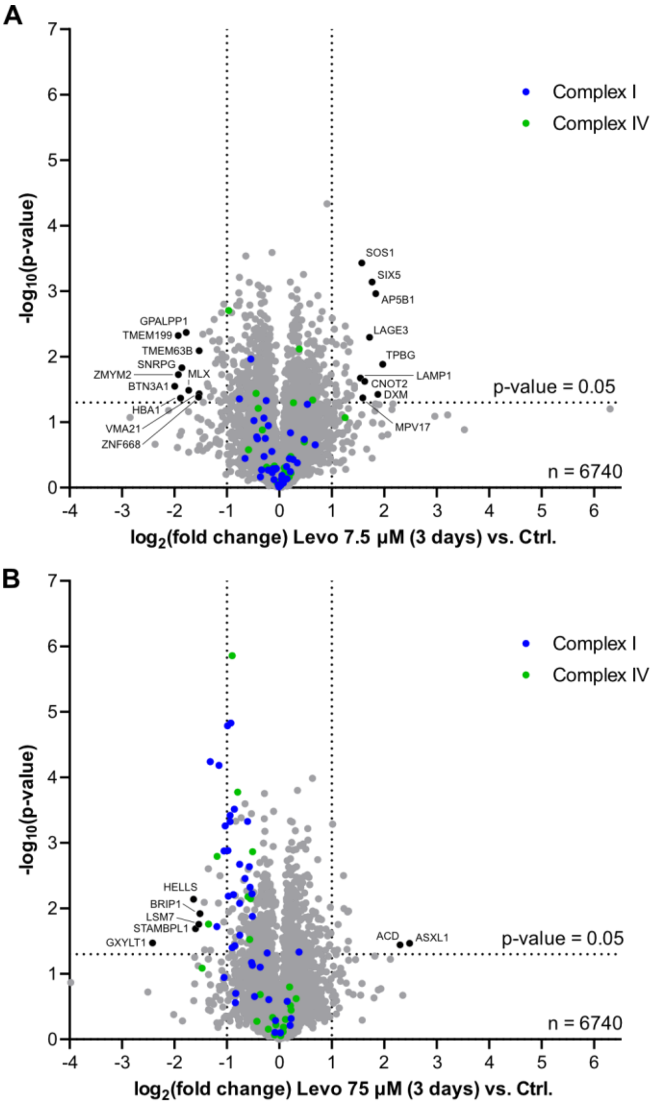

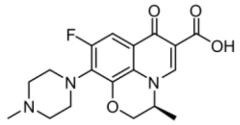

Levofloxacin
Whole proteome analysis in PDL cells, 3 days

C **Up-regulated proteins (fold-change ≥ log₂ = 1.5)**

Gene	Protein name
ACD	Adrenocortical dysplasia protein homolog
AP5B1	AP-5 complex subunit beta-1
ASXL1	Putative Polycomb group protein ASXL1
CNOT2	CCR4-NOT transcription complex subunit 2
DYM	Dymeclin
LAGE3	EKC/KEOPS complex subunit LAGE3
LAMP1	Lysosome-associated membrane glycoprotein 1
MPV17	Protein Mpv17
SIX5	Homeobox protein SIX5
SOS1	Son of sevenless homolog 1

D **Down-regulated proteins (fold-change ≤ log₂ = −1.5)**

Gene	Protein name
BRIP1	Fanconi anemia group J protein
BTN3A1	Butyrophilin subfamily 3 member A1
GPALPP1	GPALPP motifs-containing protein 1
GXYLT1	Glucoside xylosyltransferase 1
HBA1	Hemoglobin subunit alpha
HELLS	Lymphoid-specific helicase
LSM7	U6 snRNA-associated Sm-like protein LSm7
MLX	Max-like protein X
SNRPG	Small nuclear ribonucleoprotein G
STAMBPL1	AMSH-like protease
TMEM199	Transmembrane protein 199
TMEM63B	CSC1-like protein 2
VMA21	Vacuolar ATPase assembly integral membrane protein VMA21
ZMYM2	Zinc finger MYM-type protein 2
ZNF668	Zinc finger protein 668

Figure S5: Whole proteome analysis of Levofloxacin in PDL-hTERT cells after 3 days of incubation. (**A**) Volcano plot depicting the proteome regulation of the low concentration versus the control. (**B**) Volcano plot of the high concentration versus the control. Threshold lines represent a $\log_2$ regulation of ±1 and $-\log_{10}$ (P-value) of 1.3 (two-sided two-sample t-test, n = 4 replicates per group). Subunits of the ETC complex I (blue) and complex IV (green) are highlighted. (**C**) Top up-regulated proteins in alphabetical order (threshold: fold-change ≥ $\log_2$ 1.5). (**D**) Top down-regulated proteins in alphabetical order (threshold: fold-change ≤ $\log_2$ –1.5).

Figure S6

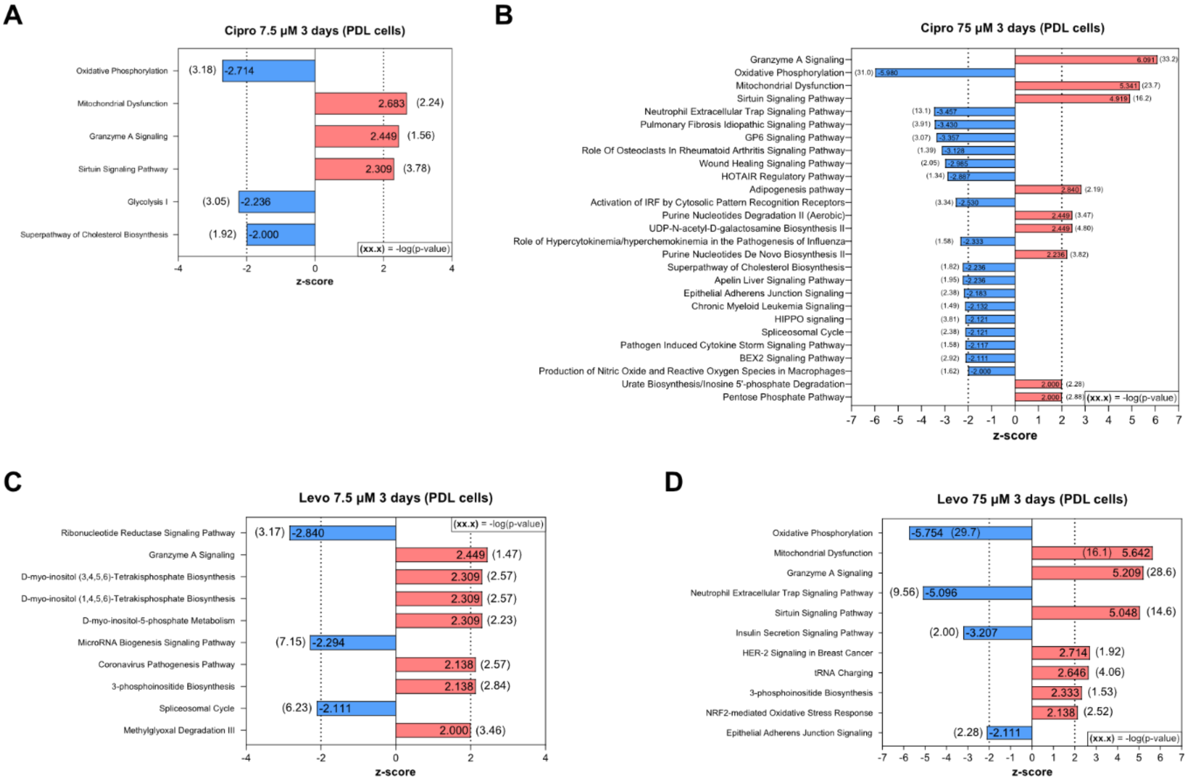

Figure S6: Proteome regulation pathway analyses of FQ-treated PDL cells after 3 days. Regulated pathways upon treatment with 7.5 µM Ciprofloxacin (**A**) and 75 µM Ciprofloxacin (**B**). Respective analyses of treatment with 7.5 µM Levofloxacin (**C**) and 75 µM Levofloxacin (**D**). The bar plots depict the intensity (z-score, threshold ±2) and statistical significance (– $\log_{10}$ *P*-value, in brackets) of the pathway regulation.

Figure S7

A

	IC_{50} HEK cells	IC_{50} PDL-hTERT cells
Cipro	> 150 µM	> 175 µM
Levo	> 400 µM	> 800 µM

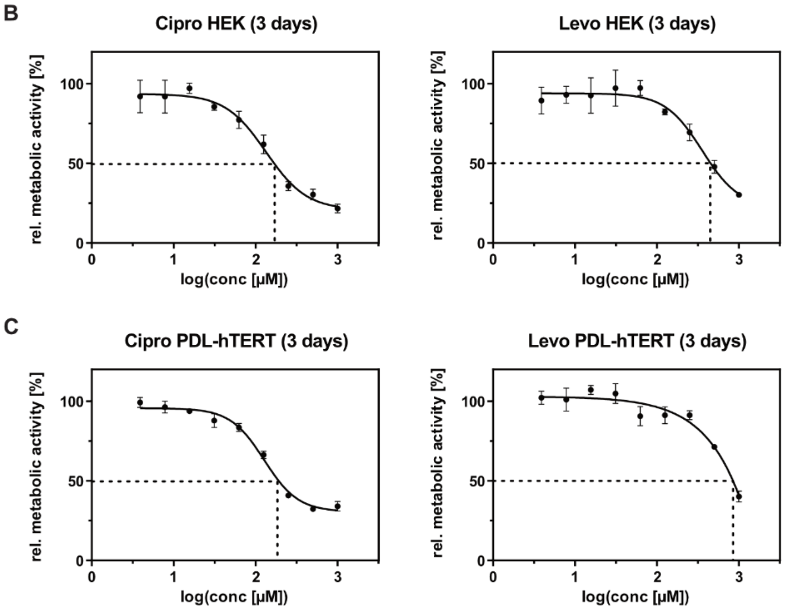

Figure S7: Relative metabolic activity after incubation with Ciprofloxacin and Levofloxacin for 3 days. (**A**) Approximate apparent IC_{50} values in HEK-293 and PDL-hTERT cells. MTT assay plots in HEK-293 (**B**) and PDL cells (**C**). As no complete inhibition of metabolic activity was reached in the tested solubility range of the compounds, the apparent IC_{50} values were approximated (dashed line) and given as approximate values in (**A**). The mean and SD of 3 replicates per condition is plotted and the graphs are representative of two independent experiments.

Figure S8

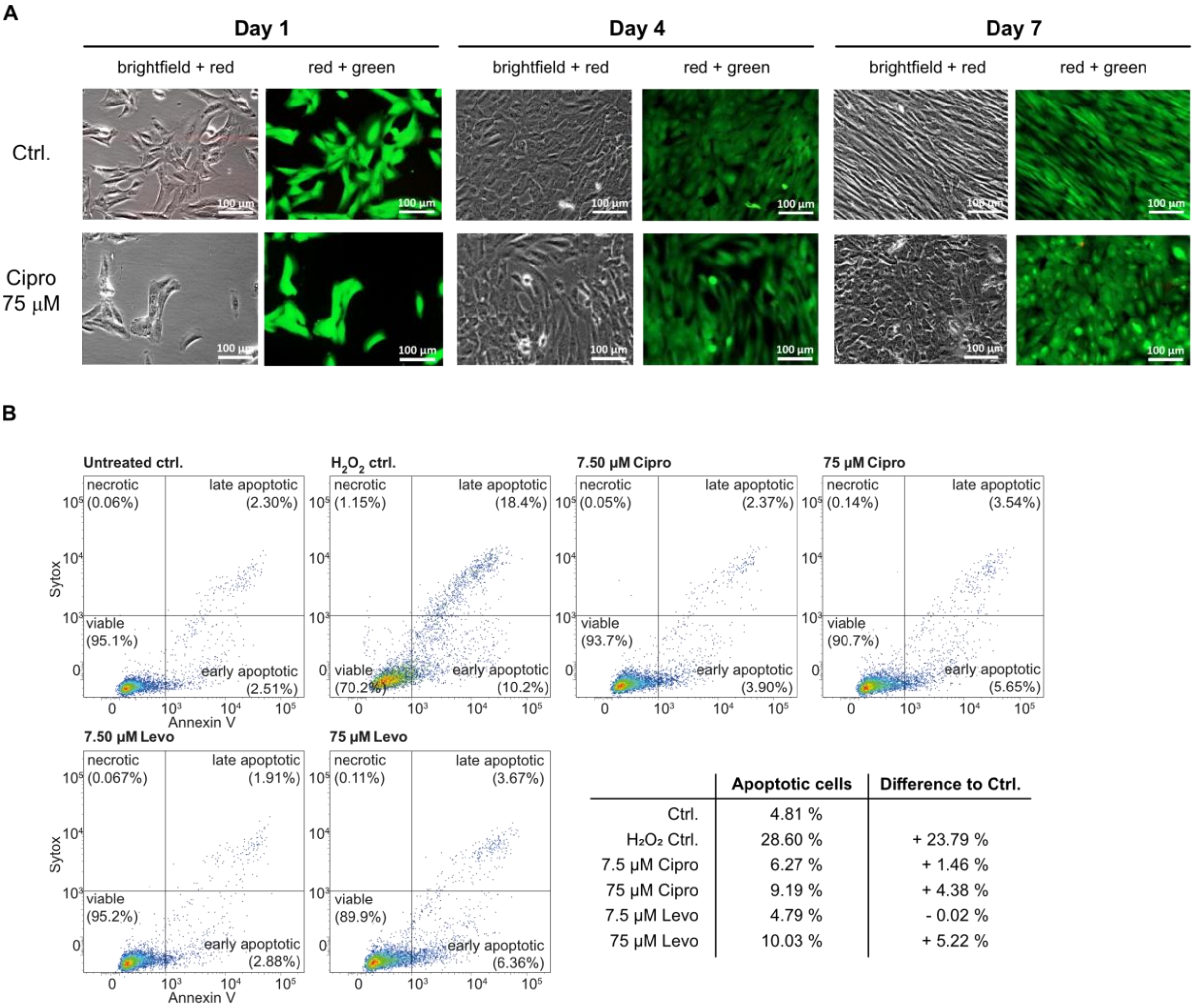

	Apoptotic cells	Difference to Ctrl.
Ctrl.	4.81 %	
H_2O_2 Ctrl.	28.60 %	+ 23.79 %
7.5 µM Cipro	6.27 %	+ 1.46 %
75 µM Cipro	9.19 %	+ 4.38 %
7.5 µM Levo	4.79 %	- 0.02 %
75 µM Levo	10.03 %	+ 5.22 %

Figure S8: Viability and morphology of Ciprofloxacin-treated PDL-hTERT cells. (**A**) Live/dead-Assay: Microscope pictures of Ciprofloxacin- and control-treated PDL-hTERT cells (20× magnification). Prolonged incubation of the cells for 7 days with Ciprofloxacin (75 µM) leads to cell rounding in comparison to control cells. Live, Calcein-AM-positive cells are labelled in green colour, while dead, Propidium Iodide-positive cells are labelled in red colour. Representative images derived are shown from three independent experiments (n=3). (**B**) FACS-based apoptosis assay in PDL-hTERT cells in presence of FQs (7.5 µM and 75 µM) for 3 days. Incubation with fluorescent Annexin V and Sytox dyes enable gating of cells in viable, early apoptotic, late apoptotic and necrotic populations. Vehicle control and H_2O_2-incubated (2 mM, 30 min, 37 °C) cells were used as control and 200.000 events measured per condition. Only minor, concentration-dependent increases of apoptotic populations (sum of early and late apoptotic states) were determined for both Ciprofloxacin and Levofloxacin.

Scheme S1

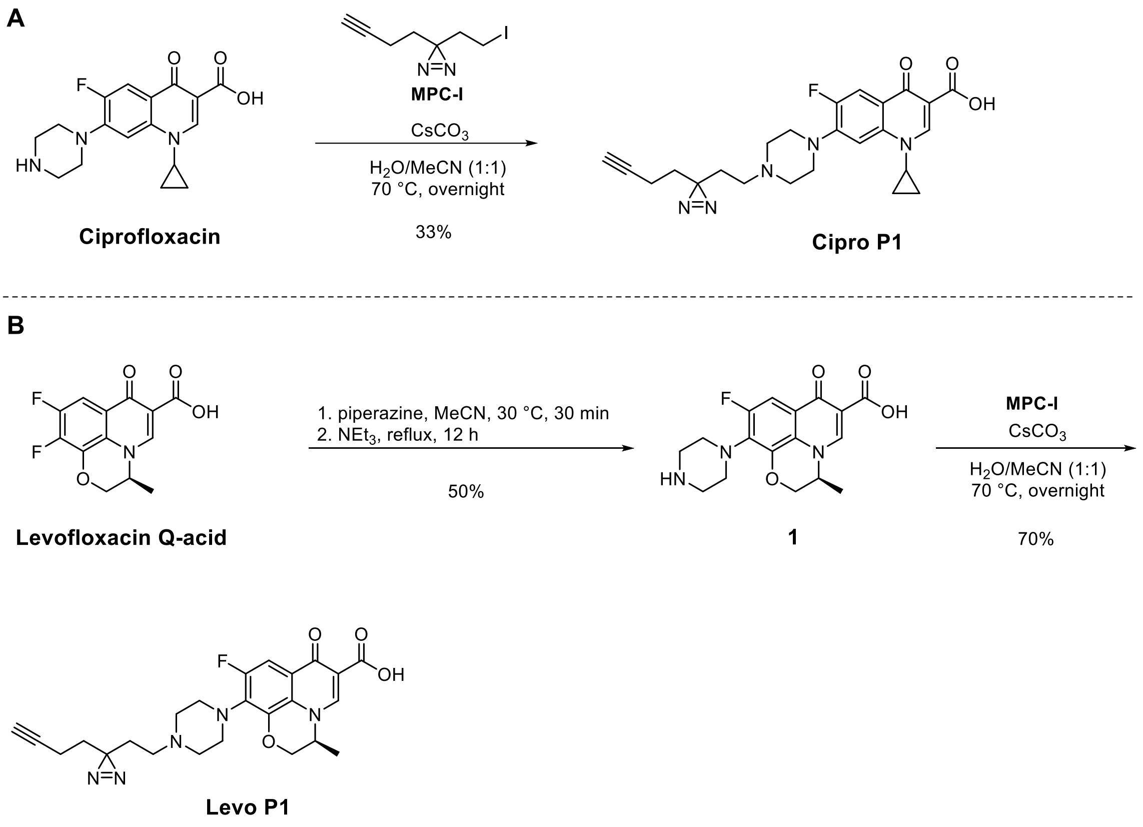

Scheme S1: Synthesis of probes **Cipro P1** and **Levo P1**. (**A**) Nucleophilic substitution with ciprofloxacin and iodo-minimalist photo-crosslinker **MPC-I** afforded **Cipro P1**. (**B**) Levofloxacin Q-acid was subjected to nucleophilic aromatic substitution conditions to install the piperazine moiety, followed by reaction with the photo-crosslinker **MPC-I** yielded **Levo P1**. The iodo-derivative of the minimalist photo-crosslinker **MPC-I** was synthesized according to literature procedures by *Li* et *al*.[4]

Scheme S2

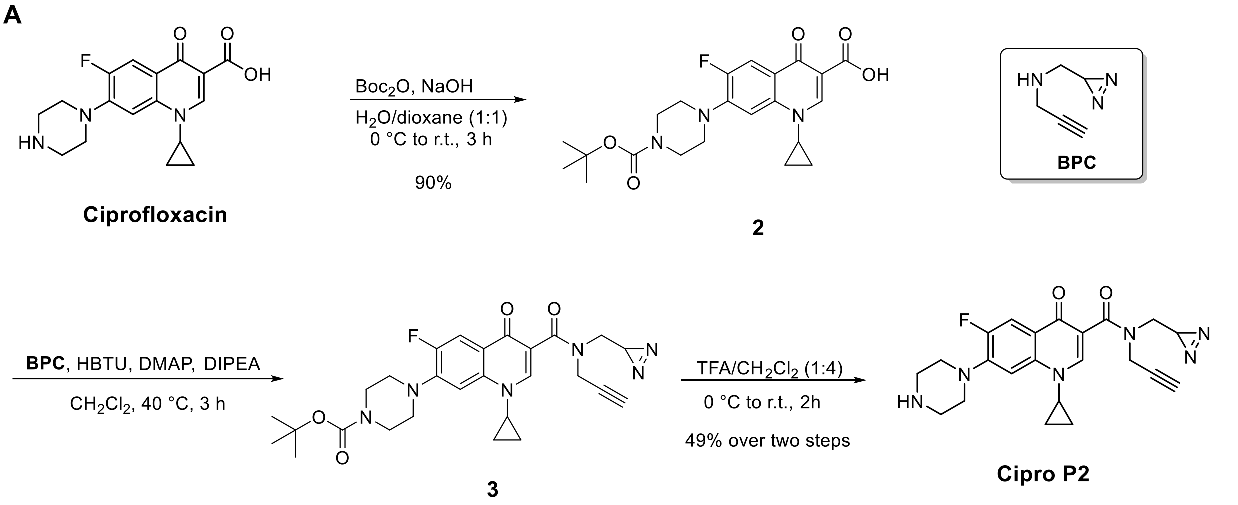

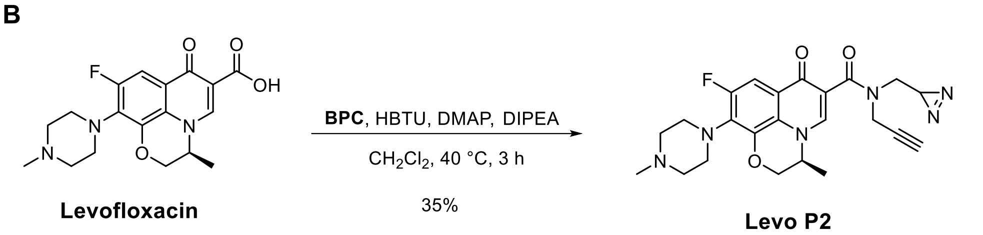

Scheme S2: Synthesis of probes **Cipro P2** and **Levo P2**. (**A**) Boc-protection of the piperazine moiety of Ciprofloxacin afforded **2**, enabling selective amide-coupling of the branched photo-crosslinker **BPC** and subsequent TFA-mediated deprotection to obtain **Cipro P2** in 44% yield over 3 steps. (**B**) Synthesis of **Levo P2** by amide-coupling of the branched photo-crosslinker **BPC** to Levofloxacin.

Figure S9

A

Organism	Ciprofloxacin	Levofloxacin	Cipro P1	Levo P1	Cipro P2	Levo P2
S. aureus NCTC8325	62 nM	31 nM	780 nM (12.5×)	780 nM (25×)	> 100 **µM** (> 1600×)	100 **µM** (3225×)
E. coli MM28	15 - 31 n**M**	62 nM	3.1 **µM** (100×)	6.25 **µM** (100×)	50 **µM** (1600×)	25 **µM** (400×)
E. coli RFM795	8 n**M**	15 - 31 nM	31 n**M** (4×)	125 n**M** (4×)	12.5 **µM** (1560×)	3 **µM** (100×)

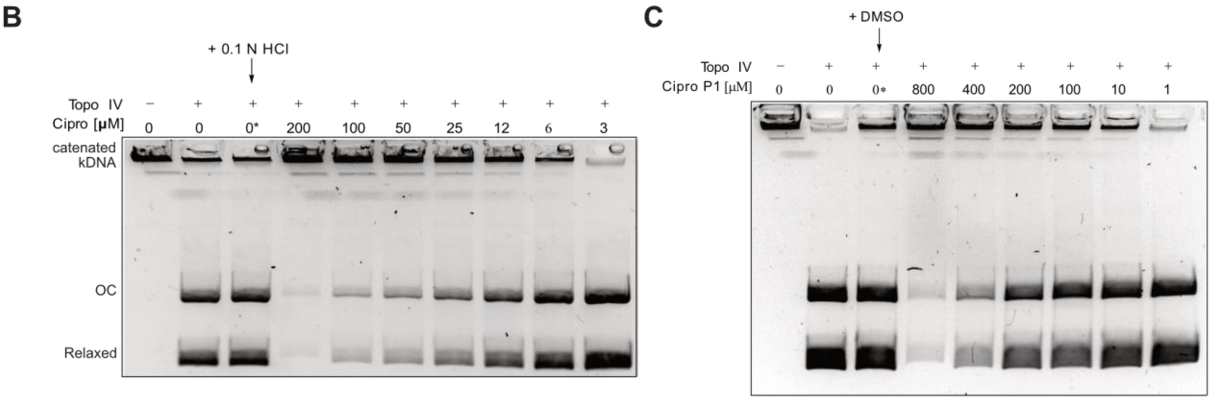

Figure S9: (**A**) Minimal Inhibitory Concentrations (MIC) of Ciprofloxacin, Levofloxacin and their derived probes. The x-fold decrease in potency of the probes compared to their parent compound is given in brackets. Topoisomerase IV activity assay in presence of Ciprofloxacin (**B**) and **Cipro P1** (**C**), respectively. Catenated kDNA is decatenated and due to the presence of an intercalator in the agarose gel resolved in their open circular (OC) and relaxed circular forms. The approximate apparent IC_{50} value of Ciprofloxacin is 25 µM, consistent with literature values.[5,6] **Cipro P1** also inhibits the topoisomerase IV activity, the apparent approximate IC_{50} value is up to 16-fold higher.

Figure S10

A

	Cipro P1	Cipro P2	Levo P1	Levo P2
HEK	> 750 µM	> 400 µM	> 950 µM	> 350 µM
PDL	> 550 µM	> 120 µM	> 380 µM	> 150 µM

B

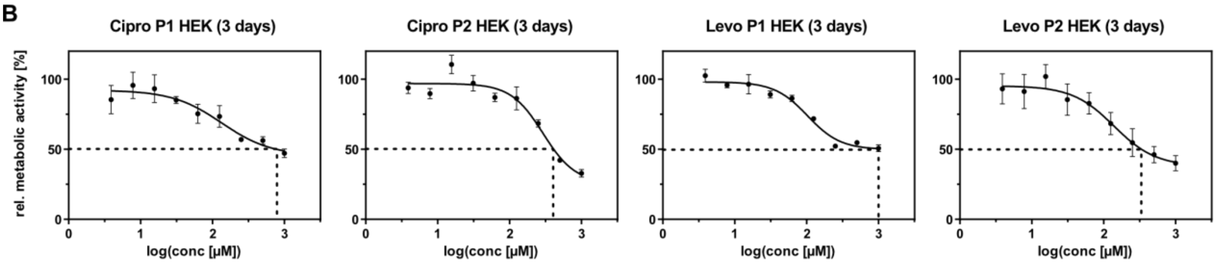

C

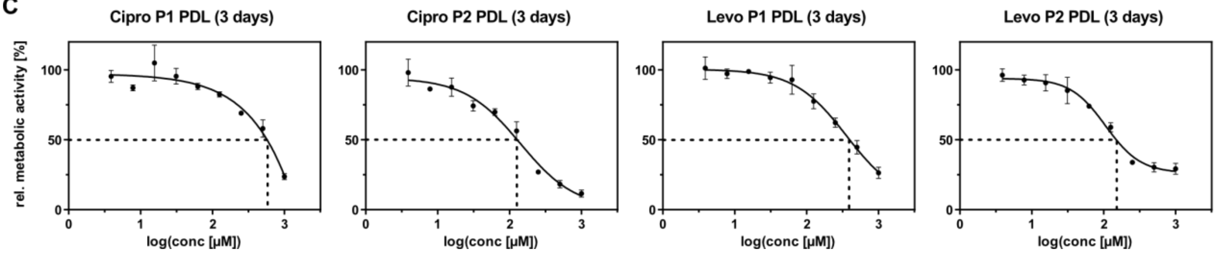

Figure S10: Relative metabolic activity after incubation with the four affinity probes for 3 days. (**A**) Approximate apparent IC_{50} values in HEK-293 and PDL-hTERT cells. Corresponding MTT assay plots in HEK-293 (**B**) and PDL-hTERT cells (**C**). As no complete inhibition of metabolic activity was reached in the tested solubility range of the compounds up to 1 mM, the apparent IC_{50} values were approximated (dashed line) and given as approximate values in (**A**). The mean and SD of 3 replicates per condition are plotted and the graphs are representative of two independent experiments.

Figure S11

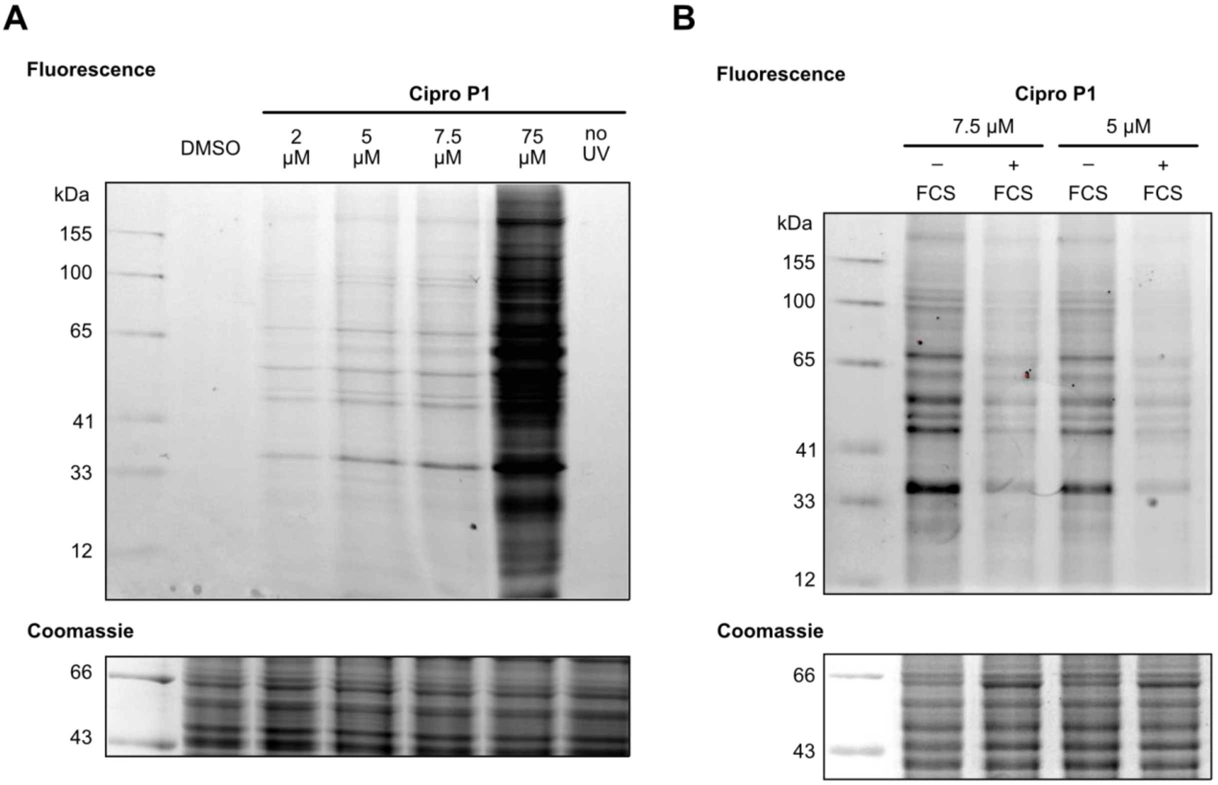

Figure S11: Gel-based analytical AfBPP with **Cipro P1** in HEK-293 cells. (**A**) Concentration-dependent labelling pattern after incubation with **Cipro P1** for 1 h at 37 °C and UV irradiation for 15 min at 360 nm and subsequent lysis, CuAAC-mediated attachment of TAMRA-fluorophore and SDS-PAGE with fluorescent-scanning read-out. Vehicle- and non-irradiated controls showed no labelling. The corresponding Coomassie-stain is depicted as loading control. (**B**) Analytical AfBPP experiment comparing in situ labelling in FCS-free and FCS-containing medium verifying the desired effect of increased labelling intensity in FCS-free medium without altering the distinct labelling pattern.

Figure S12

A

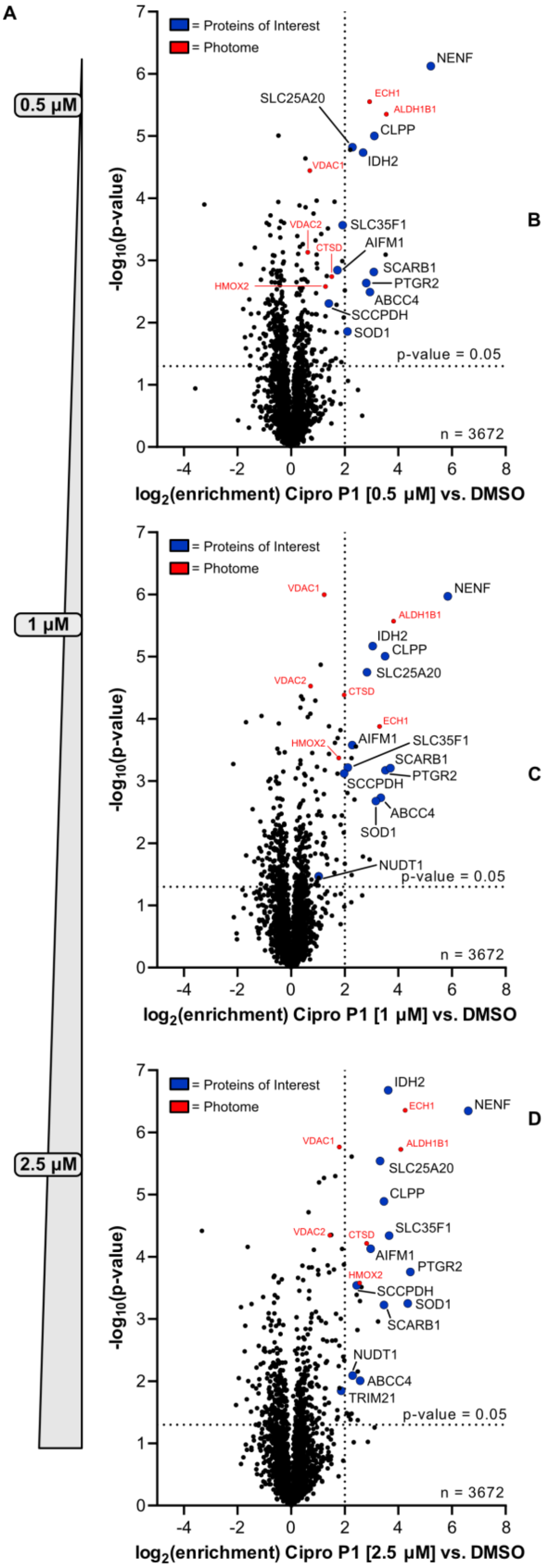

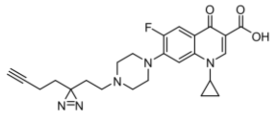

Cipro P1

AfBPP in HEK cells

B

Protein of Interests

Gene	Protein
NENF	Neudesin
CLPP	ATP-dependent Clp protease proteolytic subunit, mitochondrial
IDH2	Isocitrate dehydrogenase [NADP], mitochondrial
AIFM1	Apoptosis-inducing factor 1, mitochondrial
SCARB1	Scavenger receptor class B member 1
PTGR2	Prostaglandin reductase 2
SLC25A20	Mitochondrial carnitine/acylcarnitine carrier protein
SLC35F1	Solute carrier family 35 member F1
ABCC4	ATP-binding cassette sub-family C member 4
SOD1	Superoxide dismutase
SCCPDH	Saccharopine dehydrogenase-like oxidoreductase
NUDT1	Oxidized purine nucleoside triphosphate hydrolase (MTH1)
TRIM21	E3 ubiquitin-protein ligase TRIM21

C

Photome hits

Gene	Protein
ALDH1B1	Aldehyde dehydrogenase X, mitochondrial
VDAC1	Voltage-dependent anion-selective channel protein 1
VDAC2	Voltage-dependent anion-selective channel protein 2
ECH1	Delta(3,5)-Delta(2,4)-dienoyl-CoA isomerase, mitochondrial
CTSD	Cathepsin D
HMOX2	Heme oxygenase 2

D

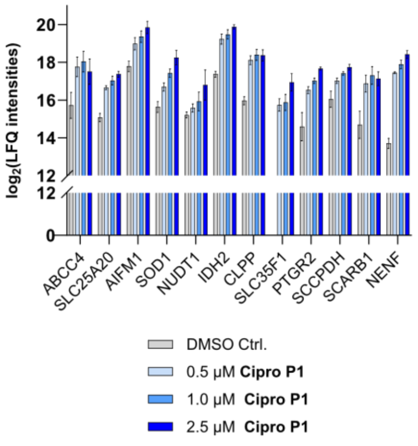

Figure S12: Preparative AfBPP of **Cipro P1** in HEK-293 cells. (**A**) Concentration-dependent volcano plots of **Cipro P1** (0.5, 1.0 and 2.5 µM for 1 h at 37 °C) versus DMSO control. Threshold lines indicate $\log_2$ enrichment ≥ 2 and the statistical significance –$\log_{10}$(P value) ≥ 1.3 (two-sided two-sample t-test, n = 4 replicates per group). Literature-known unspecific photome hits are depicted in red.[7] Proteins of interest are highlighted in blue. (**B**) Corresponding table of potential off-target proteins of interest. (**C**) Table of corresponding reported photome hits. (**D**) Profile plots of the mean $\log_2$(LFQ intensities) and the respective SD of the 12 proteins of interest to illustrate concentration-dependent enrichment.

Figure S13

A

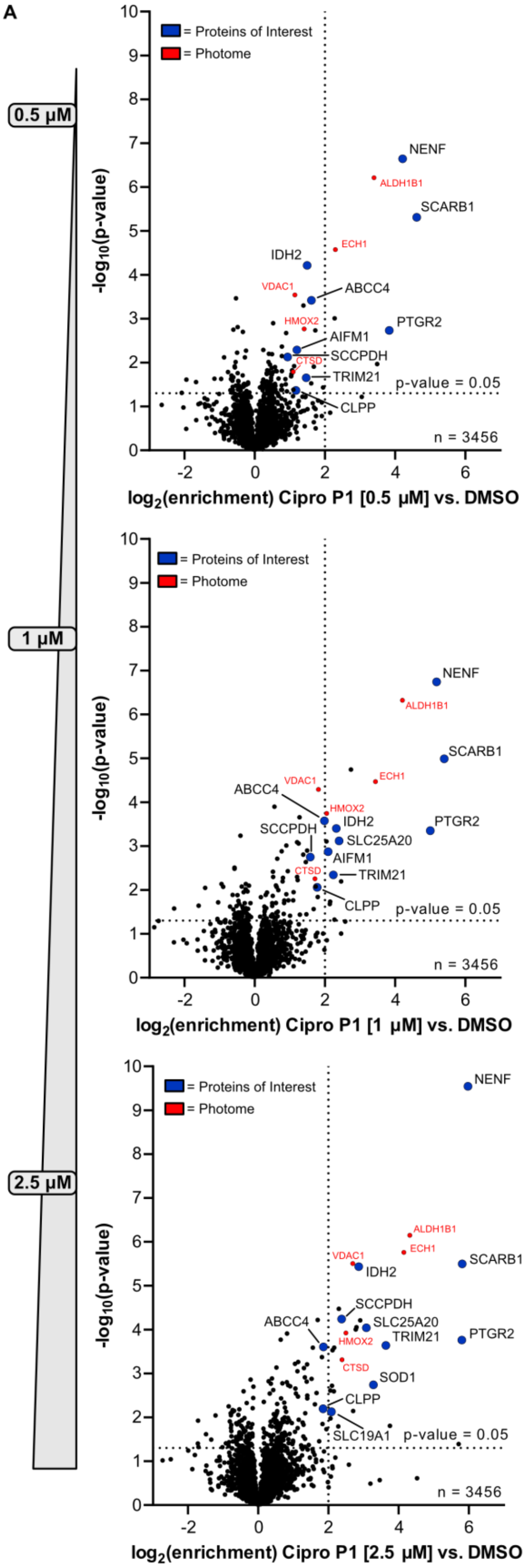

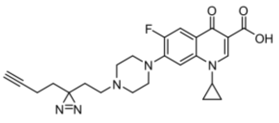

B

Protein of Interests

Gene	Protein
NENF	Neudesin
SCARB1	Scavenger receptor class B member 1
PTGR2	Prostaglandin reductase 2
IDH2	Isocitrate dehydrogenase [NADP], mitochondrial
AIFM1	Apoptosis-inducing factor 1, mitochondrial
ABCC4	ATP-binding cassette sub-family C member 4
SCCPDH	Saccharopine dehydrogenase-like oxidoreductase
TRIM21	E3 ubiquitin-protein ligase TRIM21
CLPP	ATP-dependent Clp protease proteolytic subunit, mitochondrial
SLC25A20	Mitochondrial carnitine/acylcarnitine carrier protein

C

Photome hits

Gene	Protein
ALDH1B1	Aldehyde dehydrogenase X, mitochondrial
VDAC1	Voltage-dependent anion-selective channel protein 1
ECH1	Delta(3,5)-Delta(2,4)-dienoyl-CoA isomerase, mitochondrial
CTSD	Cathepsin D
HMOX2	Heme oxygenase 2

D

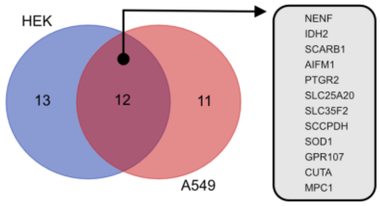

Figure S13: Preparative AfBPP of **Cipro P1** in A549 cells. (**A**) Concentration-dependent volcano plots of **Cipro P1** (0.5, 1.0 and 2.5 µM for 1 h at 37 °C) versus DMSO control. Threshold lines indicate $\log_2$ enrichment ≥ 2 and the statistical significance $-\log_{10}$(*P* value) ≥ 1.3 (two-sided two-sample *t*-test, *n* = 4 replicates per group). Literature-known unspecific photome hits are depicted in red.[7] Proteins of interest are highlighted in blue. (**B**) Corresponding table of potential off-target proteins of interest. (**C**) Table of corresponding reported photome hits. (**D**) Venn-diagram showing the overlapping proteins of interest enriched by **Cipro P1** in HEK-293 and A549 cell lines in accordance with the standard thresholds.

Figure S14

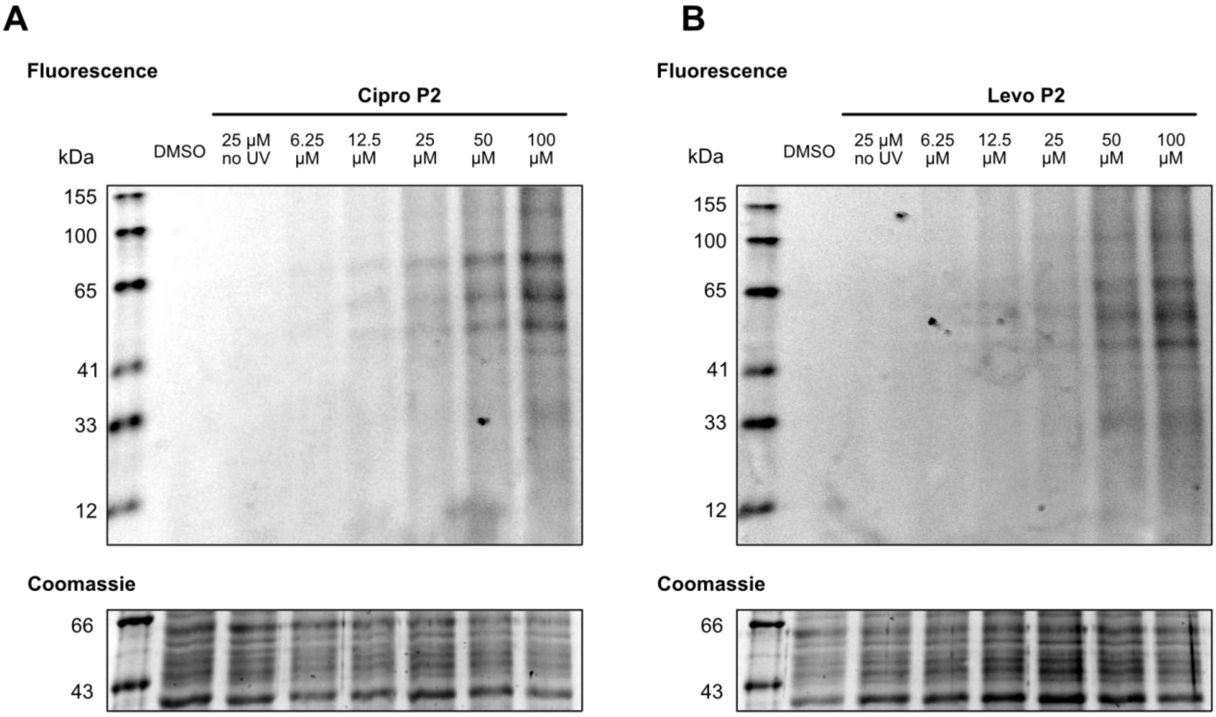

Figure S 14: Gel-based analytical AfBPP with **Cipro P2** and **Levo P2** in PDL cells. (**A**) Concentration-dependent labelling pattern after incubation with **Cipro P2.** (**B**) Concentration-dependent labelling pattern after incubation with **Levo P2.** As reported, the branched photo-crosslinker used in the **P2** probes needs higher concentrations compared to **P1** probes for sufficient labelling.[8] Cells were incubated for 1 h at 37 °C and after UV irradiation for 10 min at 300 nm and lysed, TAMRA-fluorophore appended via CuAAC chemistry and SDS-PAGE with fluorescent-scanning read-out performed. Vehicle- and non-irradiated controls showed no labelling. The corresponding Coomassie-stain is depicted as loading control.

Figure S15

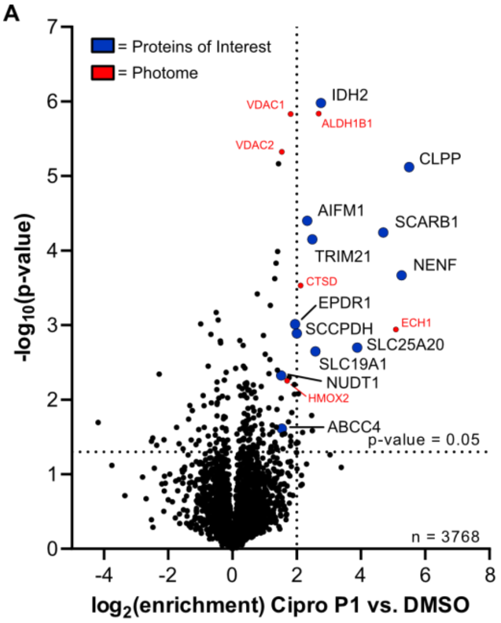

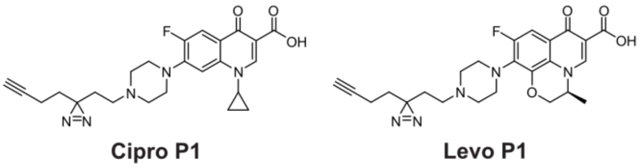

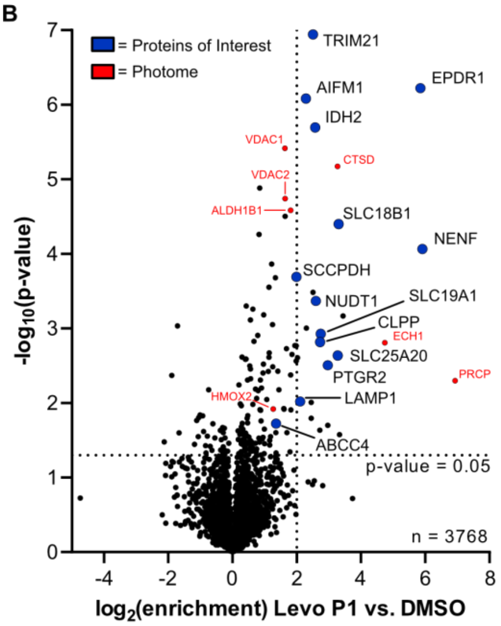

C

Protein of Interests

Gene	Protein
IDH2	Isocitrate dehydrogenase [NADP], mitochondrial
CLPP	ATP-dependent Clp protease proteolytic subunit, mitochondrial
AIFM1	Apoptosis-inducing factor 1, mitochondrial
SCARB1	Scavenger receptor class B member 1
TRIM21	E3 ubiquitin-protein ligase TRIM21
NENF	Neudesin
SLC19A1	Reduced folate transporter
SLC25A20	Mitochondrial carnitine/acylcarnitine carrier protein
NUDT1	Oxidized purine nucleoside triphosphate hydrolase (MTH1)
EPDR1	Mammalian ependymin-related protein 1
SLC18B1	MFS-type transporter SLC18B1
PTGR2	Prostaglandin reductase 2
LAMP1	Lysosome-associated membrane glycoprotein 1
SCCPDH	Saccharopine dehydrogenase-like oxidoreductase
ABCC4	ATP-binding cassette sub-family C member 4

D

Photome hits

Gene	Protein
VDAC1	Voltage-dependent anion-selective channel protein 1
VDAC2	Voltage-dependent anion-selective channel protein 2
ALDH1B1	Aldehyde dehydrogenase X, mitochondrial
CTSD	Cathepsin D
ECH1	Delta(3,5)-Delta(2,4)-dienoyl-CoA isomerase, mitochondrial
HMOX2	Heme oxygenase 2
PRCP	Lysosomal Pro-X carboxypeptidase

Figure S15: Preparative AfBPP of **P1** probes in PDL-hTERT cells. Volcano plot **of Cipro P1** (**A**) and **Levo P1** (**B**) versus the control. Cells were treated with 5 µM of the respective probe and incubated for 1 h at 37 °C before UV irradiation at 360 nm. Threshold lines indicate $\log_2$ enrichment ≥ 2 and the statistical significance $-\log_{10}$(*P* value) ≥ 1.3 (two-sided two-sample *t*-test, *n* = 4 replicates per group). Proteins of interest are highlighted in blue and listed in (**C**). Literature-known unspecific photome hits are depicted in red and listed in (**D**).[7]

Figure S16

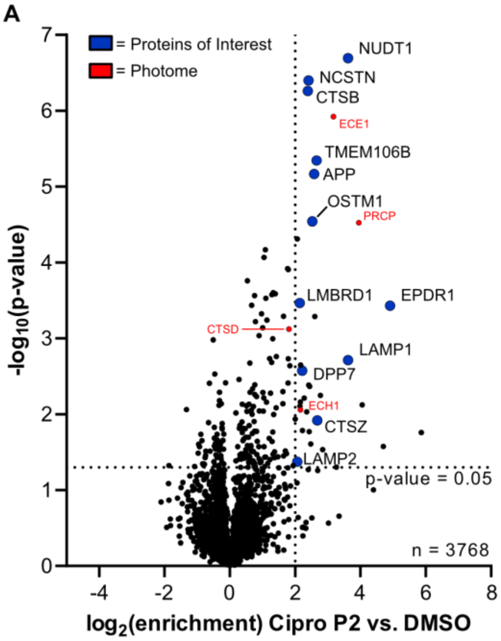

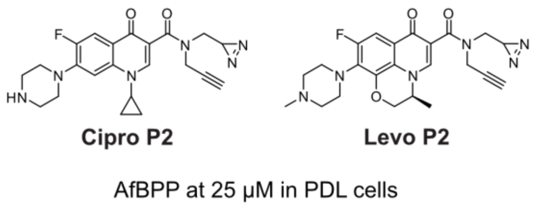

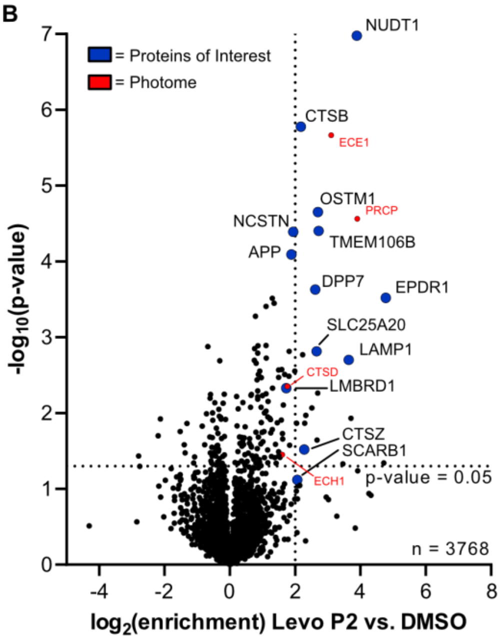

C

Protein of Interests

Gene	Protein
NUDT1	Oxidized purine nucleoside triphosphate hydrolase (MTH1)
* NCSTN	Lysosomal Pro-X carboxypeptidase
* CTSB	Mammalian ependymin-related protein 1
* TMEM106B	Transmembrane protein 106B
APP	Amyloid-beta precursor protein
* OSTM1	Osteopetrosis-associated transmembrane protein 1
* LMBRD1	Lysosomal cobalamin transport escort protein LMBD1
* EPDR1	Mammalian ependymin-related protein 1
* LAMP1	Lysosome-associated membrane glycoprotein 1
* LAMP2	Lysosome-associated membrane glycoprotein 2
* DPP7	Dipeptidyl peptidase 2
* CTSZ	Cathepsin Z

D

Photome hits

Gene	Protein
* CTSD	Cathepsin D
ECH1	Delta(3,5)-Delta(2,4)-dienoyl-CoA isomerase, mitochondrial
* PRCP	Lysosomal Pro-X carboxypeptidase
ECE1	Endothelin-converting enzyme 1

* = Protein localized in the lysosome

Figure S16: Preparative AfBPP of **P2** probes in PDL-hTERT cells. Volcano plot **of Cipro P2** (**A**) and **Levo P2** (**B**) versus the control. Cells were treated with 25 µM of the respective probe and incubated for 1 h at 37 °C before UV irradiation at 300 nm. Threshold lines indicate $\log_2$ enrichment ≥ 2 and the statistical significance $-\log_{10}(P$ value) ≥ 1.3 (two-sided two-sample *t*-test, *n* = 4 replicates per group). Proteins of interest are highlighted in blue and listed in (**C**). The majority of enriched proteins are lysosomal and highlighted by an asterisk. Literature-known unspecific photome hits are depicted in red and listed in (**D**).[7]

Figure S17

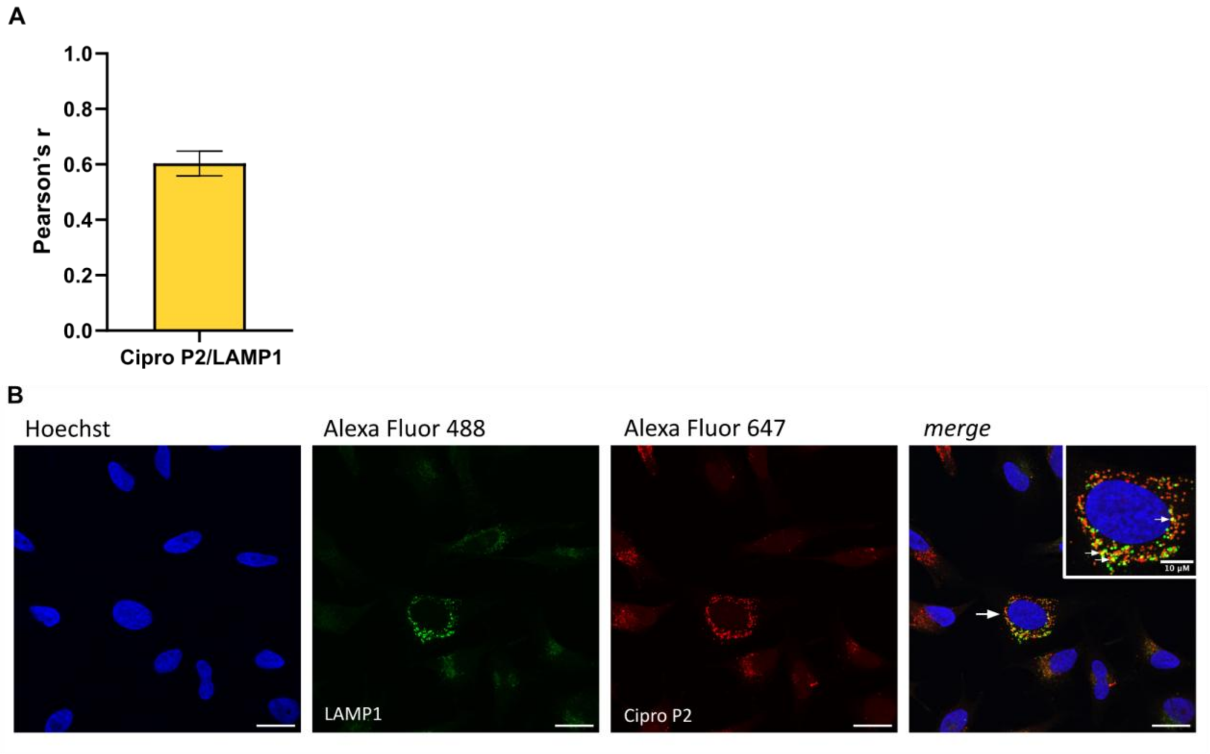

Figure S17: Lysosomal localization of **Cipro P2**. (**A**) Strong pearson´s r correlation of **Cipro P2** and the lysosome-associated membrane glycoprotein 1 (LAMP1) indicating strong co-localization in immunocytochemistry studies in HeLa cells. The mean and SD of 3 independent experiments is plotted with 50 cells analysed in each experiment. (**B**) Representative immunocytochemistry pictures demonstrating **Cipro P2** and LAMP1 co-localization. Nuclei were stained with Hoechst dye, LAMP1 primary antibody was visualized with an Alexa fluor 488-conjugated secondary antibody and Alexa Fluor 647 appended to the alkyne handle of **Cipro P2** *via* CuAAC chemistry. The merging of the channels revealed strong co-localization. The scale indicates 20 µm.

Figure S18

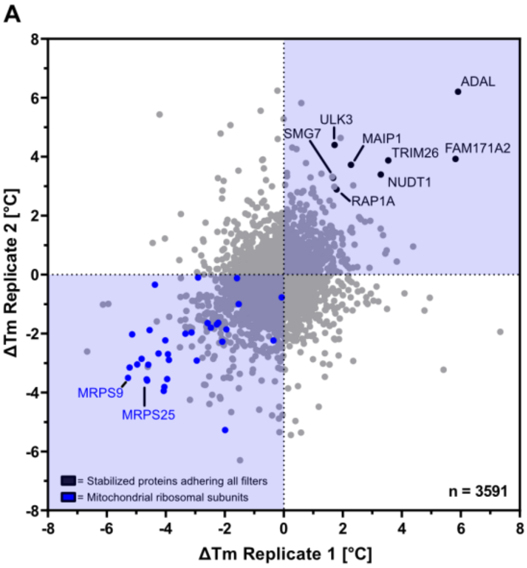

B

Destabilized proteins			
Gene name	ΔT_m Rep. 1	ΔT_m Rep. 2	Protein name
ZNF574	– 3.18	– 4.25	Zinc finger protein 574
MRPS22	– 4.97	– 3.05	28S ribosomal protein S22, mitochondrial
RAD51C	– 2.53	– 3.45	DNA repair protein RAD51 homolog 3
MRPS12	– 4.07	– 3.95	28S ribosomal protein S12, mitochondrial
F8A1	– 6.67	– 2.61	40-kDa huntingtin-associated protein
DAP3	– 4.25	– 2.68	28S ribosomal protein S29, mitochondrial
MRPS25	– 4.63	– 3.59	28S ribosomal protein S25, mitochondrial
MRPS5	– 5.23	– 3.15	28S ribosomal protein S5, mitochondrial
MRPS15	– 4.05	– 3.81	28S ribosomal protein S15, mitochondrial
MRPS9	– 5.28	– 3.51	28S ribosomal protein S9, mitochondrial
MRPS39	– 4.60	– 3.06	28S ribosomal protein S39, mitochondrial
MRPS26	– 4.65	– 3.55	28S ribosomal protein S26, mitochondrial
MED11	– 4.62	– 3.13	Mediator of RNA polymerase II transcription subunit 11
SIK3	– 2.61	– 1.84	Serine/threonine-protein kinase SIK3
MRPS17	– 3.96	– 3.55	28S ribosomal protein S17, mitochondrial

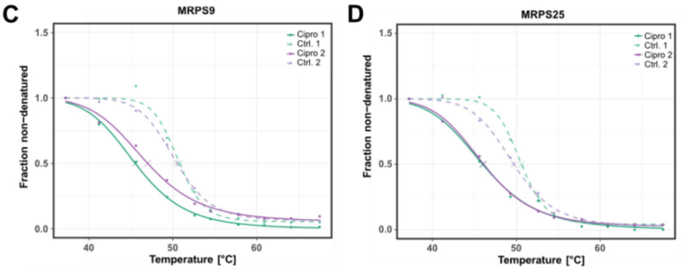

Figure S18: Destabilization effects in TPP with Ciprofloxacin (75 µM, 1 h, 37 °C) in HEK-293 cells. (**A**) Scatter plot of the melting point differences of both Ciprofloxacin-treated replicates in relation to their vehicle-treated replicates. Stabilized proteins adhering to all filters are labelled in black. Mito-ribosomal subunits are depicted in blue and were found to be significantly destabilized or their expression affected. (**B**) Table of destabilized proteins adhering to all filters. Inferred melting curves of exemplary destabilized mito-ribosomal subunits MRPS9 (**C**) and MRPS25 (**D**).

Figure S19

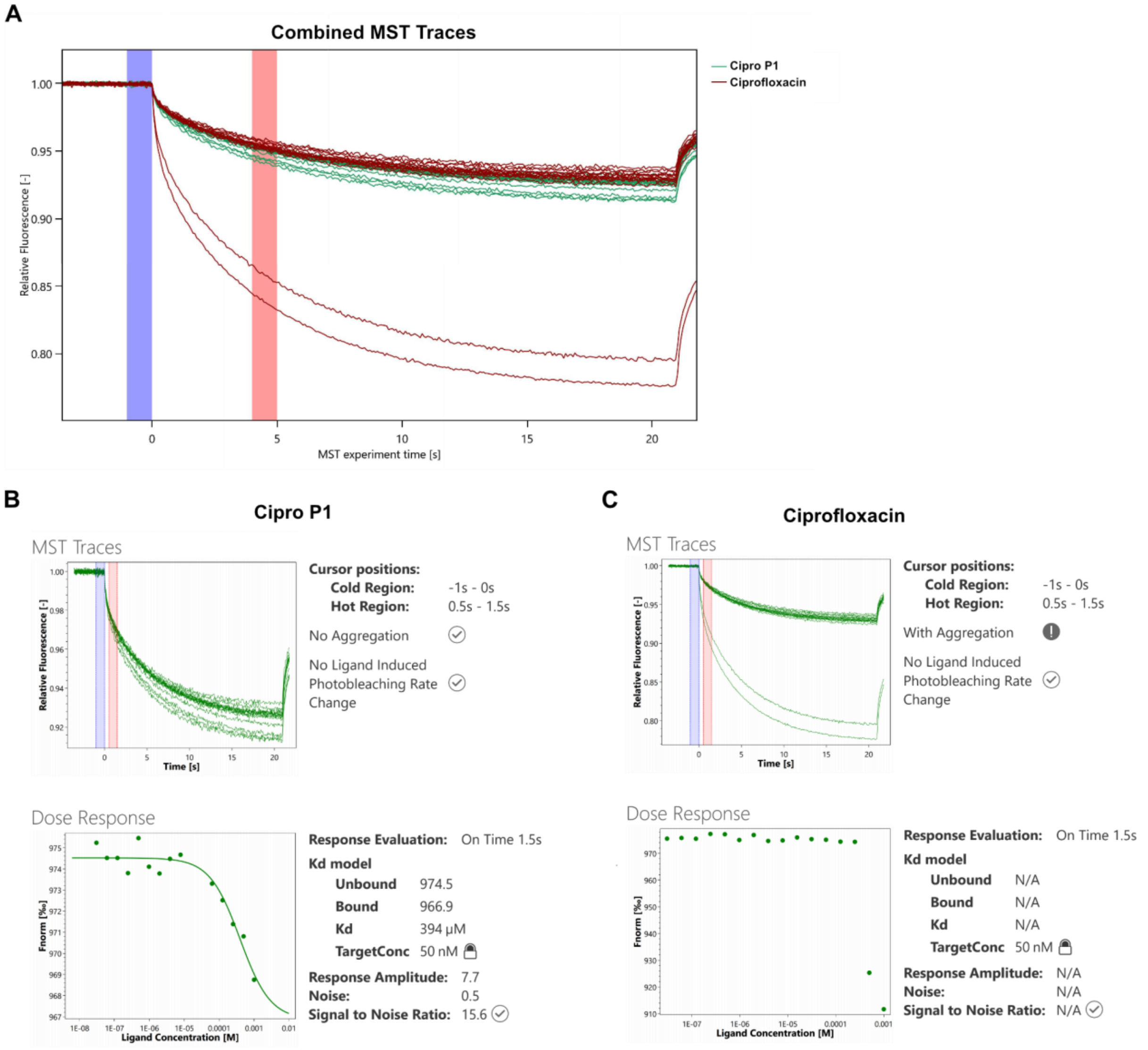

Figure S19: Investigation of Ciprofloxacin and **Cipro P1** binding to SCARB1 via Microscale thermophoresis (MST). (**A**) The combined MST traces of **Cipro P1** and Ciprofloxacin of all capillaries are plotted. Fluorescence shifts of the highest Ciprofloxacin concentrations (500 µM and 1 mM) were not concentration-dependent and, therefore, likely due to compound precipitation and/or capillary wall adsorption. (**B**) MST traces and inferred dose response of **Cipro P1**. The cold and hot regions are depicted in blue and red, respectively. A binding affinity (K_d) of 394 µM was determined by the acquisition software (MO.Control, v1.6, *NanoTemper*). (**C**) MST traces and inferred dose response of Ciprofloxacin. As no robust dose-response was detected and the two highest concentrations resulted in precipitation, no binding affinity for SCARB1 could be inferred. The experiment was analysed with MO Affinity Analysis (v2.3, *NanoTemper*).

Figure S20

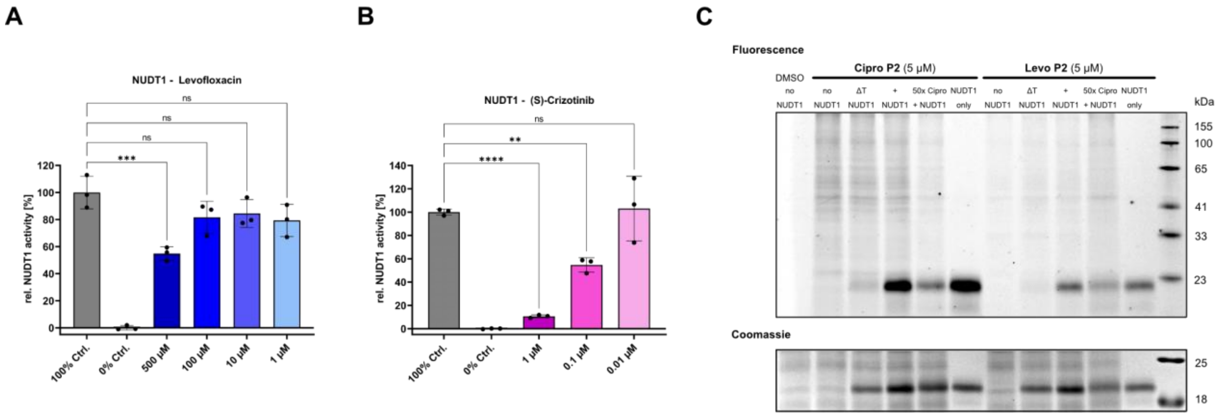

Figure S20: NUDT1 target validation studies. (**A**) In vitro activity assay of NUDT1 with Levofloxacin revealing significant inhibition only at the highest tested concentration (500 µM) that is likely not physiologically relevant. (**B**) Respective activity assay in presence of the selective inhibitor (*S*)-crizotinib, featuring nanomolar inhibition in accordance with the literature and indicating the fidelity of the assay.[9] The mean and SD of 3 replicates per condition is plotted. The graphs are representative of three independent experiments. In the bar plots the statistical relevance based on one-way ANOVA with Dunnett´s multiple comparison test is depicted (ns meaning P-value > 0.05, * P-value ≤ 0.05, ** P-value ≤ 0.01, *** P-value ≤ 0.001, **** P-value ≤ 0.0001). (**C**) Complete gel picture of analytical AfBPP of recombinant NUDT1 spiked into a human cell lysate background by **Cipro P2** and **Levo P2**. The labelling is abolished in the heat denatured NUDT1 control (ΔT) and partially outcompeted by the parent FQ, further validating NUDT1 engagement by the two FQs. The corresponding Coomassie-stain is depicted as loading control.

Figure S21

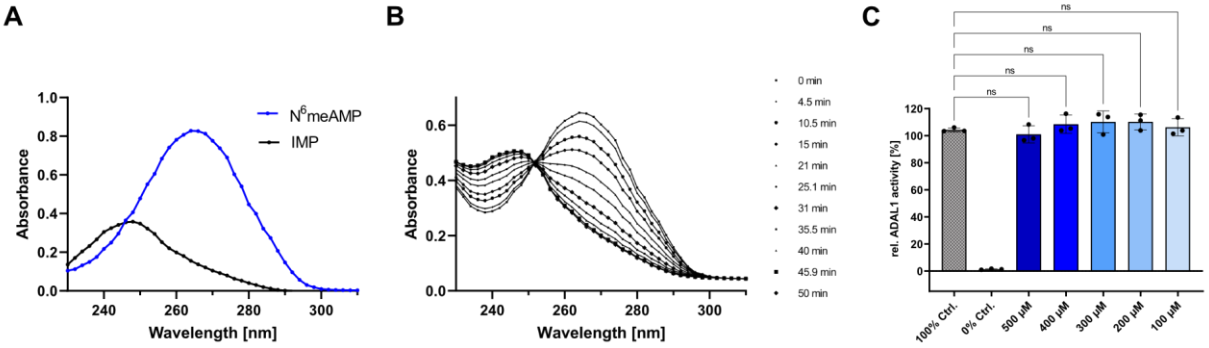

Figure S21: Target validation studies of ADAL using in vitro activity assays monitoring the absorbance shift of the substrate N^6-methyl-AMP to inosine monophosphate (IMP). (**A**) Absorbance profile of N^6-methyl-AMP (blue) and IMP (black). (**B**) Time-dependent absorbance shift monitoring the enzymatic reaction of N^6-methyl-AMP to IMP by ADAL. (**C**) Activity assay in presence of Levofloxacin, showing no significant effect. The graph is representative of two independent experiments. In the bar plot the statistical relevance based on one-way ANOVA with Dunnett´s multiple comparison test is depicted (ns meaning P-value > 0.05, * P-value ≤ 0.05, ** P-value ≤ 0.01, *** P-value ≤ 0.001, **** P-value ≤ 0.0001).

Figure S22

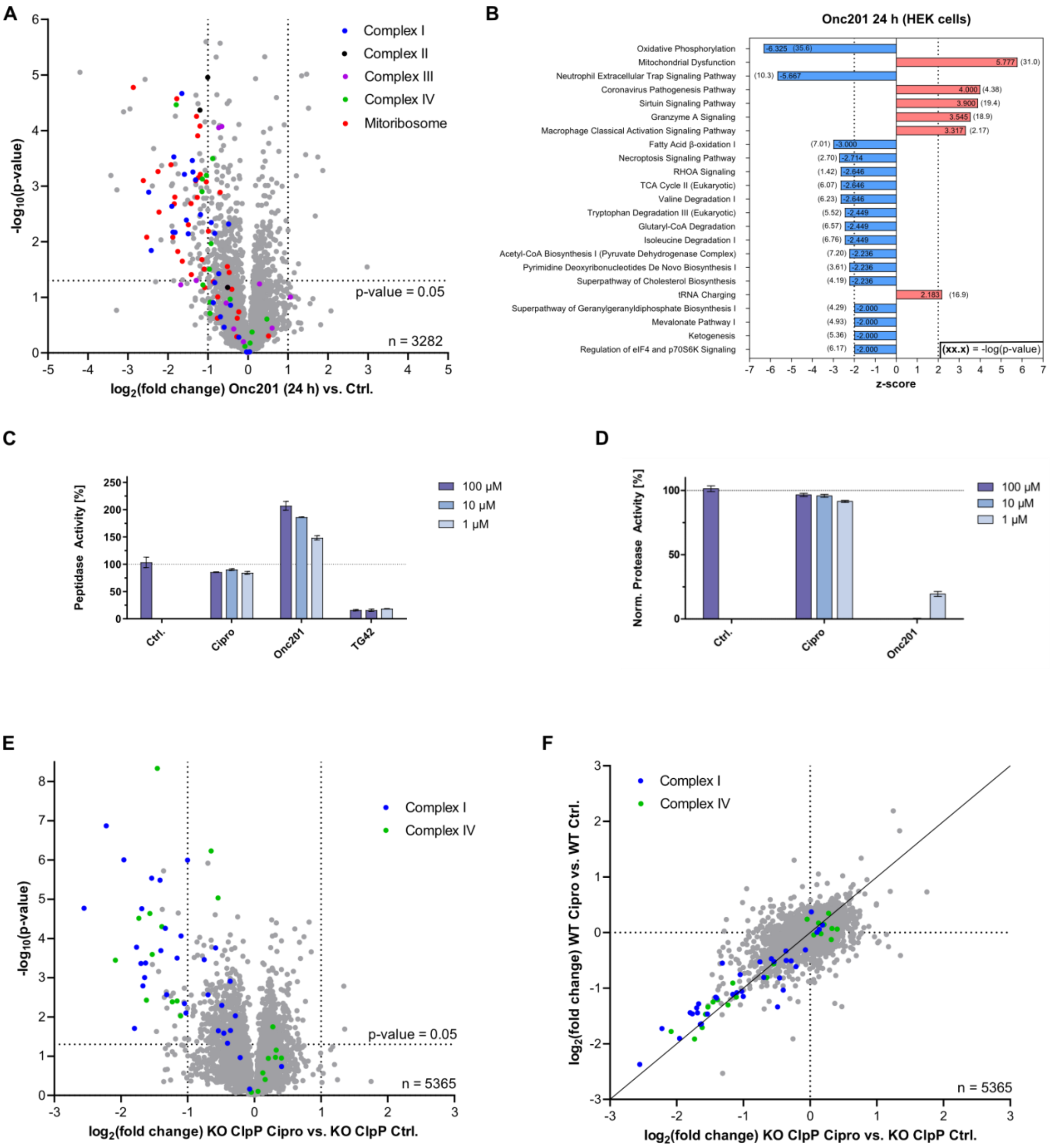

Figure S22: Target devalidation of ClpP. (**A**) Full proteome analysis of Onc201-treated HEK293 cells. The volcano plot depicts up- and downregulation or proteins on a log_2 scale after treatment with Onc201 (1 µM) for 24 h compared to vehicle-control treated cells. (**B**) Corresponding pathway analysis of the dysregulated proteome. The bar plots depict the intensity (z-score, threshold ±2) and statistical significance (– log_{10} *P*-value, in brackets) of the pathway regulation. Interestingly, the pattern of downregulated proteins and the affected pathways are strikingly similar to Ciprofloxacin treated cells (compare **Figure S6B**) except that, in general, more mitochondrial pathways seem to be affected. (**C**) ClpP peptidase activity assay in presence of Ciprofloxacin. No significant and concentration-dependent effect was detected. Known overactivator Onc201 leads to strong concentration-dependent overactivation, whereas the published compound TG42 strongly inhibits ClpP peptidase activity.[10,11] The mean and SD of 4 replicates per condition is plotted. The graph is representative of two independent experiments. (**D**) ClpXP protease activity assay in presence of Ciprofloxacin and Onc201. Ciprofloxacin did not affect the protease activity, whereas Onc201 strongly inhibits the activity, by preventing the formation of the ClpXP protease complex. The mean and SD of 4 replicates per condition is plotted. The graph is representative of two independent experiments. (**E**) Whole proteome analysis of ClpP knock-out HEK293 cells. The volcano plot depicts up- and downregulation or proteins on a log_2 scale after treatment with Ciprofloxacin (75 µM) for 3 days compared to vehicle-control treated cells. Despite the genetic knock-out of ClpP the presence of Ciprofloxacin leads to a strong downregulation of ETC complex I (blue), complex III (black) and complex IV (green). Thus, a potential interaction of Ciprofloxacin with ClpP is not causing the observed phenotype. (**F**) Scatter plot comparing treated versus untreated log_2(fold-changes) in wild-type and ClpP KO cells, additionally showing the strong correlation and similarity.

Figure S23

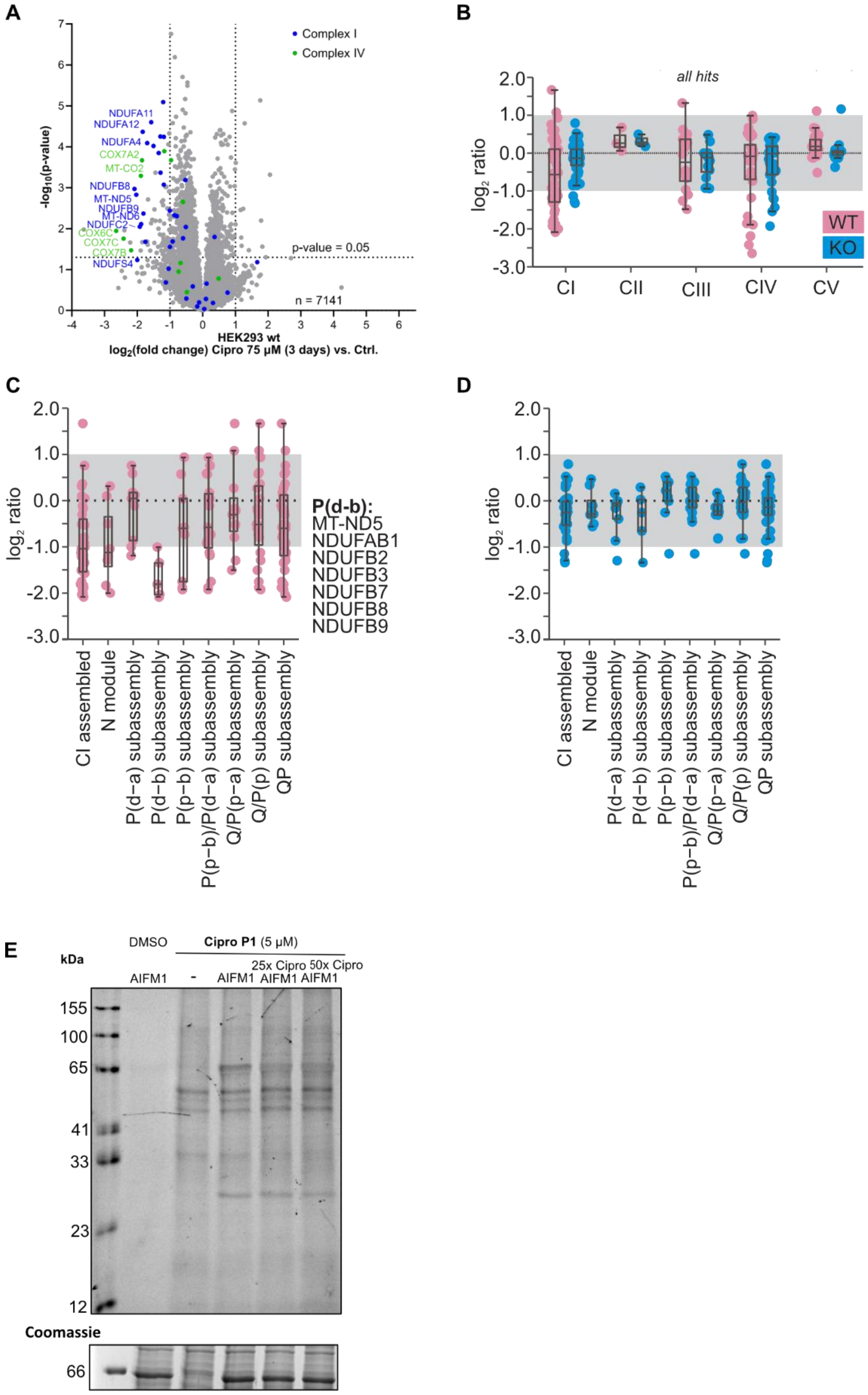

Figure S23: Target validation of AIFM1. (**A**) Volcano plot depicting the proteome regulation of the wild-type (WT) control HEK293 cells treated with Ciprofloxacin (75 µM) for 3 days versus vehicle control-treated WT cells featuring the distinct alteration of ETC complexes I and IV. Threshold lines represent a $\log_2$ regulation of ±1 and – $\log_{10}$ (P-value) of 1.3 (two-sided two-sample t-test, n = 5 replicates per group). The volcano plot of the wild-type (WT) control experiment is depicted in main figure 5B. (**B**) Comparison of respiratory chain complex protein levels in HEK293 WT or AIFM1 KO cells treated with ciprofloxacin or left untreated. Changes of protein levels of all identified subunits of the respective respiratory chain complexes upon ciprofloxacin treatment were plotted. In WT cells, levels of complex I and IV subunits decrease upon ciprofloxacin treatment. This effect is attenuated in AIFM1 KO

cells indicating that ciprofloxacin acts on AIFM1 (compare main Figure 5C for significant hits only). Comparison of complex I module levels in HEK293 WT or AIFM1 KO cells treated with ciprofloxacin or left untreated. Changes of protein levels of all identified subunits of complex I upon ciprofloxacin treatment were sorted according to the complex I module they are present in and plotted. In WT cells (**C**), the P(d-b) subassembly appears to be particularly affected upon ciprofloxacin treatment indicating a potential complex I assembly effect. This effect is attenuated in AIFM1 KO cells (**D**). Delays in complex I assembly upon ciprofloxacin treatment would be in line with a disturbed AIFM1 and mitochondrial IMS import function. Assembly intermediates taken from *Guerrero-Castillo* et *al*. 2017.[12] (**E**) Complete gel picture of analytical AfBPP of recombinant AIFM1 spiked into a human cell lysate background by **Cipro P1**. The labelling is partially outcompeted by Cipro, further validating AIFM1 engagement. The corresponding Coomassie-stain is depicted as loading control.

Figure S24

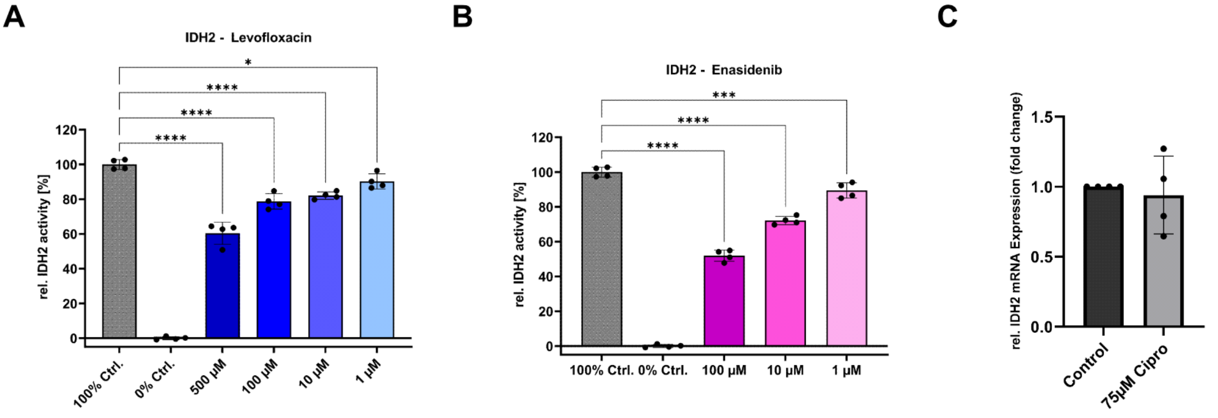

Figure S24: IDH2 *in vitro* activity assay in presence of Levofloxacin (**A**) and literature-known inhibitor Enasidenib (**B**). Partial, but concentration-dependent inhibition in a physiologically relevant range was detected. The mean and SD of 4 replicates per condition is plotted. The graphs are representative of three independent experiments. In the bar plots the statistical relevance based on one-way ANOVA with Dunnett´s multiple comparison test is depicted (ns meaning P-value > 0.05, * P-value ≤ 0.05, ** P-value ≤ 0.01, *** P-value ≤ 0.001, **** P-value ≤ 0.0001). (**C**) IDH2 mRNA levels of Ciprofloxacin (75 µM) treated PDL-hTERT cells are not significantly changed in comparison to vehicle control cells. GAPDH was used as housekeeper gene and fold change to control is plotted (n=4, mean and standard deviations are shown, wherein each dot represents an independent experiment).

Supplementary Information

Chemical proteomics reveal human off-targets of fluoroquinolone induced mitochondrial toxicity

Till Reinhardt[1], Yassmine El Harraoui[1], Alex Rothemann[2], Adrian T. Jauch[3], Sigrid Müller-Deubert[4], Martin F. Köllen[1], Timo Risch[5], Lianne H.C. Jacobs[2], Rolf Müller[5], Franziska R. Traube[6], Denitsa Docheva[4], Stefan Zahler[3], Jan Riemer[2], Nina C. Bach[1,*] and Stephan A. Sieber[1,*]

[1]Center for Functional Protein Assemblies, Department of Bioscience, TUM School of Natural Sciences, Technical University of Munich, Ernst-Otto-Fischer-Straße 8, 85748 Garching, Germany.

[2]Institute for Biochemistry and CECAD, University of Cologne, Cologne, Germany.

[3]Department of Pharmacy, Pharmaceutical Biology, Ludwig-Maximilians-Universität München, Butenandtstraße 5-13, 81377 Munich, Germany.

[4]Department of Musculoskeletal Tissue Regeneration, Orthopaedic Hospital König-Ludwig-Haus, University of Würzburg, 97076 Würzburg, Germany.

[5]Helmholtz Institute for Pharmaceutical Research Saarland (HIPS), Helmholtz Centre for Infection Research (HZI) and Saarland University Department of Pharmacy, Campus Building E8.1, 66123 Saarbrücken, Germany.

[6] Institut für Biochemie und Technische Biochemie, Universität Stuttgart, 70569 Stuttgart.

[*]Corresponding author(s).

Methods and Experimental Procedures

General Bio/Biochemistry

The fluoroquinolones (FQs) Ciprofloxacin (*Sigma-Aldrich, St. Louis, USA, Cat# 17850*) and Levofloxacin (*Alfa Aesar, Haverhill, USA, Cat# J66943*) stocks were prepared in sterile-filtered 0.1 N HCl to a maximum concentration of 50 mM. HCl was used as vehicle control, diluted in culture medium, and did not exceed 0.1% (v/v) in any experiment.

Human cell culture

Cell culture experiments were performed in HEK293, A549 or PDL-hTERT cells[1] cultured in DMEM high glucose medium supplemented with 10% FCS and 2 mM glutamine (all, *Sigma-Aldrich, St. Louis, USA, Cat# G7513*) in 5% CO_2 atmosphere at 37 °C in *Heracell 150i* incubators (*Thermo Fisher Scientific, Waltham, USA).* The human periodontal ligament (PDL-hTERT) cell line was provided by Prof. Denitsa Docheva. During standard cell passaging accutase (*Sigma-Aldrich)* or 2,5x trypsin/0.5% EDTA in PBS (*Capricorn Scientific*, *Ebsdorfergrund, Germany)* was used for cell detachment. Cell dishes in various sizes (10 cm, 15 cm) and flasks (T75, T175) were used for cultivation (*Sarstedt, Nümbrecht, Germany, Cat# 83.3902, 83.3903, 83.3911.002, 83.3912.002*). Sterile cell culture 6-well and 96-well plates were purchased from *Thermo Fisher Scientific, Waltham, USA, Cat# 130184, 130188*.

MTT assays

HEK or PDL-hTERT cells (1,5 x 10^3 cells/well) were seeded into 96-well plates and grown for 24 h. Subsequently, the medium was carefully removed by suction and 100 µL standard DMEM medium (10% FCS, 2 mM glutamine) with either vehicle control (1% (v/v), DMSO or sterile-filtered 0.1 N HCl) or a concentration range of the FQ compounds (1 % final DMSO or 0.1 N HCl concentration) was added. The cells were grown in presence of the compound and controls for 72 h and 20 µL thiazolyl blue tetrazolium bromide (MTT, 5 mg/mL in PBS, *Sigma-Aldrich, St. Louis, USA, Cat# M2128*) was added and incubated for 2 h at 37 °C, 5 % CO_2 to allow the MTT reagent to be metabolized. The medium was removed by suction and the formed formazan crystals dissolved in 200 µL DMSO. The read-out was performed with an *Infinite M200 Pro* microplate reader (*Tecan, Männedorf, Switzerland*) at 570 nm and background was subtracted at 630 nm. The absorbance values were normalized to DMSO/0.1 N HCl controls and the apparent IC_{50} value inferred and plotted in *GraphPad Prism* (Version 5.03) by regression. As in many cases no complete metabolic inhibition in the concentration range of the compounds was detected, approximate apparent IC_{50} values are indicated (e.g. > 400 µM). The experiment was performed with triplicates per condition in two independent experiments (n=2) for each compound.

FACS apoptosis cell assay

PDL-hTERT cells were seeded into 6-well plates and grown for 24 h. The medium was removed and fresh pre-warmed medium with FQ compound or vehicle control in the respective concentration was added and the cells incubated for 24 h. Subsequently, the medium was again removed and fresh medium with compound/ vehicle control added for 24 h. This step was repeated after 48 h of total compound inhibition and cells washed with PBS after a total compound incubation time of 72 h. As positive control, cells were treated with H_2O_2 (2 mM, 30 min, 37 °C). Cells were detached by accutase, centrifuged (500 ×g, 5 min), the accutase-containing medium removed and cells were resuspended in fresh medium and stored on ice until further processing. Next, cells were washed twice with PBS and subsequently counted. 200,000 cells per sample were transferred into a new tube. Apoptosis and necrosis were determined using FITC–Annexin V Apoptosis Detection Kit (*BioLegend, #422201*) and SYTOX Red Dead Cell Stain (*Life Technologies, #S34859*). To this end, cells were resuspended in 150 µl of Annexin V binding buffer supplemented with 0.75 µl of FITC-conjugated Annexin V and 0.15 µl of SYTOX Red Dead Cell Stain, gently vortexed, and incubated at RT for 15 min in the dark. Afterwards, samples were put on ice and the cell suspension was filtered through a 35-µm strainer before measurement. For the analysis, BD FACS Canto (recording of 10,000 events per sample) and FlowJo Single Cell Analysis Software (v10.8.0) were used. Gates (FSC [A] – SSC [A] to remove cell debris, FSC [A] – FSC [H] to gate for single cells, and last FITC/APC to distinguish between live, dead, and early apoptotic cells) were set once for the control sample and then applied to all other samples.

Live/dead staining and microscopy

For PDL-hTERT cells (9 x 104 cells /well) were plated in each well of 6-well plates (*Greiner Bio-One, Frickenhausen, Germany*) and incubated for 3 days at 37 °C and 5% CO2. Afterwards, cells were treated with 75 µM Cipro, as well as some wells were left untreated as controls. Fresh media (control or containing Cipro) were given on day 1 and 4. Afterwards, the cells were stained with 1 µM calcein acetoxymethyl (calcein AM) and 10 µM propidium iodide, diluted from 100 µM and 1 mM stock solutions respectively. The diluent was given to the cells in a mixture of 1:1 PBS to culture medium. Calcein AM is cell permeable and in living cells is converted to fluorescent calcein by intracellular estarases, thus labelling the cells in green colour. Propidium iodide is a fluorescent dye that can only traverse cells with damaged membranes, thus marking dead cells in red colour (to note: dead cells frequently detach during the staining procedure). Last, cells were imaged on day 1, 4, and 7 in phase-contrast, FITC (green) and Cy3 (red) fluorescent channels using Zeiss AxioVert.A1 microscope equipped with AxioCam ICc1 camera (*Carl Zeiss, Göttingen, Germany*). Each image was automatically processed using the “Min/Max” option provided by the micriscope software Zen 2.3 lite (*Carl Zeiss*). The

experiment was repeated three independent times (n = 3), each performed in duplicate wells for control and Cipro-containing groups.

Bacterial Cultivation

Bacteria of safety category S1 were cultivated in *Innova 40* or *Innova 44* incubators (*New Brunswick Scientific, Edison, USA*).

Bacterial strains and media

Table S1: Bacterial strains and their appropriate cultivation media.

Species	Strain	Medium for cultivation
Staphylococcus aureus	NCTC8325	B
Escherichia coli	MM28	LB
	RFM795	LB

Table S2: Composition of the media used for the cultivation of bacteria.

Media	Composition
B	10.0 g peptone ex casein<br>5.00 g NaCl<br>5.00 g yeast extract<br>in 1 L ddH2O, pH = 7.5
LB<br>(lysogeny broth)	10.0 g peptone ex casein<br>5.00 g NaCl<br>5.00 g yeast extract<br>1.00 g K2HPO4<br>in 1 L ddH2O, pH = 7.5

Overnight cultures

Overnight cultures were prepared using 5 mL of the respective medium inoculated with 5 µL of the desired bacterial cryostock (1:1,000) in a culture tube (*VWR, Radnor, USA, Cat# 60818-725*). The culture was incubated overnight (routinely 16 h) by shaking at 200 rpm at 37 °C. All overnight cultures were prepared freshly to avoid genetic variation. A sterile control was added each time (medium without bacteria).

Cryostocks

5 mL of an overnight culture of the desired bacteria were harvested by centrifugation (6,000 g, 10 min, 4 °C) and the supernatant was removed. The bacteria pellet was resuspended in 750 µL of fresh sterile medium. Sterile glycerol was added to reach a final concentration of 50%. The stock was mixed and stored at –80 °C in cryo tubes.

Minimal inhibitory concentrations (MIC) assay

MIC were determined with the broth dilution method. The overnight culture was diluted 1:10,000 in fresh medium and 99 µL aliquots were added to 96-well plates containing various concentrations of compounds (1 µL, 100× in DMSO or 0.1 N HCl) and the respective vehicle control (1 µL) as negative control in triplicates. After 20 h incubation at 200 rpm at 37 °C, the turbidity of the wells was analyzed.

Topoisomerase IV decatenation assay

The assay was performed as described before.[2] Briefly, *S. aureus* topoisomerase IV decatenation assay kit was purchased from *Inspiralis (Norwich, UK*, *Cat# SAD4001*) and conducted according to manufacturer conditions. Briefly, 1 U *S. aureus* topoisomerase IV was used to decatenate 200 ng kDNA by incubating in presence of controls or compound in provided assay buffer (10 mM Tris pH 7.5, 1 mM $MgCl_2$, 1 mM DTT, 0.3 mM ATP, 70 mM potassium glutamate, 0.01 mg/mL albumin) in 30 µL total volume for 30 min at 37 °C. The reactions were stopped by addition of 2.25 µL EDTA (250 mM) and mixed with appropriate amounts of agarose loading buffer. 10 µL aliquots were separated on a 0.8% agarose gel at 80 V for 1 h. Since an intercalator (*Gel-Red*, *Biotium, Fremont, USA, Cat# B-41003*) was present for visualization, decatenated products resolved in open circular and relaxed forms.

Proteomics experiments

Whole proteome analyses

Cell treatment and lysis

The respective cells were seeded into 10 cm dishes and incubated for 24 h at standard cell culture conditions (5% CO_2 atmosphere, 37 °C). The cells were treated with compound for the desired time in replicates per condition depending on the experiment as indicated in Table S3. For the whole proteome analysis of Ciprofloxacin-treated HEK293 cells over 14 days, the cells were passaged every 3 days using fresh medium and compound. For experiments monitoring the FQ-treatment in-depth after 3 days, fresh medium with compound was applied every 24 h to simulate daily drug application.

Table S3: Treatment conditions of human cell lines for the different whole proteome analysis.

<table>
<tr><th>Experiment</th><th>Compound</th><th>Concentration</th><th>Incubation times</th><th>Replicates per condition</th></tr>
<tr><td rowspan="3">FQ Proteome Analysis in HEK293</td><td rowspan="2">Ciprofloxacin</td><td>7.5 µM</td><td>3 h
3 days
7 days
14 days</td><td rowspan="3">3</td></tr>
<tr><td>75 µM</td><td>3 h
3 days
7 days
14 days</td></tr>
<tr><td>0.1 N HCl vehicle control</td><td>Same volume</td><td>14 days</td></tr>
<tr><td rowspan="2">FQ Proteome Analysis in HEK293 AIFM1 KO (prepared with SP3-protocol)</td><td>Ciprofloxacin</td><td>75 µM</td><td>3 days</td><td rowspan="2">5</td></tr>
<tr><td>0.1 N HCl vehicle control</td><td>Same volume</td><td>3 days</td></tr>
<tr><td rowspan="5">FQ Proteome Analysis in PDL-hTERT</td><td rowspan="2">Ciprofloxacin</td><td>7.5 µM</td><td rowspan="5">3 days</td><td rowspan="5">4</td></tr>
<tr><td>75 µM</td></tr>
<tr><td rowspan="2">Levofloxacin</td><td>7.5 µM</td></tr>
<tr><td>75 µM</td></tr>
<tr><td>0.1 N HCl vehicle control</td><td>Same volume</td></tr>
<tr><td rowspan="2">Onc201 Proteome Analysis in HEK293</td><td>Onc201</td><td>1 µM</td><td rowspan="2">24 h</td><td rowspan="2">4</td></tr>
<tr><td>DMSO vehicle control</td><td>Same volume</td></tr>
<tr><td rowspan="4">FQ Proteome Analysis in ClpP KO HEK293</td><td>Ciprofloxacin in wild-type HEK293</td><td>75 µM</td><td rowspan="4">3 days</td><td rowspan="4">4</td></tr>
<tr><td>Ciprofloxacin in ClpP KO HEK293</td><td>75 µM</td></tr>
<tr><td>0.1 N HCl vehicle control in wild-type HEK293</td><td>Same volume</td></tr>
<tr><td>0.1 N HCl vehicle control in ClpP KO HEK293</td><td>Same volume</td></tr>
</table>

Cells were lysed at the respective time-points by removing the medium from the plates, washing the cells with cold PBS once and addition of 300 µL pre-cooled lysis buffer (50 mM Tris pH 7.5, 150 mM NaCl, 1% (v/v) NP40, 0.1% (w/v) sodium deoxycholate, 1 mM EDTA) supplemented with 1 tablet of protease inhibitor (*cOmplete Mini EDTA-free, Roche, Basel, Switzerland, Cat.# 04693159001*) per 10 mL lysis buffer directly into the dishes. After incubation on ice for 15 min, the lysate was collected, snap-frozen and stored at –80 °C until further processing. The lysate was cleared by centrifugation (12,000 ×g, 15 min, 4 °C), the total protein concentration measured using *RotiQuant universal* bicinchoninic acid assay (BCA) assay (*Carl Roth, Karlsruhe, Germany, Cat# 0120.*1) with BSA (*Sigma-Aldrich, St. Louis, USA, Cat# P5619*) calibration curve according to manufacturer instructions.

Equal protein amounts per sample (typically 100-300 µg) were precipitated using 4× excess volume of cold acetone (–20 °C) overnight at –80 °C. The precipitated proteins were pelletized (12,000 ×g, 10 min, 4 °C), and the supernatant was disposed. The proteins were washed three times with 1 mL cold methanol (–80 °C). Resuspension was achieved by sonication (10 s, 10% intensity, pulse 5) using a

Bandelin SONOPULS equipped with *Bandelin UW2070* and *MS73* lance (*Bandelin electronic, Berlin Germany*) and the proteins were pelletized *via* centrifugation (12,000 ×g, 20 min, 4 °C). The protein pellet was resuspended in UA buffer (100 µL, 8 M urea in 100 mM Tris pH 8.5) and subjected to the filter aided sample preparation (FASP protocol).[3] Briefly, 30 kDa cut-off *Micron* centrifugal filters (*Merck, Darmstadt, Germany, Cat# Millipore MRCFC0R030*) were washed once with NaOH (0.05 N, 500 µL) by centrifugation (12,000 ×g, 15 min, r.t.) and subsequently equilibrated with 500 µL UA buffer. The dissolved protein pellets were reduced with 1 mM DTT (1 µL of 100 mM DTT in H_2O to 100 µL sample) for 1 h at 37 °C, 500 rpm in thermoshaker and applied to the filters. After centrifugation (12,000 ×g, 15 min, r.t.), the samples on the filters were washed with 200 µL UA buffer, centrifuged (12,000 ×g, 15 min, r.t.) and the flow-through discarded. Alkylation was performed by addition of 100 µL IAA solution (0.05 M iodoacetamide in UA buffer) per filter and incubation for 20 min at r.t. without mixing. The filter units were centrifuged (12,000 ×g, 15 min, r.t.), washed twice with 100 µL UA buffer (12,000 ×g, 15 min, r.t., each) and subsequently twice with 100 µL ABC buffer (0.05 M NH_4HCO_3 in H_2O). Trypsin (enzyme to protein amount ratio 1:100) in 40 µL ABC buffer was added to the filters and protein digest performed in a wet chamber at 37 °C, 200 rpm over for 16 h. The filter units were transferred into new 1.5 mL low-bind Eppendorf tubes and centrifuged (12,000 ×g, 10 min, r.t.). The samples were acidified with formic acid to pH < 6 and desalted by stage-tipping. Stage-tips were prepared with two *Empore Supelco* C18 discs (*Merck, Darmstadt, Germany, Cat# 66883-U*) and washed once with 50 µL of MeOH, once with 200 µL of 80% (v/v) acetonitrile and 0.5 % (v/v) FA in H_2O and once with 200 µL of 0.5% (v/v) FA in H_2O by centrifugation (1-2 min, 500 ×g) without running the stage-tips dry. The samples were loaded on the tips and washed twice with 200 µL of 0.5% (v/v) FA in H_2O by centrifugation (1-2 min, 500 ×g) without running the stage-tips dry. Desalted peptides were eluted consecutively by 60 µL and 30 µL of 80% (v/v) acetonitrile and 0.5 % (v/v) FA in H_2O. The eluates were lyophilized and for MS measurement the samples were dissolved in 1% (v/v) FA in H_2O by pipetting up and down, vortexing and sonication for 2× 5 min (brief centrifugation after each step). *Millipore* centrifugal filter units (*Durapore PVDT 0.22 µm, Merck, Darmstadt, Germany, Cat# UFC30GVNB*) were equilibrated with 300 µL 1% FA (13,000 ×g, 2 min, room temperature) and samples were filtered through the equilibrated filters (centrifugation: 13,000 ×g, 1 min, room temperature). The samples were subjected to LC-MS/MS measurement (see LC-MS/MS measurement section).

The samples of FQ Proteome Analysis in HEK293 AIFM1 KO were prepared following the SP3 protocol as previously published[4] with minor changes. After lysis all samples were adjusted to a total protein amount of 25 µg in a final volume of 50 µL and transferred to a 96-well plate (Polypropylene, V-bottom, *Greiner* cat. 651201). For protein alkylation, a 1:2 mixture of TCEP and IAA of each 500 mM was prepared, and 3 µL was added to each sample. After incubation for 15 min at 950 rpm and room

temperature, the excess of IAA was quenched by the addition of 2 µL 500 mM DTT per sample. To each sample, 10 µl of a 1:1 mix of washed (3x with H_2O) hydrophobic and hydrophilic carboxylate-coated magnetic beads (*Cytiva*, cat#65152105050250 and 45152105050250) was added, followed by the addition of 150 µL EtOH to precipitate the proteins onto the beads. The subsequent steps were carried out using an automated liquid handling system (*Hamilton Microlab Prep*, *Hamilton*). The plate was then incubated for 5 min at 500 rpm and room temperature, following washing of the beads. Each washing step was performed with the help of a 96-well ring magnet (*Alpaqua*, *Magnum FLX*) by placing the plate on it and slowly (20 µL/s) removing the supernatant. Washing involved removing the plate from the magnet, adding the respective washing solution, and shaking for 1 min at 800 rpm and room temperature. The samples were washed three times with 180 µL of 80% EtOH and once with 180 µL acetonitrile. Protein digestion was performed with 0.45 µL trypsin (0.5 µg/µL, sequencing grade, *Promega*) in 100 µL of 50 mM TEAB overnight at 800 rpm and 37 °C in a plate shaker with a heated lid. The plate was tightly sealed during digestion. The next day, peptides were eluted from the beads in the liquid handling system with 50 µL of 3% FA and transferred into new wells for desalting. Desalting was performed using pre-equilibrated stage tips containing two layers of styrenedivinylbenzene-reverse phase sulfonate (SDB-RPS) disks (Empore, 3M) The stage tips were equilibrated with 150 µL wash buffer 1 (1% (v/v) TFA in isopropanol) before loading the samples. Samples were loaded for 10 min at 500 x g, followed by washing with 150 µL wash buffer 1 for 10 min at 800 x g, and another wash with 150 µL wash buffer 2 (0.2% (v/v) TFA in H2O). Peptides were eluted with 50 µL elution buffer (1% (v/v) ammonia, 80% (v/v) acetonitrile) for 5 min at 300 x g, followed by 5 min at 800 x g. Eluted peptide samples were dried using a SpeedVac (*Concentrator Plus, Eppendorf, Hamburg, Germany*), following reconstitution in 150 µL of 1% (v/v) FA by pipetting up and down, vortexing and sonication for 2× 5 min (brief centrifugation after each step). 2 µL of each sample was subjected for LC-MS/MS measurements on a timsTOF Pro mass spectrometer (Bruker) in data-independent acquisition (DIA) mode (see LC-MS/MS measurement section).

Affinity-based protein profiling (AfBPP)

Analytical gel-based AfBPP

Analytical gel-based AfBPP was performed as described before with modifications for human cell lines.[2] Respective cells were seeded into 6-well plates and grown until 80-90% confluency. The cells were washed with pre-warmed sterile PBS (1 mL), 1 µL probe (1:100 stock in DMSO) or DMSO as vehicle control in 1 mL DMEM medium (without FCS, except in +/- FCS comparison experiment) added and the cells incubated in standard conditions (5% CO_2 atmosphere, 37 °C) for 1 h. None-UV-irradiation controls were lysed at this point by medium removal, washing of the cells with pre-cooled PBS (1 mL) and addition of 100 µL pre-cooled lysis buffer (50 mM Tris pH 7.5, 150 mM NaCl, 1% (v/v) NP40, 0.1%

(w/v) sodium deoxycholate, 1 mM EDTA) supplemented with 1 tablet of protease inhibitor (*cOmplete Mini EDTA-free, Roche, Basel, Switzerland, Cat# 04693159001*) per 10 mL lysis buffer directly into the dishes and incubation on ice for 15 min. Subsequently, all other samples were irradiated on cool packs at 365 nm for **P1** probes (*TL-D BLB 18 W, Philips, Amsterdam, The Netherlands*) or 300 nm for **P2** probes (*LZC-UVB 8 W, Luzchem, Ottawa, Canada*) for 10 min. Lysis was performed as before for non-irradiated controls. The lysate was cleared by centrifugation (12,000 ×g, 15 min, 4 °C), the total protein concentration measured using *RotiQuant universal* bicinchoninic acid assay (BCA) assay (*Carl Roth, Karlsruhe, Germany, Cat# 0120.*1) with BSA (*Sigma-Aldrich, St. Louis, USA, Cat# P5619*) calibration curve according to manufacturer instructions. Equal protein amounts per sample (typically 100-300 µg) were adjusted to 90 µL and 8 µL CuAAC master mix (1 µL TAMRA-azide (10 mM in DMSO, *Baseclick, Neuried, Germany, Cat# BCFA-008-10*), 5 µL BTTAA (10 mM in H_2O, *Jena Bioscience, Jena, Germany, CLK-067*), 2 µL $CuSO_4$ (50 mM in H_2O)) added. The click reaction was started by addition of 2 µL sodium ascorbate (100 mM in H_2O) and the reaction incubated in the dark for 1 h. Subsequently, the reaction was stopped by precipitation of the proteins by 500 µL cold acetone (–20 °C, MS grade) for 2 h to overnight at –20 °C. The precipitated proteins were pelletized (12,000 ×g, 10 min, 4 °C), and the supernatant was disposed. The proteins were resuspended in 100 µL PBS and 100 µL 2× SDS loading buffer and the samples heated to 95 °C for 10 min. After SDS-PAGE (7.5 % gel) applying 40 µL of each sample, utilizing *BenchMark* fluorescent protein standard marker (*Invitrogen, Thermo Fisher Scientific, Waltham, USA, Cat# LC5928*) and *Roti-Mark* standard protein marker (*Carl Roth, Karlsruhe, Germany, Cat# T851.1*) or *PeqGold* protein marker I (*VWR, Radnor, USA, Cat# 27-1010 I*), fluorescent bands were visualized by a *Las-3000* fluorescence darkbox (*Fujifilm, Minato, Japan*) with a *Fujinon* VRF 43LMD Lens, 605DF40 filter and 520 nm EPI excitation wavelength. Subsequently, total protein bands were visualized by Coomassie staining as loading controls.

Preparative MS-based AfBPP

Preparative MS-based AfBPP was performed as described before with modifications for human cell lines.[2] Respective cells were seeded into 15 cm dishes and grown until 80-90% confluency. The cells were washed with pre-warmed sterile PBS (10 mL), 10 µL probe (1:100 stock in DMSO) or DMSO as vehicle control in 10 mL pre-warmed DMEM medium (without FCS) added and the cells incubated in standard conditions (5% CO_2 atmosphere, 37 °C) for 1 h. Dishes were irradiated on cool packs at 365 nm for **P1** probes (*TL-D BLB 18 W, Philips, Amsterdam, The Netherlands*) or 300 nm for **P2** probes (*LZC-UVB 8 W, Luzchem, Ottawa, Canada*) for 10 min and the medium removed subsequently. The cells were washed with 10 mL pre-cooled PBS and lysis was performed by addition of 1 mL pre-cooled lysis buffer (50 mM Tris pH 7.5, 150 mM NaCl, 1% (v/v) NP40, 0.1% (w/v) sodium deoxycholate, 1 mM EDTA) supplemented with 1 tablet of protease inhibitor (*cOmplete Mini EDTA-free, Roche, Basel, Switzerland,*

Cat# 04693159001) per 10 mL lysis buffer directly into the dishes and incubation on ice for 15 min. The lysate was transferred into 1.5 mL Eppendorf tubes and optionally snap-frozen and stored at –80 °C until further processing. The lysate was cleared by centrifugation (12,000 ×g, 15 min, 4 °C), the total protein concentration measured using *RotiQuant universal* bicinchoninic acid assay (BCA) assay (*Carl Roth, Karlsruhe, Germany, Cat# 0120.*1) with BSA (*Sigma-Aldrich, St. Louis, USA, Cat# P5619*) calibration curve according to manufacturer instructions and samples adjusted to 430 µL (0.5 mg/mL protein concentration). Subsequently, 10 µL 10% (w/w) SDS in PBS (c_{final} = 0.4 %) and 40 µL CuAAC master mix (5 µL Biotin-PEG3-N3 (10 mM in DMSO, *Sigma-Aldrich, St. Louis, USA, Cat# 76024*), 25 µL BTTAA (10 mM in H_2O, *Jena Bioscience, Jena, Germany, CLK-067*), 10 µL $CuSO_4$ (50 mM in H_2O)) was added, the click reaction started by addition of 10 µL sodium ascorbate (100 mM in H_2O) and incubated in the dark for 1 h. Proteins were precipitated by addition of 4× excess volume (2 mL) cold acetone (–20 °C) and incubation overnight at –20 °C. The precipitated proteins were pelletized (16,900 ×g, 15 min, 4 °C), and the supernatant was disposed. The Protein pellet was washed two times with 1 mL cold methanol (–80 °C). Resuspension was achieved by sonication (10 s, 10% intensity) and proteins were pelletized *via* centrifugation (10,000 ×g, 10 min, 4 °C). After the washing steps, the supernatant was disposed and the pellet resuspended in 500 µL 0.4% (w/v) SDS in PBS at room temperature by sonication (10 s, 10% intensity). 50 µL avidin-agarose beads (*Sigma-Aldrich, St. Louis, USA, Cat# A9207*) per sample were prepared by washing three times with 1 mL 0.4% (w/v) SDS in PBS. All centrifugation steps were conducted at 400 ×g for 3 min at room temperature. The 50 µL washed avidin-agarose beads were added to each sample and incubated under continuous inverting (1 h, room temperature). Beads were washed three times with 1 mL 0.4% (w/v) SDS in PBS, two times with 6 M urea in ddH_2O and three times with 1 mL PBS. The beads were resuspended in 200 µL denaturation buffer (7 M urea, 2 M thiourea in 20 mM pH 7.5 HEPES buffer). For reduction, Tris(2-carboxyethyl)phosphine (TCEP, 500 mM, 2 µL, *Alfa Aesar, Haverhill, USA, Cat# J60316*) was added, the tubes were mixed by vortexing shortly and incubated in a thermoshaker (600 rpm, 1 h, room temperature). Then 2-iodoacetamide (IAA, 500 mM, 4 µL, *Sigma-Aldrich, St. Louis, USA, Cat# I6125*) was added for alkylation, the tubes were mixed by vortexing shortly and incubated in a thermoshaker (600 rpm, 30 min, room temperature, in the dark). Remaining IAA was quenched by the addition of dithiothreitol (DTT, 500 mM, 4 µL, *Sigma-Aldrich, St. Louis, USA, Cat# 43819*). The tubes were shortly mixed by vortexing and incubated in a thermoshaker (600 rpm, 30 min, room temperature). LysC (0.5 µg/µL, *Fujifilm Wako Pure Chemicals, Minato, Japan, Cat# 125-05061*) was thawed on ice and 1 µL was added to each microcentrifuge tube, the tubes were shortly mixed by vortexing and incubated in a thermoshaker (600 rpm, 2 h, room temperature, in the dark). Triethylammonium bicarbonate (TEAB) solution (600 µL, 50 mM in water) and then trypsin (1.5 µL, 0.5 µg/µL in 50 mM acetic acid, *Promega, Madison, USA, Cat# VC551C*) were added to each tube with a short vortexing step after each addition. The microcentrifuge tubes were

incubated in a thermoshaker (600 rpm, 16 h, 37 °C). The digest was stopped by adding 10 µL formic acid (FA) and vortexing followed by centrifugation (12,000 ×g, 3 min, room temperature). 50 mg *SepPak* C18 columns (*Waters, Milford, USA, Cat# WAT054960*) were equilibrated by gravity flow two times with 1 mL acetonitrile and three times with 1 mL aqueous 0.1% trifluoroacetic acid (TFA) solution. Subsequently, the samples were loaded by gravity flow, washed three times with 1 mL aqueous 0.1% TFA solution and once with 0.5 mL aqueous 0.5% formic acid (FA) solution. Elution of peptides into new 2.0 mL Protein LoBind *Eppendorf* tubes was performed by two times addition of 250 µL elution buffer (80% acetonitrile, 0.5% FA) by gravity flow followed by 250 µL elution buffer by vacuum flow until all liquid was eluted from the column. The eluates were lyophilized. Before MS measurement the samples were dissolved in 30 µL 1% FA by pipetting up and down, vortexing and sonication for 15 min (brief centrifugation after each step). *Millipore* centrifugal filter units (*Durapore PVDT 0.22 µm, Merck, Darmstadt, Germany, Cat# UFC30GVNB*) were equilibrated with 300 µL 1% FA (13,000 g, 2 min, room temperature) and samples were filtered through the equilibrated filters (centrifugation: 12,000 ×g, 1 min, room temperature).

Thermal protein profiling (TPP)

The experiment was performed similarly to TPP-TR experiments described by Franken et al. with minor modifications.[5] HEK293 cells were grown in 8 T175 flasks to approx. 80-90% confluency. Per replicate two T175 flasks were treated with Ciprofloxacin (75 µM, 1 h, 37 °C) and two T175 flasks with the same volume of 0.1 N HCl as vehicle control. Per condition two replicates were used, leading to a total of 8 T175 flasks. The cells were washed with PBS, detached with accutase, the two flask per replicate condition combined and the cells pelletized (500 ×g, 3 min, r.t.). The medium was removed and each pellet (Cipro #1, Cipro #2, Ctrl. #1, Ctrl #2) washed twice with cold PBS. Each pellet was resuspended in 1.2 mL cold PBS supplemented with either 75 µM Ciprofloxacin or the same volume of 0.1 N HCl, depending on the sample. The cell suspension of each sample was distributed into 10 PCR tubes (100 µL per tube), resulting in 10 stripes á 4 tubes (2×2 conditions, 10 temperature points). The PCR tubes were subjected to the temperature gradient using a PCR cycler. Due to temperature gradient limitations, two separate programs had to be run to cover the desired temperature range (see **Table S4**). The PCR tubes were heat-treated according to the two programs for 3 min and directly snap frozen in liquid nitrogen. The samples were stored at –80 °C until further processing.

Table S4: PCR cycle program for heat-treatment of TPP samples.

**Program 1**<br>T = 43.5 °C<br>Gradient = 13.5 °C	Position	1	5	8	11		
	Temp. [°C]	37.2	41.2	45.6	49.3		
**Program 2**<br>T = 60 °C<br>Gradient = 17 °C	Position	1	3	5	7	9	12
	Temp. [°C]	52.6	54.5	57.8	60.9	64.1	67.5

The cells were thawed in a heating block set to 25 °C and directly snap-frozen again. This freeze-thaw cycle was repeated twice. 110 µL of the samples were transferred into ultracentrifugation tubes (*Beckman Coulter, Brea, USA*) and the original PCR tubes rinsed with 50 µL PBS and also transferred to the ultracentrifugation tubes. The samples were ultra-centrifuged (100,000 ×g, 20 min, 4 °C, *Beckman Coulter*, *Brea, USA*) and 100 µL of each supernatant transferred to low-bind Eppendorf tubes. Of the first two lowest concentrations additional 10 µL of supernatant were taken for protein quantification using *RotiQuant universal* bicinchoninic acid assay (BCA) assay (*Carl Roth, Karlsruhe, Germany, Cat# 0120.*1) with BSA (*Sigma-Aldrich, St. Louis, USA, Cat# P5619*) calibration curve according to manufacturer instructions. The average of the two temperature conditions was taken to adjust each sample accordingly to 60 µg total protein and precipitation was performed by addition of 4× excess of cold acetone and incubation at −20 °C overnight. The proteins were pelletized by centrifugation (21,000 ×g, 20 min, 4 °C) and washed once with 500 µL cold methanol (21,000 ×g, 20 min, 4 °C). The proteins were resuspended in 200 µL buffer X (7 M urea, 2 M thiourea in 20 mM HEPES pH 7.5) and 0.4 µL DTT (500 mM in H_2O) added and incubated for 45 min at room temperature in a thermoshaker (1000 rpm). Subsequently, alkylation was performed by addition of 2 µL iodoacetamide (IAA, 550 mM in 50 mM TEAB buffer) and the samples incubated at room temperature, 1000 rpm for 30 min. The alkylation was quenched by addition of 1.6 µL DTT (500 mM in H_2O) and incubation for 30 min at room temperature in a thermoshaker (1000 rpm). Proteins were pre-digested with 1 µL LysC (0.5 µg/µL, *Fujifilm Wako Pure Chemicals*, *Minato, Japan, Cat# 125-05061*) for 2 h at room temperature (450 rpm). Subsequently, 600 µL of 50 mM TEAB buffer was added and 1.5 µL trypsin (0.5 µg/µL in 50 mM acetic acid, *Promega, Madison, USA, Cat.# VC551C*) added and incubated at 37 °C, 1000 rpm for 16 h. The digest was stopped by adding 8 µL formic acid (FA) and vortexing followed by centrifugation (12,000 ×g, 3 min, room temperature). 50 mg *SepPak* C18 columns (*Waters, Milford, USA, Cat# WAT054960*) were equilibrated by gravity flow two times with 1 mL acetonitrile and three times with 1 mL aqueous 0.1% trifluoroacetic acid (TFA) solution. Subsequently, the samples were loaded by gravity flow, washed three times with 1 mL aqueous 0.1% TFA solution and once with 0.5 mL aqueous 0.5% formic acid (FA) solution. Elution of peptides into new 1.5 mL Protein LoBind *Eppendorf* tubes was performed by two

times addition of 200 µL elution buffer (80% acetonitrile, 0.5% FA) by gravity flow followed by 200 µL elution buffer by vacuum flow until all liquid was eluted from the column. The eluates were concentrated to dryness in a SpeedVac (*Concentrator plus, Eppendorf, Hamburg, Germany*). The peptides were reconstituted in 7.5 µL TMT labeling buffer (50 mM HEPES, 20 % acetonitrile, pH 8.5) in a sonication bath (*VWR, Radnor, USA*) for 2× 5 min. After brief centrifugation, 5 µL of each aliquoted TMT10plex label (= 50 µg, *Thermo Fisher Scientific, Waltham, USA,* Cat# *90110*) was added to the respective samples (labelling each temperature point with one individual TMT channel). And incubated for 1h at room temperature (400 rpm). The labelling reaction was quenched by addition of 1 µL hydroxylamine solution (5% (v/v) in H_2O) and 188.5 µL 0.1% (v/v) FA was added. 1 µL of each temperature point was combined and desalted *via* stage tipping to perform a test LC-MS/MS measurement to analyze TMT-labeling efficiency. The samples were stored at −80 °C until further processing. All 10 temperature-point samples of one replicate and compound-condition were pooled (resulting in 4 samples again: Cipro #1, Cipro #2, Ctrl. #1, Ctrl. #2) and concentrated to dryness in a SpeedVac (*Concentrator plus, Eppendorf, Hamburg, Germany*). After reconstitution in 500 µL 0.5% (v/v) FA, the samples were desalted using the 50 mg *SepPak* C18 columns (*Waters, Milford, USA, Cat# WAT054960*) as described above. After concentration to dryness, the samples were reconstituted in 110 µL HILIC buffer A (95% (v/v) acetonitrile, 5% (v/v) H_2O, 0.1 % (v/v) FA) and sonicated (10 s, 10 % intensity, pulse 5) using a *Bandelin SONOPULS* equipped with *Bandelin UW2070* and *MS73* lance (*Bandelin electronic, Berlin Germany*). After centrifugation (21,000 ×g, 10 min, 4 °C) 105 µL were transferred into a HPLC vial and each of the 4 samples separately offline-fractionated using a HILIC column (*YMC-Pack PVA-Sil* column, 5 µm, 150 x 2.1 mm, 120 Å, *YMC Europe GmbH, Dinslaken, Germany*) equipped to an *Ultimate 3000* HPLC system (*Dionex, Sunnyvale, USA*) using buffer A (95% MeCN, 5% H*2*O, 0.1% TFA) and buffer B (95% H*2*O, 5% MeCN, 0.1% TFA) in following gradient at a flow rate of 0.2 mL/min: 7.5 min 0% buffer B, 50 min to 30% buffer B, 3.5 min to 50% buffer B, 2.5 min to 100% buffer B, total gradient length 62.5 min. The fractions were pooled into 10 samples per replicate and condition (resulting in 4× 10 samples) and concentrated to dryness. After reconsitition in 30 µL 0.1% (v/v) FA, vortexing, sonication for 2× 5 min (brief centrifugation after each step) and filtering through *Millipore* centrifugal filter units (*Durapore PVDT 0.22 µm, Merck, Darmstadt, Germany, Cat# UFC30GVNB*) equilibrated with 300 µL 1% FA (13,000 g, 2 min, room temperature), the samples were subjected to LC-MS/MS measurement.

LC-MS/MS measurement

Samples were measured either on a Orbitrap Q Exactive Plus (*Thermo Fisher Scientific, Waltham, USA*), LTQ Orbitrap Fusion (*Thermo Fisher Scientific, Waltham, USA*) or a Tims-TOF Pro (*Bruker, Billerica, USA*) instrument (see **Table S5**).

Table S5: LC-MS/MS instrument usage in proteomic experiments.

**Experiment**	**Experiment type**	**Instrument**
FQ Proteome Analysis in HEK293	Whole proteome analysis	Q Exactive Plus
FQ Proteome Analysis in HEK293 AIFM1 KO	Whole proteome analysis	TimsTOF Pro (DIA mode)
FQ Proteome Analysis in PDL	Whole proteome analysis	TimsTOF Pro (DDA mode)
FQ AfBPP in HEK293	Affinity-based protein profiling	Q Exactive Plus
FQ AfBPP in A549	Affinity-based protein profiling	Q Exactive Plus
FQ AfBPP in PDL	Affinity-based protein profiling	TimsTOF Pro (DDA mode)
FQ TPP in HEK293	Thermal protein profiling	Orbitrap Fusion
Onc201 Proteome Analysis in HEK293	Whole proteome analysis	Orbitrap Fusion
FQ Proteome Analysis in ClpP KO HEK293	Whole proteome analysis	Orbitrap Fusion

LC-MS/MS measurement methods:

Q Exactive Plus

Samples were analyzed with an *UltiMate 3000 nano* HPLC system (*Dionex, Sunnyvale, USA*) using *Acclaim C18 PepMap100* 75µm ID x 2 cm trap and *Acclaim PepMap RSLC C18* (75 µm ID x 50 cm) separation columns (*Thermo Fisher Scientific, Waltham, USA*) in an EASY-spray setting coupled to a *Qexactive Plus* (*Thermo Fisher Scientific, Waltham, USA*). Peptide samples were loaded on the trap column and washed with 0.1% TFA, then transferred to the separation column (buffer A: H2O with 0.1% FA, buffer B: MeCN with 0.1% FA, flow 0.3 µL/min, gradient: to 5% buffer B in 7 min, from 5% to 22% buffer B in 105 min, then to 32% buffer B in 10 min, to 90% buffer B in 10 min and hold at 90% buffer B for 10 min, then to 5% buffer B in 0.1min and hold 5% buffer B for 9.9 min) and ionized at spray voltage of 2.0 kV and a capillary temperature of 275°C. The *Q Exactive Plus* was operated in a TOP12 data dependent mode with full scan acquisition in the orbitrap at a resolution of R = 140,000 and an AGC target of $3e^6$ in a scan range of 300-1500 m/z with a maximum injection time of 80 ms. Monoisotopic precursor selection as well as dynamic exclusion (dynamic exclusion duration: 60 s) was enabled. Precursors with charge states >1 and intensities greater than $1e^4$ were selected for fragmentation. Isolation was performed in the quadrupole using a window of 1.6 m/z. Precursors were analyzed in a scan range of 200–2000 m/z to an AGC target of $1e^5$ and a maximum injection time of 100 ms. Peptide fragments were generated by higher-energy collisional dissociation (HCD) with a normalized collision energy of 27% and detected in the orbitrap.

LTQ Orbitrap Fusion for whole proteome analysis

Samples were analyzed *via* HPLC-MS/MS using an *UltiMate 3000 nano* HPLC system (*Dionex, Sunnyvale, USA*) equipped with an *Acclaim* C18 PepMap100 75 µm ID x 2 cm trap and an *Acclaim* C18 PepMap RSLC, 75 µM ID x 15 cm separation columns (*Thermo Fisher Scientific, Waltham, USA*) coupled

to a *LTQ Orbitrap Fusion* (*Thermo Fisher Scientific, Waltham, USA*). Samples were loaded on the trap and washed for 10 min with 0.1% FA (at 5 µL/min), then transferred to the separation column and separated using a, 112 min gradient from 4% to 35% MeCN followed by 4 min at 80% MeCN in 0.1% FA (at 200 nL/min flow rate). The *LTQ Orbitrap Fusion* was operated in a 3 second top speed data dependent mode. The full scan acquisition was performed in the orbitrap at a resolution of 120,000 and an ion target of $4e^5$ in a scan range of 300 –1700 m/z. The monoisotopic precursor selection as well as the dynamic exclusion for 60 sec were enabled. Precursors with charge states of 2–7 and intensities greater than $5e^3$ were selected for fragmentation. Isolation was performed in the quadrupole using a window of 1.6 m/z. Precursors were collected to a target of $1e^2$ for a maximum injection time of 250 ms with "inject ions for all available parallelizable time" enabled. Fragments were generated using higher-energy collisional dissociation (HCD) and detected in the ion trap at a rapid scan rate. Internal calibration was performed using the ion signal of fluoranthene cations (EASY-ETD/IC source).

LTQ Orbitrap Fusion for thermal protein profiling (TPP)

TMT-labelled peptides of the TPP samples were analyzed on an *UltiMate 3000* nano HPLC system (*Dionex*, *Sunnyvale, USA*) equipped with an *Acclaim C18 PepMap100* (75 µm ID × 2 cm) trap column (*Thermo Fisher Scientific, Waltham, USA*) and an *Aurora* (25 cm × 75 µm ID, 1.6 µm FSC C18, *IonOpticks, Fitzroy, Australia*) separation column in a column oven (*Sonation*, *Biberach, Germany*) heated to 40 °C coupled to an *Orbitrap Fusion* (*Thermo Fisher Scientific, Waltham, USA*) in EASY-spray setting. Samples were loaded on the trap column and washed for 10 min with 0.1% (v/v) TFA in H2O at a flow rate of 5 µL/min. Subsequently, peptides were transferred to the separation column and online separated using a 120 min gradient (Buffer A: H2O + 0.1% (v/v) FA; Buffer B: Acetonitrile + 0.1% (v/v) FA) with a flow rate of 300 nL/min. The gradient was as follows: in 10 min to 5% buffer B, in 50 min from 5% to 22% buffer B and in 60 min from 22% to 35% buffer B. Subsequently, a column washing step using 90% buffer B for 10 min and column re-equilibration with 5% buffer B for 5 min was performed. MS1 full scans were recorded at a resolution of 120.000 in the Orbitrap, parameters set as follows: Ion transfer tube temperature 275 °C, RF lens amplitude 60%, 375-1500 m/z scan range, automatic gain control (AGC) target of 4×10^5, 3 s cycle time and 50 ms maximal injection time. The monoisotopic precursor selection as well as the dynamic exclusion for 60 sec were enabled. Peptides with a higher intensity than 5.0×10^4 and charge states between 2 and 7 were selected for fragmentation in the higher-collisional induced dissociation (HCD) cell at 35% collision energy and analyzed in the Orbitrap, the isolation window was set to 1 m/z, the resolution to 60000 and an AGC target of 5×10^4 and a maximal injection time of 118 ms.

TimsTOF Pro

Peptides were measured and online-separated using an *UltiMate 3000 nano* HPLC system (*Dionex, Sunnyvale, USA*) coupled to a *timsTOF Pro* (*Bruker, Billerica, USA*) mass spectrometer *via* a *CaptiveSpray* nano-electrospray ion source (*Bruker, Billerica, USA*) and *Sonation* (*Biberach, Germany*) column oven. Peptides were first loaded on the trap column (*Acclaim PepMap 100 C18*, 75 µm ID x 2 cm, 3 µm particle size, *Thermo Fisher Scientific, Waltham, USA*), washed with 0.1% formic acid in water for 7 min at 5 µL/min and subsequently transferred to the separation column (*Aurora* C18 column, 25 cm × 75 µm, 1.7 µm, *IonOpticks, Fitzroy, Australia*) and separated over a 60 min gradient from 5% to 28% B, then to 40 % B over 13 min, followed by 10 min at 95% before re-equilibration and at a flow rate of 400 nL/min. The mobile phases A and B were 0.1 % (v/v) formic acid in water and 0.1% (v/v) formic acid in acetonitrile, respectively.

The *timsTOF Pro* was operated in data-dependent PASEF mode with the dual TIMS analyzer operating at equal accumulation and ramp times of 100 ms each with a set 1/K0 ion mobility range from 0.85 to 1.40 V × s × cm^{-2}. The capillary voltage of the *CaptiveSpray* source was set to 1500 V. 10 PASEF scans per topN acquisition cycle were performed, resulting in a total cycle time of 1.17 s. The mass range was set from 100 to 1700 m/z. Only precursors reaching an intensity threshold of 1750 arbitrary units were considered for fragmentation, precursors reaching a target intensity of 14500 arbitrary units were dynamically excluded for 0.4 min. The quadrupole isolation width was set to 2 m/z for m/z < 700 and to 3 m/z for m/z > 800. The collision energy was ramped linearly as a function of the mobility from 59 eV at 1/K0 = 1.6 V × s × cm^{-2} to 20 eV at 1/K0 = 0.6 V × s × cm^{-2}. TIMS elution voltages were calibrated linearly to obtain the reduced ion mobility coefficients (1/K0) using three *Agilent* (*Santa Clara, USA*) ESI-L Tuning Mix ions (m/z 622, 922 and 1,222) spiked on the *CaptiveSpray* Source inlet filter.

For the whole proteome analysis in HEK293 AIFM1 KO cells the *timsTOF Pro* was operated in data-independent dia-PASEF mode with the dual TIMS analyzer operating at equal accumulation and ramp times of 100 ms each with a set 1/K0 ion mobility range from 0.60 to 1.60 V × s × cm^{-2}. for MS1 scans. The dia-PASEF settings for fragmentation were set to a mass range of 400 to 1201 m/z and an ion mobility range of 0.60 to 1.43 V × s × cm^{-2}. Two ion mobility isolation windows were performed per dia-PASEF scan with 26 m/z window widths. A total of 32 isolation windows with 1 m/z overlaps to cover the mass range were used resulting in 16 dia-PASEF scans per MS1 scan and an estimated total cycle time of 1.80 s (see table S6). The collision energy was ramped linearly as a function of the mobility from 59 eV at 1/K0 = 1.3 V × s × cm^{-2} to 20 eV at 1/K0 = 0.85 V × s × cm^{-2}. TIMS elution voltages were calibrated linearly to obtain the reduced ion mobility coefficients (1/K0) using three Agilent ESI-L Tuning Mix ions (m/z 622, 922 and 1,222) spiked on the CaptiveSpray Source inlet filter.

Table S6: DiaPASEF – long gradient method: isolation window layout in detail.

MS Type	Scan	Start IM [1/K0]	End IM [1/K0]	Start Mass [m/z]	End Mass [m/z]
MS1	0	0.6	1.6	100	1700
dia-PASEF	1	0.9	1.2	800	826
dia-PASEF	1	0.6	0.9	400	426
dia-PASEF	2	0.92	1.22	825	851
dia-PASEF	2	0.62	0.92	425	451
dia-PASEF	3	0.93	1.23	850	876
dia-PASEF	3	0.63	0.93	450	476
dia-PASEF	4	0.95	1.25	875	901
dia-PASEF	4	0.65	0.95	475	501
dia-PASEF	5	0.96	1.26	900	926
dia-PASEF	5	0.66	0.96	500	526
dia-PASEF	6	0.98	1.28	925	951
dia-PASEF	6	0.68	0.98	525	551
dia-PASEF	7	0.99	1.29	950	976
dia-PASEF	7	0.69	0.99	550	576
dia-PASEF	8	1.01	1.31	975	1001
dia-PASEF	8	0.71	1.01	575	601
dia-PASEF	9	1.02	1.32	1000	1026
dia-PASEF	9	0.72	1.02	600	626
dia-PASEF	10	1.04	1.34	1025	1051
dia-PASEF	10	0.74	1.04	625	651
dia-PASEF	11	1.06	1.36	1050	1076
dia-PASEF	11	0.76	1.06	650	676
dia-PASEF	12	1.07	1.37	1075	1101
dia-PASEF	12	0.77	1.07	675	701
dia-PASEF	13	1.09	1.39	1100	1126
dia-PASEF	13	0.79	1.09	700	726
dia-PASEF	14	1.1	1.4	1125	1151
dia-PASEF	14	0.8	1.1	725	751
dia-PASEF	15	1.12	1.42	1150	1176
dia-PASEF	15	0.82	1.12	750	776
dia-PASEF	16	1.13	1.43	1175	1201
dia-PASEF	16	0.83	1.13	775	801

Proteomics data analysis

Whole proteome analysis

Proteomic data analysis was performed similarly as described before.[2] Peptide and protein identifications were performed using *MaxQuant* 1.6.17.0 software with *Andromeda* as search engine using following parameters: Cysteine carbamidomethylation was set as fixed modification and methionine oxidation and N-terminal acetylation as variable modifications, trypsin (without N-terminal cleavage to proline) as the proteolytic enzyme with max. of 2 missed cleavages. Label-free quantification (LFQ) mode was performed with a minimum ratio count of 2. Searches were performed with the Uniprot database for *H. sapiens* (reviewed, Uniprot entry UP000005640, organism ID 9606, downloaded 12.09.2021). The "match between runs" (0.7 min match and 20 min alignment time window) and second peptide identification options were activated. For *TimsTOF* data the TOF MS/MS match tolerance was set to 0.05 Da. All other parameters were used as pre-set in the software.

Proteomic data analysis of the whole proteome analysis in HEK293 AIFM1 KO cells measured in data-independent dia-PASEF mode were performed using *DIA-NN* version 1.8.1 with "FASTA digest for library-free search" and "deep learning-based spectra, RTs and IMs prediction".[6] "Trypsin/P" with a maximum of two missed cleavages was set as protease. The maximum number of variable modifications was set to 0 and N-terminal methionine excision and cysteine carbamidomethylation selected as fixed modifications. Peptide precursors with a length of 7 to 30 amino acids and a charge range from 2 to 4 were considered. The precursor m/z range was set from 300 to 1800 m/z, the fragment ion ranges from 200 to 1800 m/z. "Generate spectral library" and quantities matrices were activated and a precusor FDR of 1% was used. Mass accuracy, MS1 accuracy and scan window was set to automatic (0), "use isotopologues", match between runs (MBR) and "remove likely interferences" were activated. The neural network classifier was used in "single-pass mode", proteins were inferred from genes and the quantification strategy set to "Robust LC (high precision)". For cross-run normalisation "RT-dependent" was selected and "Smart profiling" used for library generation. The additional option "--relaxed-prot-inf" was used. The protein group quantity matrix was used for further downstream analysis in Perseus.Statistical analysis was performed with *Perseus* 1.6.14.0. LFQ intensities were log2(x) transformed. Putative contaminants, reverse peptides and peptides only identified by site were deleted. Valid values were filtered for at least 3 valid values in each group and a missing values imputation was performed over the total matrix (width 0.3, downshift 1.8). A two-sample student`s t-test with permutation-based FDR (0.05) and the significance thresholds were set at p-value = 0.05 (-log10(p-value) = 1.3) and an enrichment factor of 2 (log2(x) = 1) as indicated in the volcano plots.

Affinity-based protein profiling

MS raw files were processed with *MaxQuant* version 2.0.3.0 as described above. Statistical analysis was performed as described above using *Perseus* version 1.6.15.0, with the difference that filtering of valid values was performed with "three in at least one group". The significance thresholds after the t-test were set at p-value = 0.05 (-log10(p-value) = 1.3) and an enrichment factor of 4 (log2(x) = 2) as indicated in the volcano plots.

Thermal protein profiling (TPP)

MS raw data was analyzed using *MaxQuant* software (version 1.6.5.0) and peptides were searched against Uniprot database for Homo sapiens (taxon identifier: 9606, downloaded on 15.07.2019, canonical). Fractions were assigned for each experiment (10 fractions á 4 experiments). Group specific parameters were set to "Reporter ion MS2" with 10plex TMT isobaric labels for N-terminal and lysine modification selected. Carbamidomethylation of cysteines was set as fixed modification and oxidation of methionines and acetylation of N-termini were set as variable modifications. Trypsin was set as digestion enzyme, a maximum of 2 missed cleavages sites allowed. For main search, precursor mass tolerance was set to 4.5 ppm and fragment mass tolerance to 0.5 Da. The isotope correction factor was set for each TMT channel according to the data sheet of the TMT labels. The option "Second peptide identification" was enabled, and the false discovery rate (FDR) determination carried out by applying a decoy database and thresholds were set to 1% FDR at peptide-spectrum match and at protein levels. Furthermore, the "match between runs" (0.7 min match and 20 min alignment time windows) option was enabled. The remaining parameters were used as default settings. Calculated corrected reporter ion intensities were normalized to the channel corresponding to the lowest temperature and were used to determine the melting curves of the proteins and the resulting thermal shifts (T_m). These were calculated using R (version 4.1.1) and the TPP package (version 3.20.1) using the "analyzeTPPTR" function.[5] Proteins that fulfilled all requirements were considered to have a significant thermal shift. For visualization of the TPP output files, the data was filtered as follows: R^2 > 0.8 for all fitted curves, plateaus < 0.3 for HCl control curves, steepest slopes of melting curves < -0.06, difference in T_m between both HCl control replicates < 1.5 °C. The data was further visualized using *Perseus* version 1.6.13.0 and *GraphPad Prism 9.*

Pathway analysis

Ingenuity Pathway Analysis (IPA) of the proteomics data was done with QIAGEN IPA (QIAGEN Inc., https://digitalinsights.qiagen.com/IPA)[7]. For IPA, proteomics data were first processed as described in section *Proteomics data analysis*. p-values and log2 fold changes from the student's t-tests were used

for pathway analysis. In IPA, core analysis was performed with the setting "human" as species and a p-value cutoff of 0.05. Analysis was focused on "Pathways".

ClpP target validation efforts

hClpP expression and purification

Human ClpP was expressed and purified as described previously.[8] Briefly, an expression construct of human ClpP (Uniprot ID Q16740) covering residues 57-228[9] fused to a C-terminal Strep-II-tag in a pET301 plasmid was expressed in *E. coli* Rosetta 2 (DE3) cells at 25 °C for 20 h after induction with IPTG (500 µM). Following harvest and cell lysis by sonication in Strep binding/wash buffer (100 mM Tris HCl, 150 mM NaCl, 1 mM EDTA, pH 8.0), the protein was purified by affinity chromatography (*StrepTrap HP 5 mL, Cytiva, Marlborough, USA*; Step elution buffer: 100 mM Tris HCl, 150 mM NaCl, 1 mM EDTA; 2.5 mM desthiobiotin, pH 8.0) and subsequent size exclusion chromatography (*Superdex 200*, *Cytiva, Marlborough, USA*; Aloisius buffer: 20 mM HEPES, 100 mM NaCl, pH 7.0) using an *Äkta Purifier* FPLC system (*GE Healthcare, Chicago, USA*).

ClpP Peptidase Assay

The residual hClpP peptidase activity was measured upon treatment with compounds by monitoring the cleavage of the optimized fluorogenic substrate acetylalanylhomoarginyl-2-aminooctanoyl-7-amino-4-carbamoylmethylcoumarin (Ac-Ala-hArg-2-Aoc-ACC, custom-synthesis by *Bachem, Bubendorf, Switzerland*) as described previously.[8] In a black 96-well plate, 1 µL inhibitor or respective vehicle control (usually DMSO, except for ciprofloxacin 0.1 N HCl) was aliquoted in four replicates in three different concentrations (100 × stocks, final concentrations in assay: 1 µM, 10 µM, 100 µM). To exclude for vehicle effects, the respective other solvents were also added if necessary to achieve the same compositions in each condition. Subsequently, 98 µL enzyme buffer mix (final concentration of hClpP 0.4 µM in assay buffer 50 mM HEPES, 300 mM KCl, 1 mM DTT, 15% (v/v) glycerol, pH = 7.5) was added to the wells and incubated at 37 °C for 15 min. Kinetic measurement (1 min intervals) was started after adding 1 µL of peptide substrate Ac-Ala-hArg-2-Aoc-ACC (10 mM stock in DMSO, final concentration 100 µM). Fluorescence of the cleaved dye was detected for 120 min at 37 °C using an *Infinite M200 pro* (*Tecan, Männedorf, Switzerland,* excitation: 380 nm; emission: 430 nm, manual gain: 100) plate reader. The slope of the fluorescence over time signal was calculated via linear regression using *GraphPad Prism* (version 5.03), the first 5 min were usually disregarded to allow for thermal equilibration effects. The residual activity of inhibitor treated protein was determined in comparison to vehicle-treated control samples, which were normalized to 100% activity. The experiments were repeated in at least two independent experiments.

ClpXP Protease Assay (GFPssrA-degradation assay)

For ClpXP protease activity assays human ClpP (expression & purification see above) and *E. coli* ClpX (kindly provided by *Thomas Gronauer*)[10] were used and the experiments conducted as described before with minor modifications.[8,10] As recombinant *H. sapiens* ClpX is instable and *E. coli* ClpX recognizes peptides C-terminally tagged with a short amino acid sequence and forms a functional complex with *H. sapiens* ClpP, it is suitable as a *H. Sapiens* ClpX substitute for *in vitro* protease activity assays.[10] Measurements were carried out in a total reaction volume of 60 µL in black, flat-bottom 96-well plates and the residual protease activity determined by monitoring the cleavage of ssrA-tagged GFP by the decrease of fluorescence signal. First, 0.6 µL inhibitor or respective vehicle control (usually DMSO, except for ciprofloxacin 0.1 N HCl) was aliquoted in four replicates in three different concentrations (100 × stocks, final concentrations in assay: 1 µM, 10 µM, 100 µM). To exclude vehicle effects, the respective other solvents were also added to the master mix if necessary to achieve the same compositions in each condition. The master mixes were prepared with hClpP14 (0.1 µM), *E. coli* $ClpX_6$ (0.2 µM) in PZ buffer (25 mM HEPES, 200 mM KCl, 5 mM $MgCl_2$, 1 mM DTT, 10 % (v/v) glycerol, pH 7.6) supplemented with 1 × ATP regeneration mix (10 × stock, 25 mM HEPES, 200 mM KCl, 5 mM $MgCl_2$, 1 mM DTT, 40 mM ATP, 160 mM creatine phosphate, 200 U/mL creatine kinase, 10 % (v/v) glycerol, pH 7.6) and added to the wells. In the negative control no ClpP and ClpX was added to the master mix. After incubation at 37 °C for 15 min, the reactions were started by the addition of GFP-SsrA substrate to a final concentration of 0.6 µM and fluorescence was detected for 3 hours at 37 °C using an *Infinite M200 pro* (*Tecan, Männedorf, Switzerland,* excitation: 485 nm; emission: 535 nm, manual gain: 60) plate reader. The initial slopes of the fluorescence decays were calculated via linear regression using GraphPad Prism (version 5.03). The residual activity of compound treated protein was determined in comparison to vehicle-treated control samples, which were normalized to 100% activity. The experiments were repeated in at least two independent experiments.

ClpP wt vs ClpP KO full proteome Analysis

For whole proteome analysis, HEK293T wild-type cells and HEK293T hClpP knockout cells (kindly provided by *Prof. Aleksandra Trifunovic, CECAD Cologne*)[11] were used and the experiment performed as described in the proteomics section.

IDH2 activity assay

Recombinant human isocitrate dehydrogenase was purchased from *active motif* (*Carlsbad, USA, Cat# 31615*). The literature known IDH2-inhibitor Enasidenib was purchased from *Cayman Chemical* (*Ann Arbor, USA, Cat# 21277*). Activity assays were performed as previously described with minor modifications.[12] Briefly, the catalytic activity of converting isocitrate to 2-oxogluterate (α-ketoglutarate) converting the cofactor $NADP^+$ to NADPH was measured by a NADPH-coupled assay.

The NADPH-dependent diaphorase (*Sigma-Aldrich, St. Louis, USA, Cat# SLBT8928*) converted resazurin (*ACROS Organics*, *Thermo Fisher Scientific, Waltham, USA,* Cat# 189900010) to fluorescent resorufin for activity read-out. To IDH2 (2 nM) in assay buffer (150 mM NaCl, 50 mM K_2HPO_4, 10 mM $MnCl_2$, 10 % (v/v) glycerol, 0.03 % (w/w) BSA, pH 7.0) was added $NADP^+$ cofactor (c_{end}= 44 µM) and β-mercaptoethanol (c_{end}= 2.5 mM) for a total reaction volume of 79 µL per well (black 96-well plate, 4 replicates per condition). Conditions lacking IDH2 were added as control. After addition of 1 µL of compound (100 × stock, final concentrations 500 µM, 100 µM, 10 µM and 1 µM) and/or the respective vehicle controls, the mixture was incubated at 25 °C for 1 h in the dark. Subsequently, the reaction was initiated by addition of 20 µL of substrate mix (0.2 mM isocitrate, 60 µg/mL diaphorase, 200 µM resazurin in assay buffer). Fluorescence was continuously measured using an *Infinite M200 pro* (*Tecan, Männedorf, Switzerland*, excitation: 544 nm, emission: 590 nm, manual gain: 80). The initial slopes of the fluorescence increases were calculated *via* linear regression using *GraphPad Prism* (version 9). The residual activity of compound treated protein was determined in comparison to vehicle-treated control samples and IDH2-lacking control samples which were normalized to 100% and 0% activity, respectively. The experiments were repeated in at least three independent experiments.

Total RNA isolation, cDNA synthesis and quantitative PCR analysis of IDH2

PDL-hTERT cells (2.5 x 10^5 cells / flask) were plated into T-25 flasks and cultured at 37°C and 5% CO2. Cells were supplemented with 75 µM Cipro or were left untreated. Fresh media (control or containing Cipro) were given on day 1 and 4. RNA was harvested from the cells on day 4. For RNA isolation, the GF-1 Total RNA Isolation Kit (*Vivantis, Darul Ehsan, Malaysia*) was used according to the manufacturer's instructions. The RNA was eluted in 50 µl RNAase free water and the RNA amount was measured using the DS-11+ Spectrophotometer (*Denovix, Wilmington, Delaware, USA*). Until further use the RNA was stored at -80 °C. Copy DNA (cDNA) was synthesised by implementing the M-MLV Reverse Transcriptase kit from *Promega* (*Madison, Wisconsin, USA*) and using 1 µg RNA from each sample. The obtained cDNA samples were stored at -20°C until further use. A PCR master mix (19 µl per sample) was prepared with 7 µl nuclease free water, 10 µl 2x KAPA SYBR FAST qPCR Mix (*Promega*), 2 µl primer mix (1µl of 10 pM forward and 1 µL of 10 pM reverse primer) and 1 µl of the cDNA sample. Primer pairs for IDH2 and the housekeeper gene GAPDH were purchased from *Qiagen* (*Hilden, Germany*; primer sequences are property of the manufacturers). The PCR reaction was carried out in the CFX Duet Real-Time qPCR System (*Bio-Rad, Feldkirschen, Germany*), equipped with Bio-Rad CFX Maestro software (both, *Bio-Rad, Feldkirschen, Germany*). The qPCR program was set to 95°C for 3 minutes, and then 39 cycles of 94°C for 10 seconds, 55 °C for 10 seconds, and 72°C for 10 seconds. Quantitative PCR results were analysed using the 2 -$\Delta\Delta C_T$ method and fold change of Cipro—treated

versus control cells was depicted in bar plots (mean and standard deviation). The RNA isolation, cDNA synthesis and qPCR were repeated 4 independent times (n=4).

Mitochondrial NADPH level determination

Isolation of intact mitochondria was performed similar to the *abcam* "subcellular fractionation protocol". PDL-1 cells were seeded in 10 cm petri dishes at a density of 2.0 million cells/dish and grown overnight. On the next day, cells were stimulated for 6 h or 72 h with 7.5 µM or 75 µM Ciprofloxacin or 0.1 N HCl, respectively. In case of the 72 h stimulation, every 24 h the medium was removed and fresh medium (including the Ciprofloxacin or the vehicle control) was added. Cells were scraped off with a rubber policeman in fractionation buffer, the cell suspension passed ten-times through a 27 G needle and the mitochondrial fraction obtained by differential centrifugation. In brief, the nuclear fraction was removed by centrifuging at 720 xg for 5 min. The mitochondria containing supernatant was centrifuged at 10,000 ×g for 5 min to obtain the mitochondrial pellet. To increase the purity, the pellet was resuspended in fractionation buffer by passing it ten-times through a 25 G needle and re-centrifugation at 10,000 ×g for 10 min. The mitochondria fraction was resuspended in PBS, protein determination performed using Bradford assay, and all samples set to the same concentration with PBS. The *Promega* NADP/NADPH-Glo^{TM} assay kit (*Promega, Walldorf, Germany, Cat# G9081*) was used to measure NADPH levels individually according to the manufacture's instruction in triplicates per condition. NADP/NADPH-Glo^{TM} detection reagent was added to the processed mitochondrial lysates and after 2h incubation luminescence was recorded (*Orion II microplate luminometer, Titertek Berthold, Bad Wildbad, Germany*). The assays were performed in at least three independent experiments.

AIFM1 target validation

Generation of AIFM1 knockout cells

The generation and characterization of AIFM1 knockout HEK293T cells was described previously.[13] In brief, AIFM1 knockout HEK293T cell lines were generated using the pSpCas9(BB)-2A-GFP (PX458) CRISPR/Cas9 construct[14] (a gift from *F. Zhang*; *Addgene*, plasmid 4813). CRISPR/Cas9 gRNAs were designed for gene disruption using CHOPCHOP software.[15] Transfections were performed using Lipofectamine LTX (*Thermo Fisher Scientific*) and green fluorescent cells were individually sorted. Genomic DNA was isolated from each cell line and the targeted region amplified. PCR products were ligated into pGEM4Z expression vectors and single clones were analyzed by *Sanger* sequencing. Multiple single clones were analysed for loss of AIFM1 and complex I on protein level.

AIFM1 expression & purification

Recombinant AIFM1 was expressed in a Rosetta2 *E. coli* strain. Bacterial growth was conducted in LB media supplemented with riboflavin and FAD shaking at 37°C and 180 rpm. Expression was induced with 1.0 mM IPTG and incubated for further 16 h before harvesting. Cells were harvested on ice in PBS and cell pellets stored at -20°C. Cells were lysed by French press and the 6xHis-tagged constructs were purified by Immobilized Metal Affinity Chromatography using Ni Sepharose (6 Fast Flow, GE). The centrifugation-cleared bacterial lysate was bound to beads in binding buffer (200 mM NaCl, 20 mM Tris/Cl pH = 7.4). Beads were washed with binding buffer supplemented with 20 mM imidazole prior to elution with 150 mM imidazole. Imidazole was removed using PD-10 columns (Cytiva) and the proteins stored at 4 °C.

Recombinant Labelling AIFM1

For gel-based fluorescence labelling of recombinant AIFM1, 1.7 µM recombinant protein was spiked-in to PBS or PBS-diluted HEK293 cell lysate (100 µg per reaction sample). The total reaction volume per condition was 100 µL using Eppendorf tubes. After addition of Cipro P1 (5 µM) the samples were incubated at room temperature for 30 min. For competition conditions samples were incubated with Ciprofloxacin for 30 min at room temperature prior to probe addition. Subsequently, the samples were irradiated on ice for 5 min with UV light (365 nm, *TL-D BLB 18 W, Philips, Amsterdam, The Netherlands)*. Click Master-mix (8 µL, 100 µM TAMRA-azide, *Baseclick, Neuried, Germany, Cat# BCFA-008-10*, 500 µM BTTAA ligand, *Jena Bioscience, Jena, Germany, CLK-067*, 1 mM $CuSO_4$) was added and the CuAAC reaction started by addition of 2 µL of aq. sodium ascorbate (c_{end}= 2 mM). The reaction was incubated in the dark at room temperature for 1 h and subsequently 4× excess volume of acetone was added to precipitate proteins at −20 °C overnight. After pelletizing the proteins (max. rpm, 10 min, 4 °C) SDS loading buffer (150 µL) was added, the samples denatured at 95 °C for 10 min and subjected to SDS-PAGE. Fluorescence scanning was performed using a *Las-3000* fluorescence darkbox (*Fujifilm, Minato, Japan*) with a *Fujinon* VRF 43LMD Lens, 605DF40 filter and 520 nm EPI excitation wavelength and loading controls were developed with Coomassie stain afterwards.

PTGR2

PTGR2 (*Antikoerper-online, Aachen, Germany, Cat# ABIN1098255*) activity was measured as described before with minor modifications by *Wiebke Hutwelker* at the *BMWZ* of the *Leibniz University Hannover*.[16,17] Briefly, Ciprofloxacin 1 µL (1:50 stock in 0.1 N HCl) was added to 44 µL recombinant PTGR2 in buffer (200 nM, Tris Buffer, 1 mM EDTA, 50 mM TCEP, 300 µM NADPH) and incubated at 37 °C for 20 min. Subsequently, 5 µL of substrate solution (20 µM, 15-ketoprostaglandin, *Cayman Chemical*, *Ann Arbor, USA, Cat# 14720*) was added and incubated for further 30 min at 37 °C. The

reaction was terminated by addition of 800 µL EtOAc+0.5% AcOH, 300 µL H2O and 100 µL internal standard (13,14-dihydro-15-ketoprostaglandin-d4, 300 pmol/sample, *Cayman Chemical*, *Ann Arbor, USA, Cat# 10010606*). Of the organic phase 600 µL was transferred into a fresh Eppendorf tube and the solvent removed in a SpeedVac. The residue was dissolved in 100 µL acetonitrile/ H2O (1:1) and measured with LC-MS/MS. Gradient: Solvent A = H2O + 0.1% formic acid, solvent B = acetonitrile + 0.5% formic acid, flow rate = 0.4 mL/min, run length 6 min, gradient = 0 min 30% B, 1 min 30% B, 2 min 31.5% B, 2.1 min 95% B, 4 min 95% B, 4.1 min 30% B, 6 min 30% B. MS settings: Capillary = 3.0 kV, sampling cone = 20.0 kV, extraction cone 4.0 kV, source temperature = 100 °C, desolvation temperature = 350 °C, cone gas flow = 50.0 L/h, desolvation gas flow = 650.0 L/h, scan time = 0.250 s, inter-scan time = 0.020 s, mass range = 100-800 Da. Masses of 15-ketoprostaglandin (349.2 Da, collision energy = 21.0 eV), 13,14-dihydro-15-ketoprostaglandin (351.2 Da, collision energy = 18.0 eV) and 13,14-dihydro-15-ketoprostaglandin (355.2 Da, collision energy = 10.0 eV) were set for MRM. The areas under the curve (AUC) were used to quantify 15-keto-PGE2 and 13,14-dihydro-15-keto-PGE2 levels by determining peak areas in relation to internal standard PGE2-d4. The experiment was performed once.

SCARB1

Microscale thermophoresis (MST) was performed as follows: Monolith His-Tag Labeling Kit RED-tris-NTA 2nd Generation (*NanoTemper Technologies, Munich, Germany, Cat# MO-L018*) was used for KD determination as recommended by the purchaser. Briefly, SCARB1 (*Hölzel Biotech, Cologne, Germany, Cat# SC1-H82E5*) concentration (50 nM) was adjusted in assay buffer and labelled by the RED-tris-NTA dye (100 nM). This mixture was added to compound dilution series (c = 1 mM to 30 nM) and measured using *Monolith NT.115* (*NanoTemper Technologies, Munich, Germany*). The experiment was performed once and analysed with the MO Affinity Analysis software (*NanoTemper*).

NUDT1 target validation efforts:

Recombinant NUDT1 expression & purification

Expression and purification of recombinant NUDT1 was done as in procedures described previously with minor modifications.[18,19] Full length NUDT1 gene synthesis (see insert sequence, not codon-optimized) and cloning into pET28a(+) using restriction sites NdeI and XhoI was ordered from *TWIST Bioscience* (*South San Francisco, USA*). Insert sequence:

GGCGCTAGCCGCTTGTATACGTTAGTGCTGGTTCTGCAGCCACAAAGAGTTTTGCTTGGAATGAAAAAACGCG
GATTTGGCGCAGGACGCTGGAATGGCTTCGGCGGGAAAGTTCAGGAAGGTGAAACCATCGAAGATGGCGCG
CGGCGTGAACTTCAGGAAGAGTCTGGCTTGACGGTTGACGCGTTGCATAAAGTAGGTCAAATAGTTTTTGAAT
TTGTGGGGGAGCCCGAACTGATGGATGTTCATGTGTTTTGTACTGACAGTATACAAGGCACACCCGTGGAATC
TGATGAAATGCGTCCGTGTTGGTTTCAGTTGGACCAGATACCGTTCAAAGATATGTGGCCTGACGATAGCTACT

GGTTTCCACTGTTACTGCAGAAAAAAAAGTTTCATGGTTACTTTAAATTTCAGGGCCAAGATACCATTCTGGAT
TACACTTTACGTGAGGTGGACACCGTCTGA

The plasmid was transformed into chemically competent *E. coli* BL21 (DE3) and expression clones verified by sequencing. An overnight culture of the expression strain (LB + 1:1000 kanamycin) was grown and 1 L flasks (LB medium + 1:1000 kanamycin) were inoculated 1:100 and incubated at 37 °C, 200 rpm until OD_{600} = 0.5 – 0.6. The flasks were cooled on ice for 15 min, the overexpression induced by addition of IPTG (500 µM) and incubated at 20 °C, 200 rpm for 20 h. The bacteria were harvested, the pellet of 2 L culture combined, washed with cold PBS and stored at –80 °C. The pellet was resuspended in 30 mL Buffer A (10 mM Tris pH 7.4, 500 mM NaCl, 1 mM DTT, 10 mM imidazole) and lysis was performed by sonification (7 min 30% intensity, 3 min 70%, 7 min 30%, 3 min 70%, pulse 5, *Bandelin SONOPULS* equipped with *Bandelin UW2070* and *MS73* lance, *Bandelin electronic, Berlin Germany*). The lysate was cleared by centrifugation (40,000 ×g, 4 °C, 30 min) and filtered through a *Whatman* filter (0.45 µm PVDF w/GMF, *Cytiva, Marlborough, USA, Cat# 6818-1304*). Nickel-affinity chromatography purification was performed with *Äkta Pure* system (*GE Healthcare, Chicago, USA*) using an in buffer A equilibrated HisTrap HP (5 mL, *Cytiva, Marlborough, USA, Cat# 17-5248*) column. The filtered lysate was applied *via* the sample pump and subsequently the column was washed for 4.9 column volumes (CV) with buffer A, 6.9 CV with wash buffer 1 (10 mM Tris pH = 7.4, 1 M NaCl, 1 mM DTT, 10 mM imidazole) and 6.9 CV wash buffer 2 (10 mM Tris pH = 7.4, 500 mM NaCl, 1 mM DTT, 40 mM imidazole). Bound protein was eluted with elution buffer (10 mM Tris pH = 7.4, 500 mM NaCl, 1 mM DTT, 500 mM imidazole for 7 CV and NUDT1 containing fraction identified *via* intact-protein MS (IPMS). The fractions were pooled and dialyzed in buffer (10 mM Tris pH = 7.4, 500 mM NaCl) with simultaneous thrombin cleavage by addition of 200 µL thrombin (200 U, 1 U/µL reconstituted in H_2O*, Sigma-Aldrich, St. Louis, USA, Cat# T4648*) into the dialysis membrane (*Carl Roth, Karlsruhe, Germany, Cat# E659.1*) overnight at 4 °C. Successful thrombin cleavage was verified by IPMS. Reverse nickel-affinity purification (same conditions as before), but this time collecting the non-bound flow-through, was performed to purify thrombin-cleaved protein. The fractions containing the cleaved protein were desalted on the *Äkta Pure* system (*HiTrap HP Desalting 5 mL*, *Cytiva, Marlborough, USA, Cat# 17-1408*, elution with 1.5 CV buffer 1 = 20 mM Tris, pH 7.5, 1 mM DTT) and subjected to anion exchange chromatography (*Resource Q 1 mL*, *GE Healthcare, Chicago, USA, Cat# 17-1177-01*) for further purification. The desalted sample was applied by sample pump, washed with buffer 1 for 5 CV and eluted with a linear gradient to 100% buffer 2 (20 mM Tris pH 7.5, 1 mM DTT, 1 M NaCl) over 20 CV. Fractions containing non-aggregated and active NUDT1 were concentrated using a 3 kDa MWCO Amicon (*Ultra-15, Merck Millipore, Darmstadt, Germany, Cat# UFC9003*) to a concentration of 750 µM and snap-frozen in liquid nitrogen in aliquots to be stored at –80 °C. The identity of the recombinant protein was verified by IPMS (calc. 18,232.8 Da, found 18,232.5 Da) and SDS-PAGE (approx. 18 kDa).

NUDT1 Activity Assay

The activity assay was performed as described before with minor modifications.[20] Briefly, the conversion of dGTP (*New England Biolabs, Ipswich, USA, Cat# N0442S*) to dGMP and inorganic pyrophosphatate (pp_i) by NUDT1 was monitored. The generation of pp_i was detected by conversion with inorganic pyrophosphatase (*E. coli*, *New England Biolabs, Ipswich, USA, Cat#* M0361S) to inorganic phosphate (p_i) forming a green complex with malachite green and acidic ammonium molybdate. The known NUDT1 inhibitor (*S*)-crizotinib (*Sigma-Aldrich, St. Louis, USA, Cat# PZ0240*) was used as positive control. The FQs Ciprofloxacin and Levofloxacin were tested in the concentrations 500 µM, 100 µM, 10 µM and 1 µM; (*S*)-crizotinib in 10 µM, 1 µM and 0.1 µM. In transparent 96-well plates 0.4 µL of compounds or respective vehicle controls (1:100 stocks, FQs in 0.1 N HCl, (*S*)-crizotinib in DMSO) were added in triplicates into the wells and 39 µL of enzyme master mix (50 nM NUDT1, 0.2 U/mL pyrophosphatase in assay buffer = 50 mM Tris pH 7.5, 100 mM NaCl, 10 mM $MgCl_2$ and 1 mM DTT) was added. Conditions with master mix lacking NUDT1 were added as control. The plate was incubated for 30 min at r.t., and the enzymatic reaction was started by addition of 0.4 µL dGTP (10 mM in H_2O) to each well and the plate was further incubated for 30 min at room temperature. The malachite green reagent was obtained by mixing 1 volume of 4.2% (w/v) ammonium molybdate in 4 N HCl with 3 volumes of 0.045% (w/v) malachite green and addition of 0.01% (v/v) Tween20 (*Carl Roth, Karlsruhe, Germany, Cat# 9005-64-5*) directly before use. The mixture was filtered through 0.2 µm PTFE syringe filters (25 mm*, fisherbrand, Thermo Fisher Scientific, Waltham, USA, Cat# 15141499*) and 160 µL added to each well, after 5 min the absorbance at 630 nm was quantified using an *Infinite M200 Pro* (*Tecan, Männedorf, Switzerland*) plate reader. The residual activity of compound-treated protein was determined using *GraphPad Prism* (version 9) in comparison to vehicle-treated control and NUDT1-lacking control samples, which were normalized to 100% and 0% activity, respectively. The experiments were repeated in at least three independent experiments.

Recombinant NUDT1 labelling

For gel-based fluorescence labelling of recombinant NUDT1, 1 µM recombinant protein was spiked-in to PBS or PBS-diluted HeLa cell lysate (20 µg per reaction sample). The total reaction volume per condition was 20 µL using PCR tubes. For NUDT1 heat-control samples, the protein was denatured at 95 °C for 10 min prior to addition of the photocrosslinker probes. After addition of the respective probes (5 µM) the samples were incubated at room temperature for 30 min. For competition conditions samples were incubated with the parent compound (Ciprofloxacin or Levofloxacin) for 30 min at room temperature prior to probe addition. Subsequently, the samples were irradiated on ice for 5 min with UV light (365 nm for **P1** probes, *TL-D BLB 18 W, Philips, Amsterdam, The Netherlands*, 300 nm for **P2** probes, *LZC-UVB 8 W, Luzchem, Ottawa, Canada*). Click Master-mix (1 µL, 100 µM

TAMRA-azide, *Baseclick, Neuried, Germany, Cat# BCFA-008-10*, 500 µM BTTAA ligand, *Jena Bioscience, Jena, Germany, CLK-067*, 1 mM $CuSO_4$) was added and the CuAAC reaction started by addition of 920 nL of aq. sodium ascorbate (c_{end}= 2 mM). The reaction was incubated in the dark at room temperature for 1 h and subsequently, 2× SDS loading buffer (23 µL) was added, the samples denatured at 95 °C for 10 min and subjected to SDS-PAGE. Fluorescence scanning was performed using a *Las-3000* fluorescence darkbox (*Fujifilm, Minato, Japan*) with a *Fujinon* VRF 43LMD Lens, 605DF40 filter and 520 nm EPI excitation wavelength and loading controls were developed with Coomassie stain afterwards.

ADAL1 target validation efforts

Recombinant ADAL1 expression & purification were performed similar to published procedures with minor modifications.[21] Full length ADAL1 gene synthesis (see insert sequence, not codon-optimized) and cloning into pET28a(+) using restriction sites NdeI and XhoI was ordered from *TWIST Bioscience* (*South San Francisco, USA*). Insert sequence:

ATGATAGAGGCAGAAGAGCAACAGCCTTGCAAGACAGACTTCTATTCTGAATTGCCAAAAGTGGAACTTCATG
CCCACTTGAATGGATCCATTAGTTCTCATACCATGAAGAAATTAATAGCCCAGAAGCCAGATCTTAAAATCCAC
GATCAGATGACTGTGATTGACAAGGGAAAGAAAAGAACTTTGGAAGAATGTTTCCAGATGTTTCAAACTATTC
ATCAGCTTACTAGTAGCCCTGAAGATATTCTAATGGTCACAAAAGATGTCATAAAAGAATTTGCAGATGACGG
CGTCAAGTACCTGGAACTAAGGAGCACACCCAGAAGAGAAAATGCTACTGGAATGACTAAAAAGACTTATGT
GGAATCTATACTTGAAGGTATAAAACAGTCCAAACAAGAAAACTTGGACATTGATGTTAGGTATTTGATAGCA
GTTGACAGAAGAGGTGGCCCTTTAGTAGCCAAGGAGACTGTAAAACTTGCCGAGGAGTTCTTCCTTTCTACTG
AGGGTACAGTTCTTGGCCTTGACCTCAGTGGAGACCCTACTGTAGGACAAGCAAAAGACTTCTTGGAACCTCTT
TTAGAAGCTAAGAAAGCAGGTCTGAAGTTAGCATTGCATCTTTCAGAGATTCCAAACCAAAAAAAAGAAACAC
AAATACTCCTGGATCTGCTTCCTGACAGAATCGGGCATGGAACATTTCTCAACTCCGGTGAGGGAGGATCCCT
GGATCTGGTGGACTTTGTGAGGCAACATCGGATACCACTGGAACTCTGTTTGACCTCAAACGTCAAAAGTCAG
ACAGTTCCATCTTATGACCAGCACCATTTCGGATTCTGGTACAGCATTGCCCATCCTTCTGTGATCTGTACTGAT
GATAAGGGTGTTTTTGCAACACACCTTTCTCAAGAGTACCAGCTGGCAGCTGAAACATTTAATTTGACCCAGTC
TCAGGTGTGGGATCTGTCTTATGAATCCATCAACTACATCTTTGCTTCTGACAGCACCAGATCTGAACTGAGGA
AGAAATGGAATCACCTGAAGCCCAGAGTGTTACATATTTAA

The plasmid was transformed into chemically competent *E. coli* BL21 (DE3) and expression clones verified by sequencing. An overnight culture of the expression strain (LB + 1:1000 kanamycin) was grown and 1 L flasks (LB medium + 1:1000 kanamycin) were inoculated 1:100 and incubated at 37 °C, 200 rpm until OD_{600} = 0.5 – 0.6. The flasks were cooled on ice for 15 min, the overexpression induced by addition of IPTG (500 µM) and incubated at 20 °C, 200 rpm for 20 h. The bacteria were harvested, the pellet of 2 L culture combined, washed with cold PBS and stored at –80 °C. The pellet was resuspended in 30 mL Buffer A (50 mM Tris pH 8.0, 300 mM NaCl, 5 mM β-mercaptoethanol, 0.1% (v/v) NP40, 10% (v/v) glycerol and 20 mM imidazole) and lysis was performed by sonification (7 min 30% intensity, 3 min 70%, 7 min 30%, 3 min 70%, pulse 5, *Bandelin SONOPULS* equipped with *Bandelin UW2070* and *MS73* lance, *Bandelin electronic, Berlin Germany*). The lysate was cleared by centrifugation (40,000 ×g, 4 °C, 30 min) and filtered through a *Whatman* filter (0.45 µm PVDF w/GMF,

Cytiva, Marlborough, USA, Cat# 6818-1304). Nickel-affinity chromatography purification was performed with *Äkta Pure* system (*GE Healthcare, Chicago, USA*) using an HisTrap HP (5 mL, *Cytiva, Marlborough, USA, Cat# 17-5248*) column equilibrated in buffer A. The filtered lysate was applied *via* the sample pump and subsequently the column was washed for 15 column volumes (CV) with buffer A, before gradient elution with buffer B (50 mM Tris pH 8.0, 300 mM NaCl, 5 mM β-mercaptoethanol, 0.1% (v/v) NP40, 10% (v/v) glycerol and 400 mM imidazole) from 0% to 100% buffer B over for 25 CV. ADAL1 containing fractions were identified *via* SDS-PAGE, pooled and dialyzed in buffer 1 (20 mM Tris pH 8.0, 1 mM DTT, 10% (v/v) glycerol) in a dialysis membrane (*Carl Roth, Karlsruhe, Germany, Cat# E659.1*) overnight at 4 °C. Anion exchange chromatography (*Resource Q 1 mL*, *GE Healthcare, Chicago, USA, Cat# 17-1177-01*) was performed for further purification. The dialyzed sample was applied by sample pump, washed with buffer 1 for 5 CV and eluted with a linear gradient to 100% buffer 2 (20 mM Tris pH 8.0, 1 mM DTT, 10% (v/v) glycerol, 1 M NaCl) over 20 CV. Fractions containing non-aggregated and active ADAL1 were concentrated using a 10 kDa MWCO *Amicon* (*Ultra-15, Merck Millipore, Darmstadt, Germany, Cat# UFC901024*) to a concentration of 42 µM and snap-frozen in liquid nitrogen in aliquots to be stored at –80 °C. The identity and activity of the recombinant protein was checked SDS-PAGE (approx. 42 kDa) and *in vitro* activity assays.

ADAL1 activity assay

The ADAL1 in vitro activity assay was performed as previously reported.[21] Briefly, the conversion of N^6-methyl-AMP (*Cayman Chemical*, *Ann Arbor, USA*, Cat# 23382) to inosine by ADAL1 was monitored by the shift of the absorbance maxima of educt (265 nm) and product (250 nm). The FQs Ciprofloxacin and Levofloxacin were tested in the concentrations 500 µM, 400 µM, 300 µM, 200 µM and 100 µM. In a *UVStar* (*Greiner, Kremsmünster, Austria, Cat# 655801*) 96-well plate 1 µL of compounds or vehicle controls (1:100 stocks, FQs in 0.1 N HCl) were added in triplicates into the wells and 98 µL of enzyme master mix (100 nM ADAL1 in 50 mM potassium phosphate buffer pH 6.7, 2 mM DTT and 100 µg/mL BSA) was added. Conditions with master mix lacking NUDT1 were added as control. The plate was incubated for 30 min at 37 °C and subsequently the enzyme reaction started by addition of the substrate N^6-methyl-AMP (1 µL of 20 mM stock in PBS). The absorbance at 270 nm was continuously measured using a *Infinite M200 Pro (Tecan, Männedorf, Switzerland)* plate reader. The initial slopes of the absorbance readings were calculated *via* linear regression using *GraphPad Prism* (version 9). The residual activity of compound treated protein was determined in comparison to vehicle-treated control samples and ADAL1-lacking control samples which were normalized to 100% and 0% activity, respectively. The experiments were repeated in at least two independent experiments.

Lysosomal immunocytochemistry/ cell imaging

HeLa cells were seeded in *ibiTreat* 8-well µ-slides (*ibidi, Munich, Germany*) at a density of 0.03 million cells per well and grown overnight. Next day, cells were labelled with 20 µM Cipro P2 probe or DMSO for 1 h. Then cells wells were washed with PBS + Ca^{2+}/Mg^{2+} for 10 min before fixing the cells with 4% PFA in PBS for 15 min at RT. Afterwards wells were washed with PBS for 5 min and permeabilized with 0.1% Triton X-100 in PBS for 10 min at RT. After another 5 min washing step cells were blocked with 5% BSA in PBS for 1 h at RT and then again washed for 5 min. A fresh click chemistry mix was prepared consisting of the following components: 1 mM $CuSO_4$, 1 mM TCEP, 100 µM TBTA and 10 µM Alexa Fluor™ azide (*Thermo Fisher Scientific, Waltham, USA, Cat # A10277*) in PBS. 200 µL click mix were added per well and slide was incubated for 2h in the dark at RT while vigorously shaking. Then cells were washed thrice with PBS and incubated with a primary anti-LAMP1 antibody (*Developmental Studies Hybridomy Bank, Iowa City, USA, ID H4A3*) overnight at 4 °C while gently shaking. On the next day, wells were washed thrice with PBS for 5 min in the dark and subsequently incubated with an Alexa Fluor™ 488 labeled anti-mouse secondary antibody (*Thermo Fisher Scientific, Waltham, USA, Cat# A-11001*) and counter-stained with *Hoechst 33342* (*Sigma Aldrich, St. Louis, USA*) for 1 h at RT in the dark while gently shaking. After another 3 x 5 min washing steps with PBS, wells were mounted (*FluorSave™, Merck Millipore, Darmstadt, Germany*), covered with a glass slip, and imaged with a *Leica TCS SP8* confocal microscope (*Leica Microsystems, Wetzlar, Germany*). 50 cells per repetition (three independent experiments) were analyzed and co-localization of the green and red channels were quantified with the help of the *Coloc2 ImageJ* plugin and delineated as mean Pearson's r value.

Statistical information

Proteomics experiments were performed with 3-4 replicates per condition. Regulation or enrichment analyses were performed by two-sided two-sample *t*-tests. As significance threshold a negative logarithmic *P*-value of 1.3 (= FDR of 0.05) was set. For whole proteome, a $\log_2$ regulation of ±1 (= fold-change of ±2) and for enrichment experiments $\log_2 \geq 2$ (= fold-change of ≥ 4) was used. For details see the proteomics sections in the supporting information. The TPP analysis was performed with two replicates per conditions and analyzed as described by *Franken et al*. For details refer to the TPP section in the supporting information.[5]

The NUDT1 activity assays were performed with triplicates per condition and the shown graphs representative of 3 independent experiments. The statistical relevance is based on one-way ANOVAs with Dunnett´s multiple comparison tests (Ciprofloxacin: F = 100.8, DF = 17; Levofloxacin: F = 40.6, DF = 17; (S)-crizotinib: F = 42.83, DF = 14). The ADAL1 activity assays were performed with triplicates per condition and the shown graphs are representative of 2 independent experiments. The statistical relevance is based on one-way ANOVAs with Dunnett´s multiple comparison tests (Ciprofloxacin: F =

90.87, DF = 20; Levofloxacin: F = 146.9, DF = 20). The IDH2 activity assays were performed with 4 replicates per condition and the shown graphs representative of 3 independent experiments. The statistical relevance is based on one-way ANOVAs with Dunnett´s multiple comparison tests (Ciprofloxacin: F = 136.1, DF = 23; Levofloxacin: F = 335.3, DF = 23; Enadisenib: F = 699.2, DF = 19). For the mitochondrial NADPH levels the average and SD of three independent experiments with three replicates per condition is plotted. The statistical relevance is in the plot after 6 h is based on an unpaired two-tailed *t*-test (t = 9.316, DF = 4). The statistical relevance in the plot after 72 h is based on a one-way ANOVA with Dunnett´s multiple comparison tests (F = 7.454, DF = 8).

Synthetic Procedures

General Working Methods

All air or water sensitive reactions were carried out under inert-gas atmosphere (Argon) in oven-dried glassware with anhydrous solvents utilizing standard Schlenk-techniques unless noted otherwise. Reagents and solvents were purchased from *Sigma-Aldrich*, *Alfa Aesar*, *Acros Organics*, *TCI Europe* or *Merck* and were of reagent grade or better and used without further purification. All reactions were magnetically stirred, and temperatures measured externally. Yields refer to isolated homogeneous and spectroscopically pure materials. Sensitive samples were stored in a freezer (–20 °C). Solvents were removed *in vacuo* at 42 °C. Reactions and fractions of flash column chromatography were monitored by qualitative thin layer chromatography /TLC) on aluminium-baked TLC silica gel plates (TLC Silica gel 60 F_{254}, *Merck KGaA*). In order to visualize the substances on the plates UV light (λ_{max} = 254 nm) and staining solutions, such as $KMnO_4$, CAM and *p*-anisaldehyde, were used. Flash column chromatography (FCC) was performed with slurry packed silica gel (40-63 µm) from VWR and elution solvents distilled prior to use. NMR spectra were recorded using an internal deuterium lock at ambient probe temperature on a Bruker instruments (300 MHz, 400 MHz or 500 MHz). The ^{1}H NMR chemical shifts are reported in ppm related to the chemical shift of TMS and calibrated to the residual protic solvent peaks of $CDCl_3$ (7.26 ppm) and DMSO-d_6 (2.50 ppm). ^{13}C NMR shifts were calibrated to the center of the multiplet signal of the residual solvent peak ($CDCl_3$ = 77.16 ppm, DMSO-d_6 = 39.52 ppm) and quoted to the nearest 0.1 ppm. ^{1}H NMR spectroscopic data are reported as follows: Chemical shift to the nearest 0.01 ppm (multipilicity, coupling constants *J*, integration intensity). For the multiplicities the abbreviations s (singlet), br s (broad singlet), d (doublet), t (triplet), q (quartet) and m (multiplet) have been used. High-resolution mass spectrometry (HRMS) measurements were recorded with a *Thermo Scientific* LTQ FT Ultra or LTQ Orbitrap instrument with ESI ionization.

3-(but-3-yn-1-yl)-3-(2-iodoethyl)-3*H*-diazirine (MPC-I)

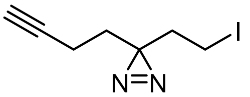

$C_8H_9IN_2$
M = 248.07 g/mol
MPC-I

The iodo-derivative of the minimalist photocrosslinker was synthesized according to literature procedures by *Li et al.* starting from the commercially available alcohol derivative. Analytical data were in accordance with the literature values.[22] – **^{1}H-NMR** (500 MHz, $CDCl_3$): δ[ppm] = 2.89 (t, *J* = 7.6 Hz, 2H), 2.12 (t, *J* = 7.6 Hz, 2H), 2.00-2.05 (m, 3H), 1.69 (t, *J* = 7.2 Hz, 2H).

7-(4-(2-(3-(but-3-yn-1-yl)-3*H*-diazirin-3-yl)ethyl)piperazin-1-yl)-1-cyclopropyl-6-fluoro-4-oxo-1,4-dihydroquinoline-3-carboxylic acid (Cipro P1)

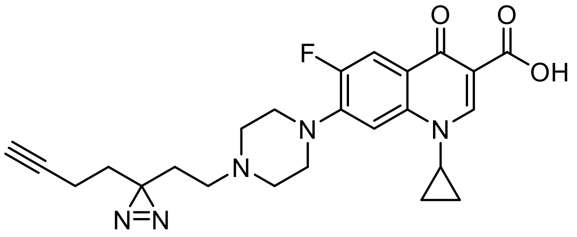

$C_{24}H_{26}FN_5O_3$
M = 451.50 g/mol
Cipro P1

To a suspension of ciprofloxacin (54 mg, 0.165 mmol, 1.0 eq.) and Cs2CO3 (194 mg, 0.594 mmol, 3.0 eq.) in H2O (1.5 mL) was added a solution of **MPC-I** (49 mg, 0.198 mmol, 1.2 eq.) in MeCN (1.5 mL). The reaction was heated to 70 °C in a sealed vial and stirred overnight. The mixture was concentrated in vacuo and the residue dissolved in H2O and EtOAc. After extraction with EtOAC (3 × 10 mL) and drying of the pooled organic phases over MgSO4, the solvent was removed in vacuo and the crude product purified by reverse phase preparative HPLC (Gradient 2% to 70% MeCN in H2O) to afford the title compound as white solid (25 mg, 0.055 mmol, 33%) after lyophilization. – **^{1}H-NMR** (500 MHz, DMSO-d6): δ[ppm] = 15.11 (br s, 1H), 8.69 (s, 1H), 7.97 (d, *J* = 13.0 Hz, 1H), 7.60 (d, *J* = 7.4 Hz, 1H), 3.84 (td, *J* = 7.2, 3.7 Hz, 1H), 3.40 – 3.14 (br m, 10H), 2.89 (t, *J* = 2.7 Hz, 1H), 2.05 (td, *J* = 7.3, 2.7 Hz, 2H), 1.83 (br s, 2H), 1.66 (t, *J* = 7.4 Hz, 2H), 1.32 (q, *J* = 7.1, 6.3 Hz, 2H), 1.22 – 1.15 (m, 2H). – **^{13}C-NMR** (125 MHz, DMSO-d6): δ[ppm] = 176.5, 176.4, 165.9, 153.9, 151.9, 148.3, 143.7, 139.1, 119.5, 119.4, 111.4, 111.2, 107.1, 106.9, 83.0, 72.2, 50.7, 50.3, 46.6, 36.0, 30.9, 27.3, 26.7, 12.7, 7.7. – **HR-MS** (ESI) *m/z* for C24H27FN5O3+ [(M+H)+]: calcd. 452.2092, found 452.2093. – In the ^{1}H NMR the broad multiplet signal

3.40 – 3.14 (br m, 10H) is due to the aliphatic positions of and next to the piperazine, the signals partially overlay with residual water in DMSO-d6. Complexity arises from coupling with fluorine. Additional signals due to presence of TFA after the preparative HPLC purification are detected in the 1H NMR (δ = 10.05 (br s)) and the 13C NMR (δ[ppm] = 158.5, 158.3, 158.0, 157.8, 118.4, 116.0).

(*S*)-9-fluoro-3,7-dihydro-3-methyl-7-oxo-10-(piperazin-1-yl)-2*H*-[1,4]oxazino[2,3,4-*ij*]quinoline-6-carboxylic acid (1)

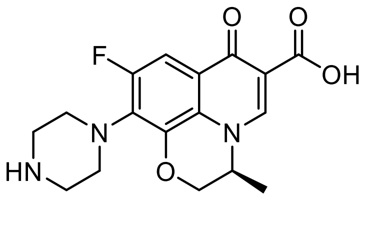

$C_{17}H_{18}FN_3O_4$
M = 347.35 g/mol
1

To a solution of levofloxacin Q-acid (400 mg, 1.42 mmol, 1.0 eq.) in MeCN (15 mL) piperazine (167 µL, 2.13 mmol, 1.5 eq.) was added and the mixture was stirred for 30 min at room temperature. After addition of triethylamine (591 µL, 4.27 mmol, 3.0 eq.) the resulting reaction mixture was heated to reflux for 12 h. The reaction was cooled to room temperature and the precipitate was isolated by filtration, washed with MeCN, refluxed in EtOH and cooled to room temperature overnight. The precipitate was removed by filtration and the supernatant concentrated *in vacuo* to obtain the title compound (245 mg, 705 µmol, 50%) as a green-white solid. The spectroscopic data are in accordance with the literature.[23] – **^{1}H-NMR** (300 MHz, DMSO-d^6): δ[ppm] = 8.95 (s, 1H), 7.57 (d, J = 12.5 Hz, 1H), 4.91 (d, J = 6.6 Hz, 1H), 4.57 (d, J = 11.3 Hz, 1H), 4.36 (d, J = 11.3 Hz, 1H), 3.22 (d, J = 6.1 Hz, 4H), 2.81 (t, J = 5.0 Hz, 4H), 1.44 (d, J = 6.8 Hz, 3H).

(*S*)-10-(4-(2-(3-(but-3-yn-1-yl)-3*H*-diazirin-3-yl)ethyl)piperazin-1-yl)-9-fluoro-3-methyl-7-oxo-2,3-dihydro-7*H*-[1,4]oxazino[2,3,4-*ij*]quinoline-6-carboxylic acid (Levo P1)

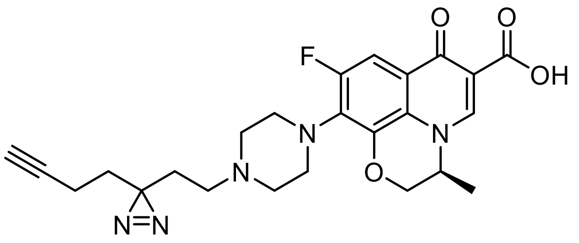

$C_{24}H_{26}FN_5O_4$
M = 467.50 g/mol
Levo P1

To a solution of **1** (50 mg, 0.14 mmol, 1.0 eq.) in H2O (3 mL) CsCO3 (140 mg, 0.43 mmol, 3.0 eq.) was added. Then minimal **MPC-I** (43 mg, 0.17 mmol, 1.2 eq.) in MeCN (3 mL) was added and the mixture was stirred overnight at 70 °C under argon atmosphere. The reaction mixture was concentrated *in vacuo* to remove MeCN and further lyophilized to remove the water. The crude product was purified by flash column chromatography (5% to 10% MeOH in dichloromethane) and additional preparative HPLC to afford the title compound (47 mg, 0.10 mmol, 70%) as white solid after lyophilization. – **^{1}H-NMR** (400 MHz, CDCl3): δ[ppm] = 15.10 (s, 1H), 9.00 (s, 1H), 7.62 (d, *J* = 12.0 Hz, 1H), 4.99 – 4.90 (m, 1H), 4.59 (dd, *J* = 11.5, 1.8 Hz, 1H), 4.40 (dd, *J* = 11.5, 2.5 Hz, 1H), 3.62 – 3.45 (m, 6H), 3.23 – 3.07 (m, 4H), 2.89 (t, *J* = 2.7 Hz, 1H), 2.06 (td, *J* = 7.4, 2.7 Hz, 2H), 1.93 – 1.80 (m, 2H), 1.65 (t, *J* = 7.4 Hz, 2H), 1.45 (d, *J* = 6.7 Hz, 3H). – **^{13}C-NMR** (125 MHz, DMSO-d6): δ[ppm] = 176.4, 176.4, 165.9, 156.6, 154.1, 146.4, 140.7, 140.7, 130.3, 130.2, 124.7, 120.7, 120.6, 106.9, 103.4, 103.1, 82.9, 72.0, 68.3, 54.8, 51.7, 50.3, 47.1, 30.9, 27.1, 26.5, 17.9, 12.7. – **HR-MS** (ESI) *m/z* for $C_{24}H_{27}FN_5O_4^+$ [(M+H)$^+$]: calcd. 468.2042, found 468.2036. – Complexity arises from coupling with fluorine. Additional signals due to presence of TFA after the reparative HPLC purification are detected in the ^{1}H NMR (δ = 10.05 (br s)) and the ^{13}C NMR.

N-((3H-diazirin-3-yl)methyl)prop-2-yn-1-amine TFA salt (BCP)

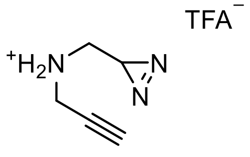

$C_5H_7N_3$ • TFA
M = 223.15 g/mol
BCP

The TFA salt of the branched photocrosslinker **BCP** was synthesized according to published procedures by *Conway et al.* and analytical data were in accordance with the literature values.[24] – **^{1}H-NMR** (400 MHz, $CDCl_3$): δ[ppm] = 4.06 (d, J = 2.6 Hz, 2H), 3.11 (d, J = 5.2 Hz, 2H), 2.60 (t, J = 2.6 Hz, 1H), 1.37 (t, J = 5.2 Hz, 1H).

7-[4-*tert*-butoxycarbonyl-piperazin-1-yl]-1-cyclopropyl-6-fluoro-1,4-dihydro-4-oxo-quinoline-3-carboxylic acid (2)

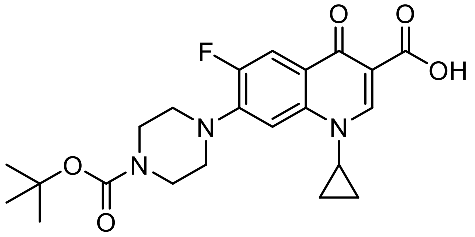

$C_{22}H_{26}FN_3O_5$
M = 431.46 g/mol
2

To a solution of ciprofloxacin (500 mg, 1.51 mmol, 1.0 eq.) in H_2O (4 mL) and dioxane (4 mL) NaOH (90.5 mg, 2.26 mmol, 1.5 eq.) was added. The mixture was cooled to 0°C and di-*tert*-butyl dicarbonate (494 mg, 2.26 mmol, 1.5 eq.) was added. After stirring for 5 min at 0 °C the reaction mixture was stirred for 3 h at room temperature. The solvent was removed *in vacuo*, acetone (30 mL) was added and the formed precipitate was filtered off. The filtrate was extracted with DCM (3 × 30 mL), the pooled organic phases washed with brine (50 mL), dried over $MgSO_4$, and concentrated. The residue was combined with the precipitate, providing compound **2** (587 mg, 1.36 mmol, 90%). The spectroscopic data are in accordance with literature values.[25] – **^{1}H-NMR** (500 MHz, $CDCl_3$): δ[ppm] = 8.79 (s, 1H), 8.06 (d, J = 12.8 Hz, 1H), 7.41 (d, J = 6.9 Hz, 1H), 3.68 (t, J = 5.0 Hz, 4H), 3.54 (s, 1H), 3.30 (t, J = 5.0 Hz, 4H), 1.50 (s, 9H), 1.40 (d, J = 5.6 Hz, 2H), 1.21 (s, 2H).

N-((3H-diazirin-3-yl)methyl)-1-cyclopropyl-6-fluoro-4-oxo-7-(piperazin-1-yl)-N-(prop-2-yn-1-yl)-1,4-dihydroquinoline-3-carboxamide (Cipro P2)

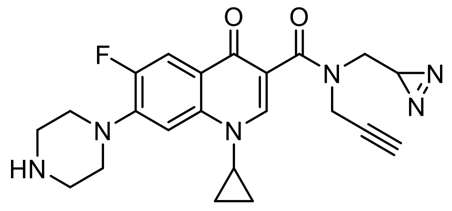

$C_{22}H_{23}FN_6O_2$
M = 422.46 g/mol
Cipro P2

To a solution of **2** (100 mg, 0.23 mmol, 1.0 eq.) in DCM (20 mL) HBTU (220 mg, 0.58 mmol, 2.5 eq.) and DMAP (cat.) were added. After stirring for 10 min at room temperature, DIPEA (202 µL, 1.16 mmol, 5.0 eq.) and **BCP** (32.9 mg, 0.30 mmol, 1.3 eq.) were added. The reaction mixture was heated to reflux for 3 h. The reaction was concentrated *in vacuo* and purified by column chromatography (MeOH:DCM = 2:98 to 4:96) to afford the amine coupling product as a light-yellow solid that was directly subjected to Boc-deprotection conditions. The solid was dissolved in CH_2Cl_2 (3 mL) and 2 mL TFA (50% (v/v) in CH_2Cl_2) was added dropwise at 0 °C. After stirring for 2 h at room temperature, the solvent was removed *in vacuo* and the crude product was purified by preparative HPLC (2-70% MeCN in H_2O over 26 min), affording compound **Cipro P2** (45.4 mg, 0.11 mmol, 46% over 2 steps) as a colorless solid. – **^{1}H-NMR** (500 MHz, CDCl3): δ[ppm] = 9.96 (s, 2H), 8.08 (s, 1H), 7.95 (d, J = 12.7 Hz, 1H), 7.36 (d, J = 6.9 Hz, 1H), 4.34 (d, J = 153.2 Hz, 2H), 3.56 – 3.44 (m, 8H), 3.32 (s, 1H), 2.56 – 2.15 (m, 3H), 1.33 (d, J = 6.9 Hz, 3H), 1.18 – 1.11 (m, 2H). – **^{13}C-NMR** (125 MHz, CDCl3): δ[ppm] = 172.4, 172.2, 167.5, 154.3, 152.3, 144.9, 143.6, 143.5, 138.5, 122.6, 122.6, 113.1, 113.0, 105.8, 78.5, 72.7, 47.0, 43.6, 34.7, 19.9, 8.3, 1.1. – **HR-MS** (ESI) *m/z* for $C_{22}H_{24}FN_6O_2^+$ [(M+H)$^+$]: calcd. 423.1939, found 423.1939. – Complexity arises from coupling with fluorine. Additional signals due to presence of TFA after the preparative HPLC purification are detected in the ^{1}H NMR and the ^{13}C NMR.

(S)-N-((3H-diazirin-3-yl)methyl)-9-fluoro-3-methyl-10-(4-methylpiperazin-1-yl)-7-oxo-N-(prop-2-yn-1-yl)-2,3-dihydro-7H-[1,4]oxazino[2,3,4-ij]quinoline-6-carboxamide (Levo P2)

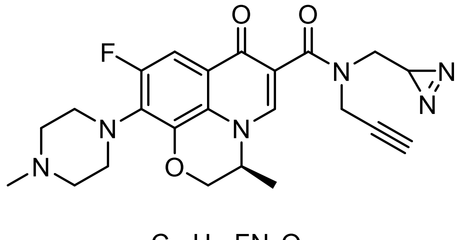

$C_{23}H_{25}FN_6O_3$
M = 452.49 g/mol
Levo P2

To a solution of levofloxacin (100 mg, 0.28 mmol, 1.0 eq.) in DCM (24 mL) HBTU (262 mg, 0.69 mmol, 2.5 eq.) and DMAP (cat.) were added. After stirring for 10 min at room temperature, DIPEA (242 µL, 1.38 mmol, 5.0 eq.) and **BCP** (39.3 mg, 0.36 mmol, 1.3 eq.) were added. The reaction mixture was heated to reflux for 3 h. Subsequently, the mixture was concentrated *in vacuo* and purified by preparative HPLC (2-70% MeCN in H_2O over 26 min) to obtain the product **Levo P2** (43.2 mg, 0.96 mmol, 35%) as a brown oil. – **^{1}H-NMR** (400 MHz, CDCl3): δ[ppm] = 7.85 (s, 1H), 7.38 (t, *J* = 10.5 Hz, 1H), 4.46 (s, 1H), 4.40 – 4.31 (m, 3H), 4.22 – 4.01 (m, 1H), 3.54 (s, 4H), 3.40 (t, *J* = 19.5 Hz, 1H), 3.15 (s, 4H), 2.76 (s, 3H), 2.27 (t, *J* = 2.5 Hz, 1H), 1.98 (s, 2H), 1.49 (s, 3H), 1.32 – 1.21 (m, 1H). – **^{13}C-NMR** (125 MHz, CDCl3): δ[ppm] = 171.6, 167.4, 167.2, 156.3, 154.3, 142.7, 142.3, 140.2, 140.2, 129.7, 129.6, 124.4, 122.9, 116.6, 116.3, 104.6, 104.4, 104.3, 78.7, 78.4, 73.4, 72.6, 68.3, 54.6, 54.3, 48.9, 48.3, 45.7, 44.3, 39.6, 35.3, 20.0, 19.8, 18.4, 2.0. – **HR-MS** (ESI) *m/z* for $C_{23}H_{26}FN_6O_3^+$ [(M+H)$^+$]: calcd. 453.2045, found 453.2040. – Complexity arises from coupling with fluorine. Additional signals due to presence of TFA after the preparative HPLC purification are detected in the ^{1}H NMR and the ^{13}C NMR.

NMR spectra of probes

^{1}H NMR **Cipro P1** (500 MHz, DMSO-d6):

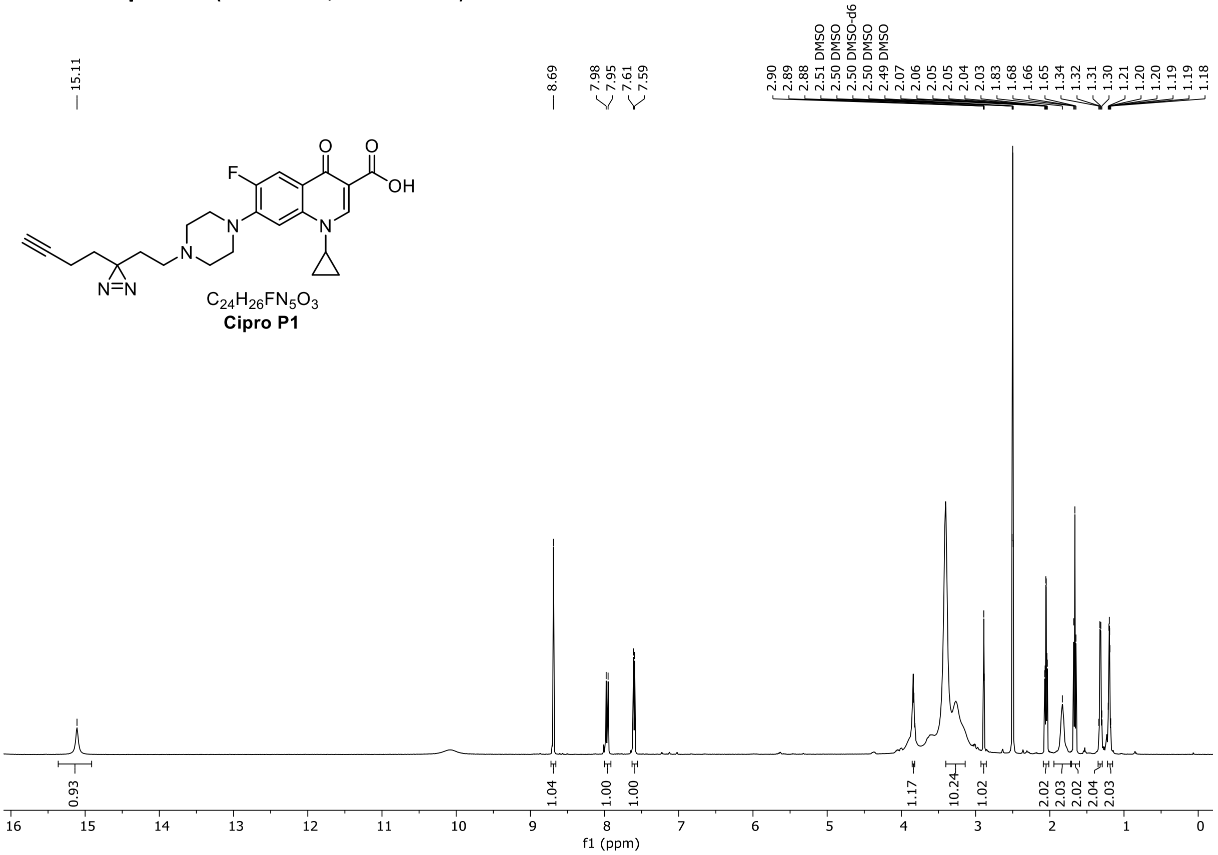

^{13}C NMR **Cipro P1** (125 MHz, DMSO-d6):

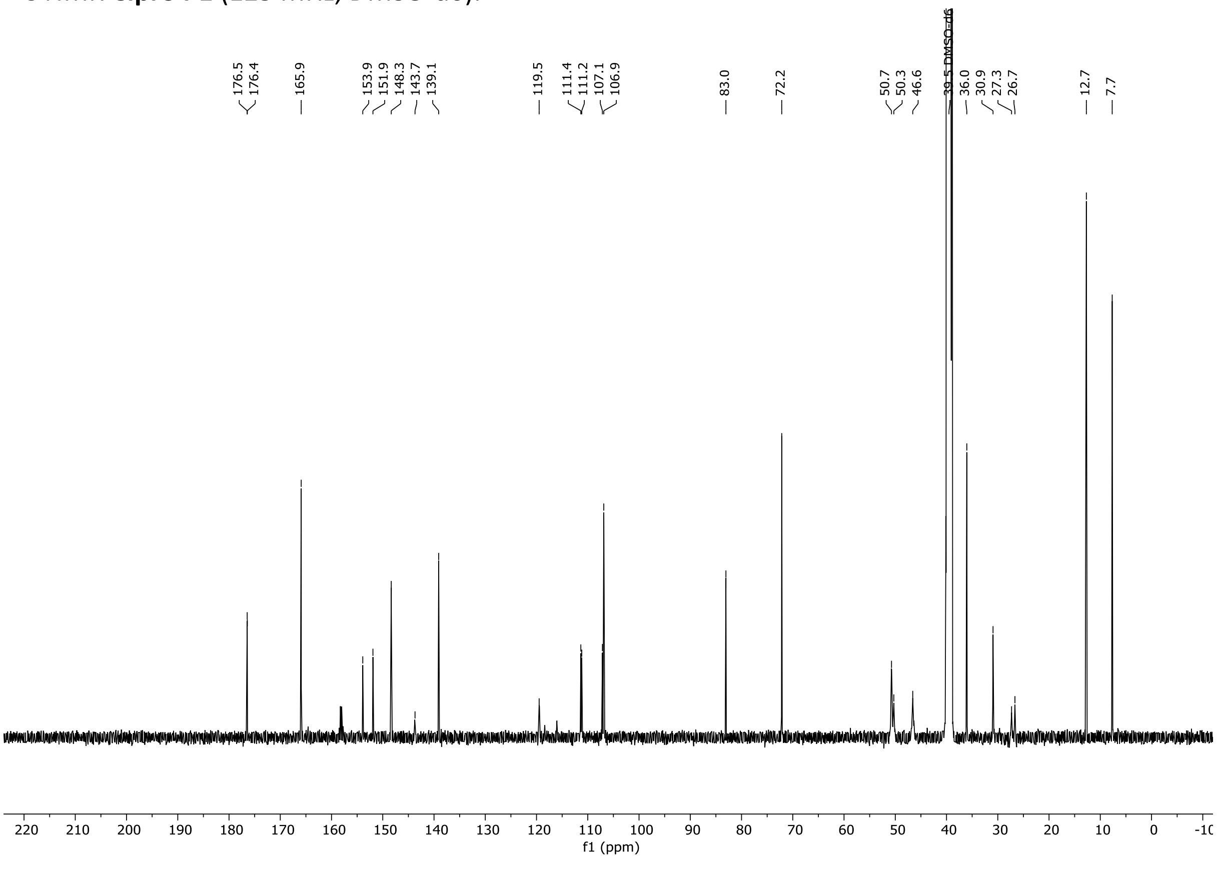

^{1}H NMR **Levo P1** (400 MHz, DMSO-d6):

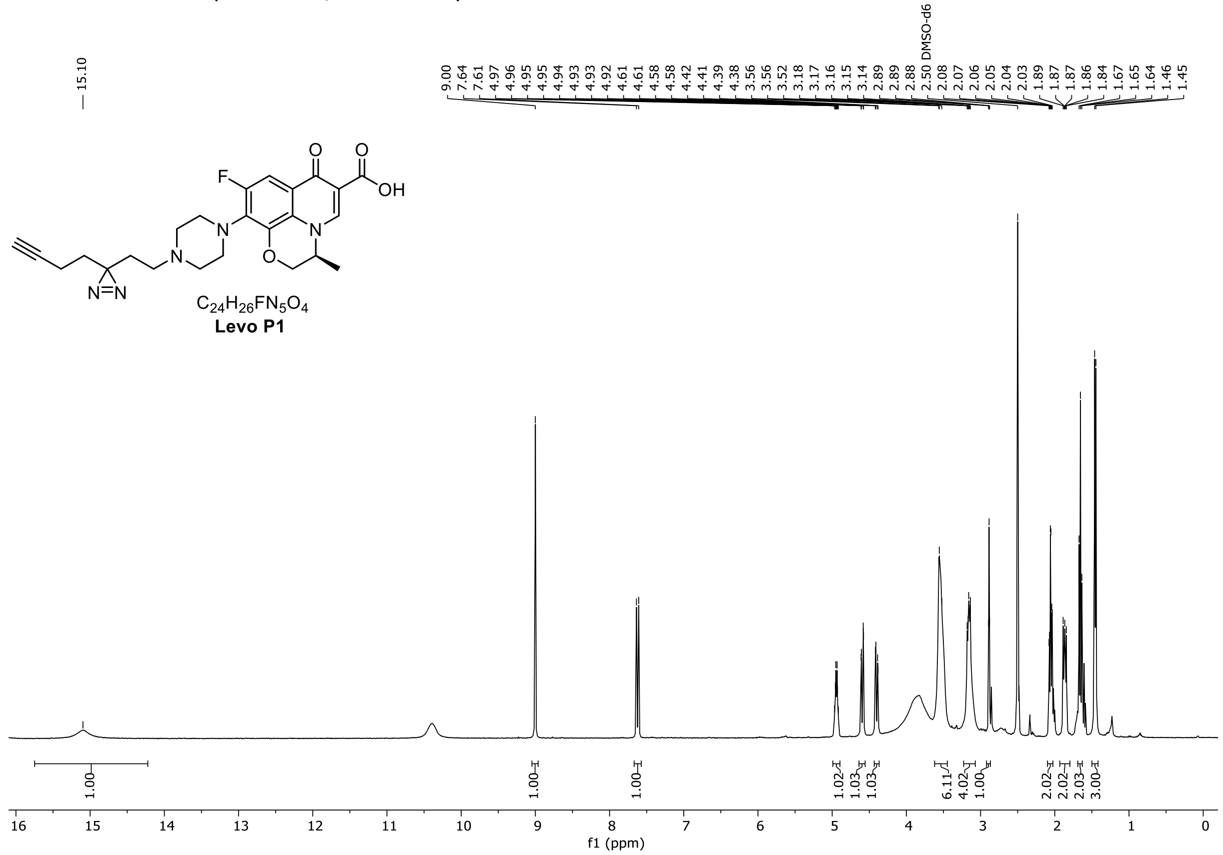

$C_{24}H_{26}FN_5O_4$
Levo P1

^{13}C NMR **Levo P1** (125 MHz, DMSO-d6):

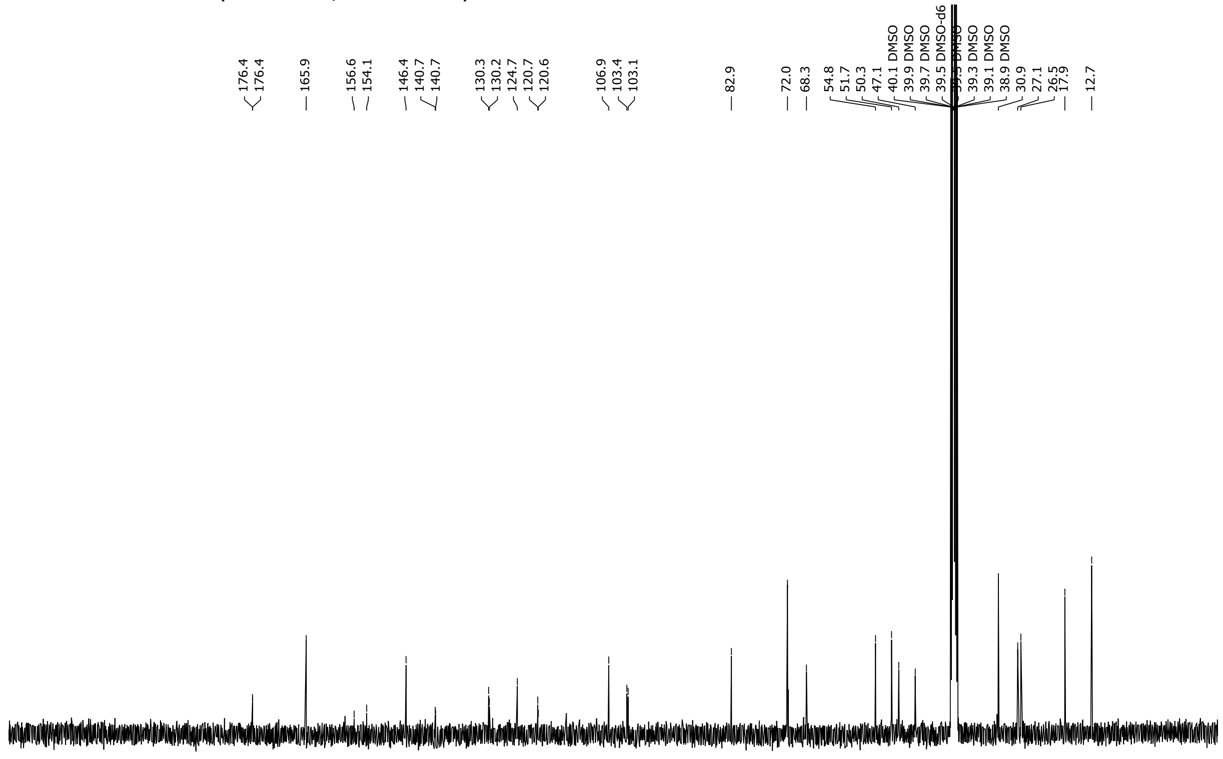

^{1}H NMR **Cipro P2** (500 MHz, $CDCl_3$):

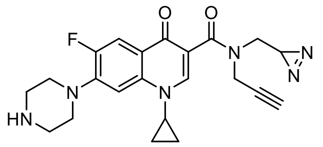

$C_{22}H_{23}FN_6O_2$
Cipro P2

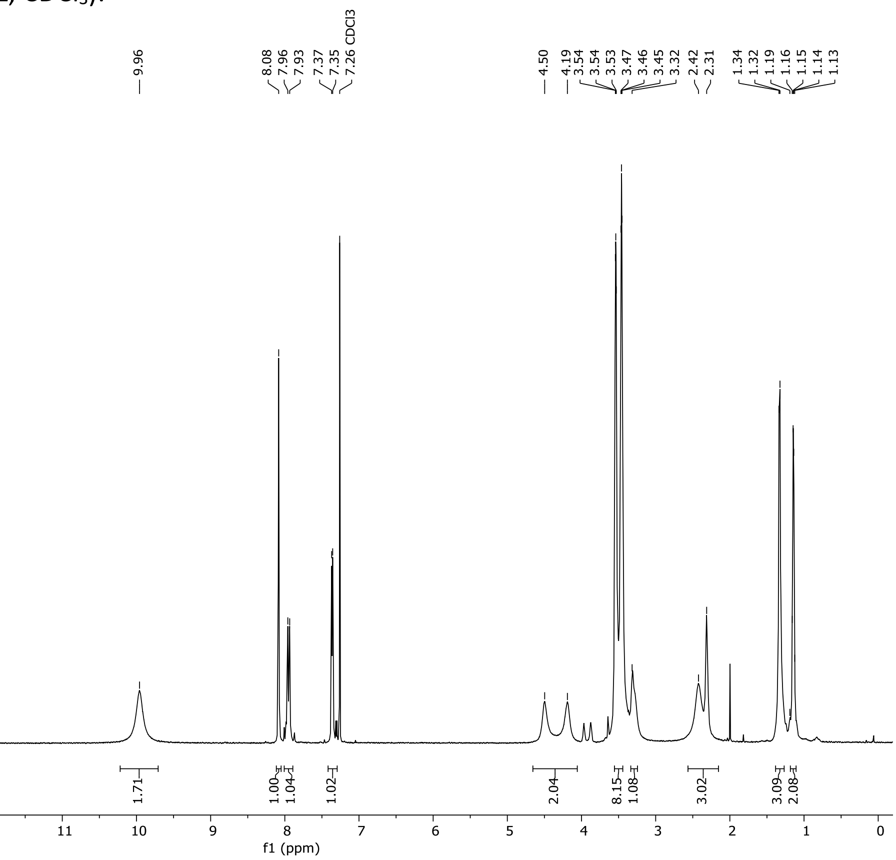

^{13}C NMR **Cipro P2** (125 MHz, $CDCl_3$):

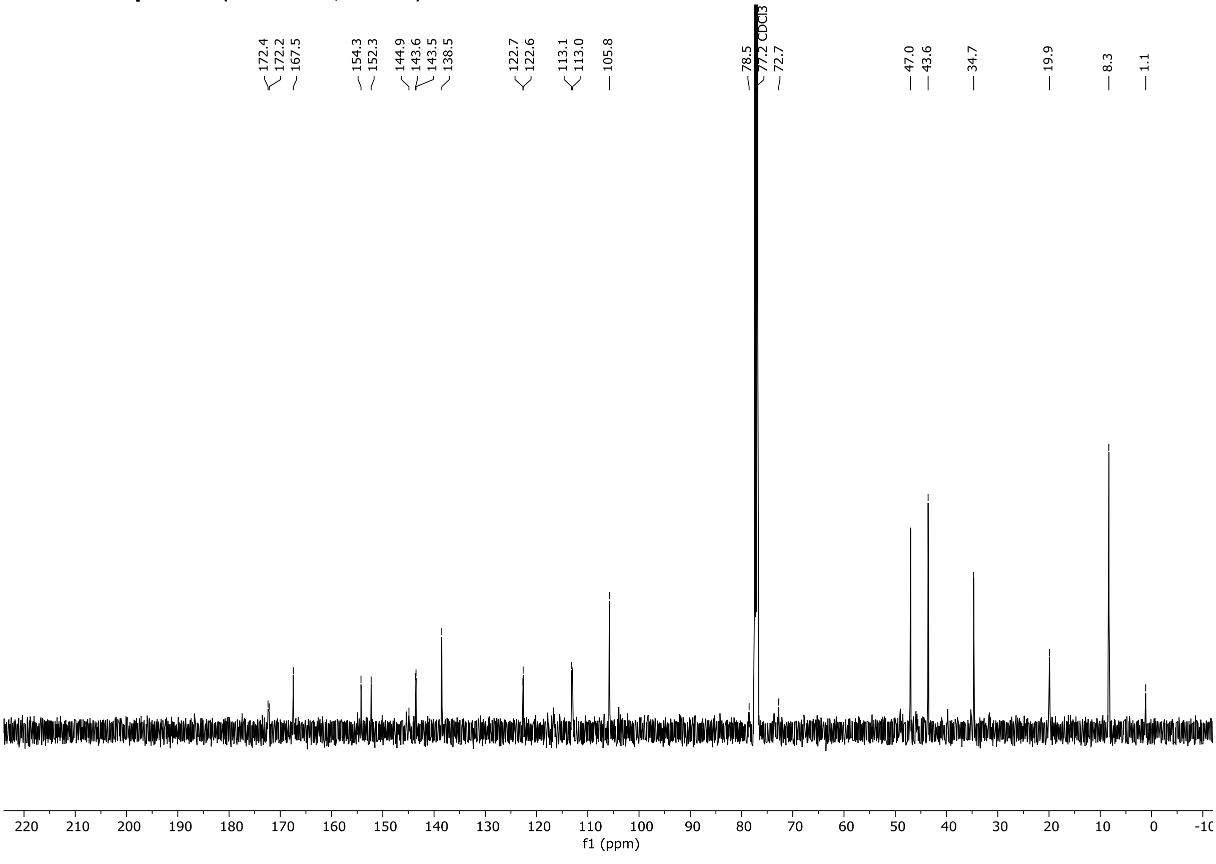

^{1}H NMR **Levo P2** (400 MHz, $CDCl_3$):

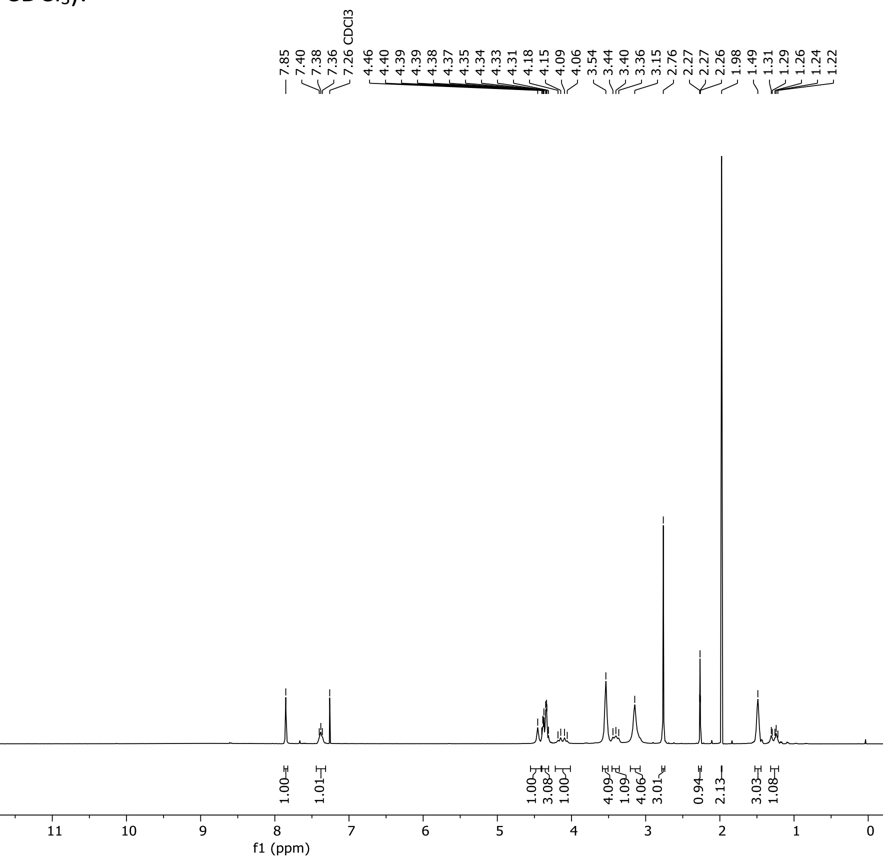

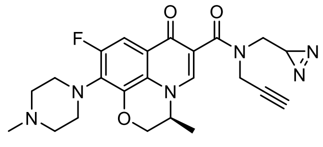

$C_{23}H_{25}FN_6O_3$
Levo P2

^{13}C NMR **Levo P2** (125 MHz, $CDCl_3$):

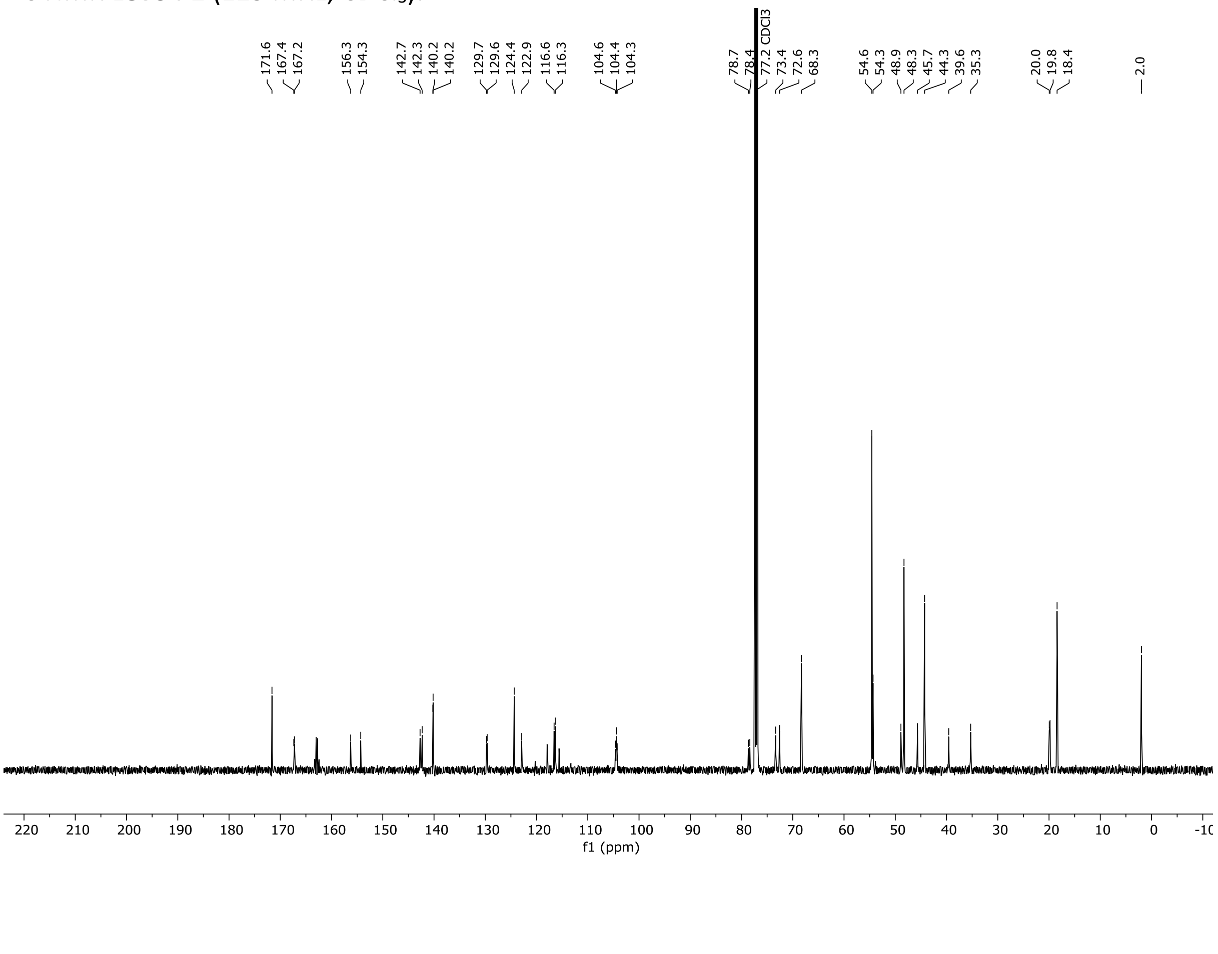

References:

1. Docheva, D. *et al.* Establishment of immortalized periodontal ligament progenitor cell line and its behavioural analysis on smooth and rough titanium surface. *Eur. Cells Mater.* **19**, 228–241 (2010).
2. Reinhardt, T., Lee, K. M., Niederegger, L., Hess, C. R. & Sieber, S. A. Indolin-2-one Nitroimidazole Antibiotics Exhibit an Unexpected Dual Mode of Action. *ACS Chem. Biol.* **17**, 3077–3085 (2022).
3. Wiśniewski, J. R., Zougman, A., Nagaraj, N. & Mann, M. Universal sample preparation method for proteome analysis. *Nat. Methods* **6**, 359–362 (2009).
4. Schum, D. *et al.* Screening Privileged Alkyl Guanidinium Motifs under Host- Mimicking Conditions Reveals a Novel Antibiotic with an Unconventional Mode of Action. *JACS Au* **4**, 3125–3134 (2024).
5. Franken, H. *et al.* Thermal proteome profiling for unbiased identification of direct and indirect drug targets using multiplexed quantitative mass spectrometry. *Nat. Protoc.* **10**, 1567–1593 (2015).
6. Demichev, V., Messner, C. B., Vernardis, S. I., Lilley, K. S. & Ralser, M. DIA-NN: neural networks and interference correction enable deep proteome coverage in high throughput. *Nat. Methods* **17**, 41–44 (2020).
7. Krämer, A., Green, J., Pollard, J. & Tugendreich, S. Causal analysis approaches in ingenuity pathway analysis. *Bioinformatics* **30**, 523–530 (2014).
8. Gersch, M. *et al.* Barrel-shaped ClpP Proteases Display Attenuated Cleavage Specificities. *ACS Chem. Biol.* **11**, 389–399 (2016).
9. Corydon, T. J. *et al.* A human homologue of Escherichia coli ClpP caseinolytic protease: Recombinant expression, intracellular processing and subcellular localization. *Biochem. J.* **331**, 309–316 (1998).
10. Nguyen, T., Gronauer, T. F., Nast-Kolb, T., Sieber, S. A. & Lang, K. Substrate Profiling of Mitochondrial Caseinolytic Protease P via a Site-Specific Photocrosslinking Approach. *Angew. Chemie Int. Ed.* **61**, e202111085 (2022).
11. Szczepanowska, K. *et al.* A salvage pathway maintains highly functional respiratory complex I. *Nat. Commun.* **11**, 1–18 (2020).
12. Yen, K. *et al.* AG-221, a First-in-Class Therapy Targeting Acute Myeloid Leukemia Harboring Oncogenic IDH2 Mutations. *Cancer Discov.* **7**, 478–493 (2017).
13. Salscheider, S. L. *et al.* AIFM1 is a component of the mitochondrial disulfide relay that drives complex I assembly through efficient import of NDUFS5. *EMBO J.* **41**, e110784 (2022).
14. Ran, F. A. *et al.* Genome engineering using the CRISPR-Cas9 system. *Nat. Protoc.* **8**, 2281–2308

(2013).

15. Montague, T. G., Cruz, J. M., Gagnon, J. A., Church, G. M. & Valen, E. CHOPCHOP: A CRISPR/Cas9 and TALEN web tool for genome editing. *Nucleic Acids Res.* **42**, 401–407 (2014).

16. Parker, C. G. *et al.* Ligand and Target Discovery by Fragment-Based Screening in Human Cells. *Cell* **168**, 527-541.e29 (2017).

17. Lum, K. M. *et al.* Mapping Protein Targets of Bioactive Small Molecules Using Lipid-Based Chemical Proteomics. *ACS Chem. Biol.* **12**, 2671–2681 (2017).

18. Zhou, W. *et al.* Potent and specific MTH1 inhibitors targeting gastric cancer. *Cell Death Dis.* **10**, 1–19 (2019).

19. Gad, H. *et al.* MTH1 inhibition eradicates cancer by preventing sanitation of the dNTP pool. *Nature* **508**, 215–221 (2014).

20. Yokoyama, T., Kitakami, R. & Mizuguchi, M. Discovery of a new class of MTH1 inhibitor by X-ray crystallographic screening. *Eur. J. Med. Chem.* **167**, 153–160 (2019).

21. Murakami, E. *et al.* Adenosine Deaminase-like Protein 1 (ADAL1): Characterization and Substrate Specificity in the Hydrolysis of N 6 - or O 6 -Substituted Purine or 2-Aminopurine Nucleoside Monophosphates. *J. Med. Chem.* **54**, 5902–5914 (2011).

22. Li, Z. *et al.* Design and synthesis of minimalist terminal alkyne-containing diazirine photo-crosslinkers and their incorporation into kinase inhibitors for cell- and tissue-based proteome profiling. *Angew. Chemie - Int. Ed.* **52**, 8551–8556 (2013).

23. Nakhaei, A., Ramezani, S., Shams-Najafi, S. J. & Farsinejad, S. Nano-Fe3O4@ZrO2-SO3H as Highly Efficient Recyclable Catalyst for the Green Synthesis of Fluoroquinolones. *Lett. Org. Chem.* **15**, 739–746 (2018).

24. Conway, L. P. *et al.* Evaluation of fully-functionalized diazirine tags for chemical proteomic applications. *Chem. Sci.* **12**, 7839–7847 (2021).

25. Alsayed, S. S. R., Lun, S., Payne, A., Bishai, W. R. & Gunosewoyo, H. Facile synthesis and antimycobacterial activity of isoniazid, pyrazinamide and ciprofloxacin derivatives. *Chem. Biol. Drug Des.* **97**, 1137–1150 (2021).
